# Pharmacological inhibition of MCL-1 disrupts mitochondrial cristae and depletes the human neural progenitor cell pool

**DOI:** 10.1242/jcs.265172

**Published:** 2026-10-15

**Authors:** Marina R. Hanna, James Costanzo, Madison Yarbrough, Melanie Gil, Matthew Murrow, Vivian Gama

**Affiliations:** ^1^ Vanderbilt University, Vanderbilt Brain Institute, Nashville, TN 37232, USA; ^2^ Vanderbilt University, Cell and Developmental Biology, Nashville, TN 37232, USA; ^3^ Vanderbilt University Medical Center, Biomedical Informatics, Nashville, TN 37232, USA; ^4^ Vanderbilt University, Vanderbilt Center for Stem Cell Biology, Nashville, TN 37232, USA

**Keywords:** Neural progenitor cells, Intermediate progenitors, MCL-1, Mitochondria, Cristae, ACSL1, MICOS, OPA1

## Abstract

Myeloid cell leukemia 1 (MCL-1) is a canonical anti-apoptotic protein crucial for early neurodevelopment. Loss of MCL-1 results in embryonic-lethal neurodevelopmental defects that cannot be rescued by other anti-apoptotic proteins of the B cell lymphoma 2 (BCL-2) family. Here, we pharmacologically inhibit MCL-1 in human neural progenitor cells and find non-apoptotic roles for MCL-1 in sustaining mitochondrial cristae integrity, fatty acid oxidation and neural progenitor identity. MCL-1 inhibition disrupts mitochondrial ultrastructure, leading to a swollen network with disorganized cristae and destabilized OPA1–MICOS machinery that fails to maintain inner mitochondrial membrane architecture. These structural defects are accompanied by displacement of ACSL1 from the outer mitochondrial membrane, impaired fatty acid oxidation, lipid droplet accumulation and reduced mitochondrial oxygen consumption, revealing a tight link between cristae architecture and metabolic competence. Mechanistically, MCL-1 acts through two coordinated functions: maintaining cristae integrity at the inner mitochondrial membrane and retaining ACSL1 at the outer mitochondrial membrane, both independently of caspase activation. Functionally, MCL-1 inhibition selectively depletes intermediate progenitor cells without affecting proliferation, indicating a direct role in lineage progression. Together, our findings position MCL-1 upstream of OPA1, MICOS and ACSL1, and identify it as a critical coordinator of cristae organization, lipid metabolism and neural progenitor fate. This establishes mitochondrial inner membrane architecture as an instructive determinant of human neurogenesis and highlights non-canonical MCL-1 functions as critical regulators of human brain development.

## Teaser

**Summary:** MCL-1, better known for blocking cell death, also shapes mitochondrial structure and metabolism to support intermediate progenitor pools, revealing a new role in neurogenesis.

## INTRODUCTION

Neurodevelopment relies on the precise coordination of cellular processes that determine the fate of multipotent *PAX6*-expressing radial glial cells (RGCs) (Beattie and Hippenmeyer, 2017; Hansen et al., 2010; Pollen et al., 2015). RGCs are the major neural stem cells (NSCs) that give rise to glia and neurons in the human cortex (Molnár et al., 2019; Silbereis et al., 2016). The fate of these NSCs relies on their ability to undergo symmetric or asymmetric division, a process that ultimately determines whether they self-renew or differentiate into specialized cell types. NSCs give rise to intermediate progenitor cells (IPCs) (Haubensak et al., 2004), which are *EOMES* (TBR2)-expressing (Englund et al., 2005; Kawaguchi et al., 2008) cortex-specific transit-amplifying cells committed to producing glutamatergic projection neurons of cortical layers two to six (Hevner, 2019; Kowalczyk et al., 2009). Given the high bioenergetic demands and metabolic complexity of NSCs, disruption of mitochondrial morphology and function can impair critical stem cell transitions during neurogenesis (Baum et al., 2026; Copp et al., 2013; Iwata et al., 2020). Despite this, the precise role of mitochondrial morphology and function in these intricate transition states is not fully understood.

Myeloid cell leukemia 1 (MCL-1) is a mitochondrial membrane protein identified as a regulator of mitochondrial morphology through mechanisms that are independent of its canonical anti-apoptotic function (Brinkmann et al., 2025; Perciavalle et al., 2012; Rasmussen et al., 2018, 2020; Wang et al., 2013). Initially recognized only as a member of the B cell lymphoma 2 (BCL-2) family for its BCL-2 homology-3 (BH3) domain and role in preventing cell death at the outer mitochondrial membrane (OMM) (Kozopas et al., 1993), MCL-1 has been well established as having non-apoptotic functions. Disruption of MCL-1 causes mitochondrial dysfunction in human cardiomyocytes (Rasmussen et al., 2020; Wang et al., 2013), as well as improper development of hematopoietic stem cells (Opferman et al., 2005), B cell and T cell progenitors (Opferman et al., 2003), activated B cells (Vikstrom et al., 2010), and the central nervous system (Arbour et al., 2008). Inhibition of MCL-1 has been explored as a promising treatment for chemoresistant tumors but has not advanced beyond Phase I/II in clinical trials due to potential cardiotoxicity. Inhibition of MCL-1 with existing small-molecule inhibitors impairs cardiomyocyte function and disrupts mitochondrial morphology, but further investigation is needed to determine whether this also applies to other bioenergetically demanding cell types, such as NSCs.

Previous work has reported a close association between MCL-1, mitochondrial morphology and pluripotent stem cell identity of human embryonic stem cells (hESCs) (Rasmussen et al., 2018). MCL-1 knockdown (*MCL-1^KD^*) in hESCs results in an elongated mitochondrial network, which is a phenotype linked to differentiation. Moreover, *MCL-1^KD^* hESCs exhibit loss of key pluripotent transcription factors and identity markers, including *OCT4* and *NANOG* (Rasmussen et al., 2018). Other studies have established a postmitotic period of fate plasticity, controlled by mitochondrial morphology, during mouse brain development (Iwata et al., 2020). When mitochondria remain elongated (low fission), NSCs divide symmetrically, thus expanding the pool of progenitor cells. Conversely, increased mitochondrial fission drives NSCs toward differentiation (Iwata et al., 2020). *In vivo* mouse studies highlight the importance of MCL-1 in mouse embryonic survival and proper fetal neurodevelopment (Brinkmann et al., 2025). A gene swap approach in which the coding sequence of the *Mcl-1* gene is replaced with other anti-apoptotic genes – *Bcl-2* (*Mcl-1^Bcl-2^*), *Bcl-xL* (*Mcl-1^Bcl-xL^*; *Bcl-xL* is also known as *Bcl2l1*) or *A1* (*Mcl-1^A1^*; *A1* is also known as *Bcl2a1*) – has been reported to result in neurodevelopmental defects that cannot be rescued by anti-apoptotic functions alone, providing evidence that the non-canonical functions of MCL-1 are indispensable for proper neurodevelopment (Brinkmann et al., 2025).

MCL-1 has also been found to localize to the inner mitochondrial membrane (IMM), where it may interact with cristae-shaping proteins, such as optic atrophy type 1 (OPA1) (Perciavalle and Opferman, 2013; Rasmussen et al., 2018, 2020). However, the function of MCL-1 at the IMM remains poorly delineated. Studies in the field have shown that disruption of the fatty acid oxidation (FAO) pathway promotes asymmetric differentiation of adult mouse NSCs, highlighting the role of FAO in regulating NSC fate decisions (Khoa et al., 2020; Knobloch et al., 2017; Ramosaj et al., 2021; Stoll et al., 2015; Xie et al., 2016). Related work in cancer cells from the Opferman and Walensky groups has revealed an unexpected link between MCL-1 and FAO (Escudero et al., 2018; Prew et al., 2022; Wright et al., 2024). Inhibition of MCL-1 results in decreased oxidation of long-chain fatty acids (LCFAs) in cancer cells, likely due to the disrupted interaction between MCL-1 and the BH3-like domain of acyl-CoA synthetase long-chain family member 1 (ACSL1). This suggests that MCL-1 directly modulates FAO in cancer cells.

FAO is a metabolic process critical for energy production and cellular homeostasis that occurs within the mitochondria (Petrelli et al., 2026). This pathway requires LCFAs to translocate from the cytosol and pass through the OMM and IMM to reach their destination for β-oxidation in the mitochondrial matrix. Starting at the OMM, an LCFA is first activated to acyl-CoA derivatives by ACSL1 and then catalyzed by carnitine palmitoyltransferase I (CPT1) into fatty acylcarnitine, which diffuses through the OMM and enters the intermembrane space. The fatty acylcarnitine is then shuttled through the IMM by carnitine-acylcarnitine translocase (CACT, also known as SLC25A20). Once in the matrix, carnitine palmitoyltransferase II (CPT2) cleaves the fatty acylcarnitine, catalyzing the LCFA back to fatty acyl-CoA for β-oxidation in the matrix, initiated by very long acyl-CoA dehydrogenase (VLCAD, also known as ACADVL). The enzymatic breakdown of LCFAs is a multistep process that produces acetyl-CoA, a metabolic byproduct that enters the tricarboxylic acid (TCA) cycle. Gene transcription is influenced by mitochondrial metabolism through several metabolic byproducts, such as acetyl-CoA, α-ketoglutarate and succinate (Arnold et al., 2022; Schvartzman et al., 2018). Disruption of the FAO pathway promotes asymmetric differentiation of pluripotent and neural stem cells (Khoa et al., 2020; Knobloch et al., 2017; Ramosaj et al., 2021; Stoll et al., 2015; Xie et al., 2016), suggesting a role of FAO in stem cell fate decisions. Although FAO modulates neurodevelopment and MCL-1 is known to impact FAO in cancer cells, the role of MCL-1 in FAO during human neurodevelopment has not been explored. Here, we report the non-apoptotic effects of MCL-1 inhibition on mitochondrial morphology, cristae architecture and FAO in an *in vitro* model of early human neurogenesis. Our findings provide evidence that MCL-1 maintains the integrity of mitochondrial cristae and neural progenitor cell identity. While several of the individual phenotypes we describe, including cristae disruption, lipid droplet accumulation and impaired FAO, have precedent in other systems, their coordinated occurrence in a physiologically relevant, primary human neurodevelopmental model, together with a direct link to progenitor identity, has not previously been demonstrated and provides a bridge between mechanistic studies in simplified systems and the developmental relevance of MCL-1 in human neurogenesis.

## RESULTS

### MCL-1 inhibition induces mitochondrial remodeling and inner membrane reorganization in human neural progenitor cells

MCL-1 is known for its canonical function as an anti-apoptotic member of the BCL-2 family and is indispensable for the survival of human induced pluripotent stem cells (hiPSCs) (Rasmussen et al., 2018), hematopoietic stem cells (Opferman and Kothari, 2018) and cardiomyocytes (Rasmussen et al., 2020). To examine the effect of MCL-1 inhibition on human neural progenitor cells (NPCs), we differentiated hESCs using dual SMAD inhibition, as previously described (Chambers et al., 2009). Human NPCs were treated with S63845, a selective BH3 mimetic inhibitor of MCL-1, for 24 or 48 h (Fig. 1A). Cells were co-treated with Q-VD-OPh (QVD), a potent pan-caspase inhibitor, to distinguish between phenotypes that are attributed to the anti-apoptotic or non-apoptotic functions of MCL-1, and to capture the transient state of cell types that are more vulnerable to MCL-1 inhibition. Using real-time quantitative polymerase chain reaction (RT-qPCR), we detected downregulation of *MCL-1* with S63485 treatment at 24 h but not at 48 h, whereas co-treatment with QVD upregulated *MCL-1* expression at both timepoints (Fig. S1A). Previous studies have reported an increase in MCL-1 protein levels upon MCL-1 inhibition, which is speculated to result from stabilization of MCL-1, whereby it is protected from proteasomal degradation when its BH3 domain is occupied (Kotschy et al., 2016; Varadarajan et al., 2013). We probed for MCL-1 protein levels and confirmed an increase at both timepoints with S63845 treatment (Fig. S1B,C) despite the downregulation of *MCL-1* expression at 24 h. This increase in MCL-1 protein levels serves as an internal control that the S63845 small molecule is effectively inhibiting MCL-1 in NPCs. Increased MCL-1 levels were not detected with QVD co-treatment despite upregulation of *MCL-1* with co-treatment at both timepoints (Fig. S1A–C), confirming that QVD co-treatment captures the state of a subset of NPCs that are more vulnerable to MCL-1 inhibition.

**Fig. 1.**
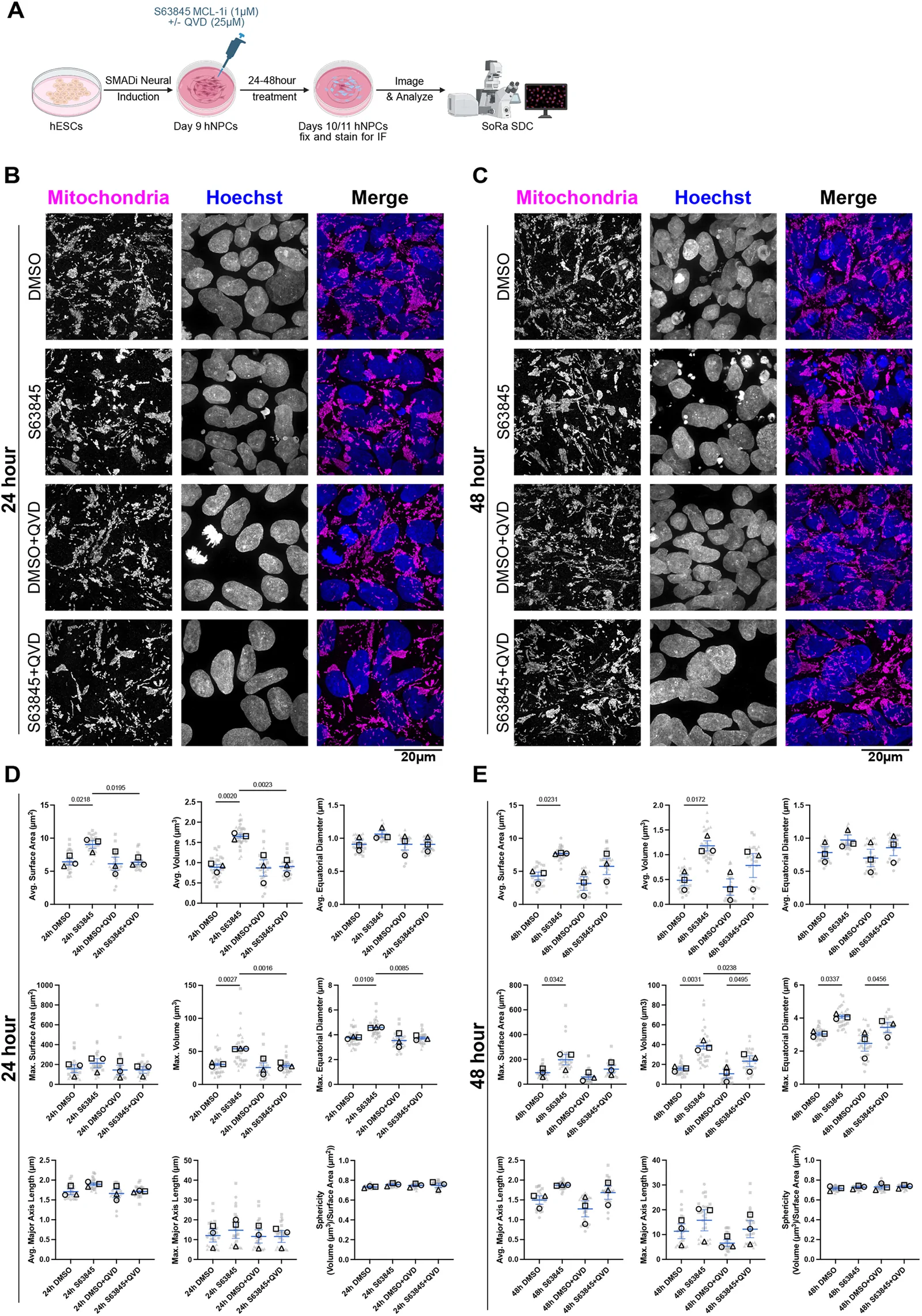
**S63845 treatment impacts mitochondrial content and morphology.** (A) Schematic detailing the neural induction of hESCs using SMAD inhibition (SMADi) for the derivation of the human NPC (hNPC) tissue culture model. NPCs were maintained in neural induction medium (NIM) with daily medium changes until they were passaged on day 7. NPCs were given a 24 h acclimation period on day 8 before treatment on day 9 with 1 μM S63845 (MCL-1 inhibitor) with or without 25 μM QVD (caspase inhibitor). Cells were then fixed in paraformaldehyde at 24 h and 48 h post-treatment on days 10 and 11, respectively, and processed for immunofluorescence (IF) imaging on a SoRa spinning-disk confocal (SDC) microscope. (B,C) Immunofluorescence images of mitochondria (anti-mitochondria, magenta) and nuclei (Hoechst, blue) acquired on a SoRa spinning-disk confocal microscope at 100× with 2.8× magnification. Representative images (*n*=3) of NPCs treated with S63845 (MCL-1 inhibitor) with or without QVD (caspase inhibitor) for 24 h (B) and 48 h (C). (D,E) Quantified mitochondrial morphology parameters including sphericity, average (avg.) and maximum (max.) surface area, volume, equatorial diameter and major axis length for the 24 h (D) and 48 h (E) treatments. Each large, open data point (black) represents the mean of a biological replicate, and each small, closed data point (transparent gray) represents a technical replicate (a single image). Shape of the data points corresponds to a biological replicate (circle for replicate 1, square for replicate 2 and triangle for replicate 3). Data were analyzed using an ordinary two-way ANOVA with an uncorrected Fisher's LSD test for multiple comparisons and a 95% confidence level (*P*≤0.05 as the significance threshold; *P*-values are indicated in the figure). Error bars represent s.e.m.

MCL-1 inhibition is known to alter mitochondrial morphology in various cell types. Thus, we first investigated the effects of S63845 on mitochondrial morphology in human NPCs. Mitochondrial morphology was quantified as previously described (Rasmussen et al., 2020; Robertson et al., 2023). Super-resolution optical pixel reassignment (SoRa) microscopy revealed a pronounced swelling of the mitochondrial network in NPCs treated with S63845 at both timepoints (Fig. 1B,C). Cell viability was not overtly compromised by S63845 treatment, as evidenced by comparable nuclear morphology across all conditions and timepoints examined (Fig. 1B,C). Consistent with these qualitative observations, average mitochondrial surface area and volume were significantly increased at both timepoints (Fig. 1D,E). Moreover, maximum mitochondrial volume and equatorial diameter were significantly increased at both timepoints, indicating a globally expanded mitochondrial network. At 48 h, this expansion was further reflected by a significant increase in maximum surface area in S63845-treated cells relative to controls (Fig. 1E). Notably, this mitochondrial network expansion, rather than the fragmentation typically associated with apoptotic cell death, is consistent with a non-apoptotic cellular response to MCL-1 inhibition. To evaluate the contribution of caspase activity to these morphological changes, cells were co-treated with QVD. At 24 h, co-treatment with QVD significantly abrogated the mitochondrial network expansion induced by S63845, with no significant differences observed relative to QVD-only controls (Fig. 1D). In contrast, at 48 h, the S63845-induced mitochondrial remodeling was maintained despite caspase inhibition, suggesting that the dependency on caspase activity for mitochondrial network expansion is temporally restricted to earlier stages of the cellular response (Fig. 1E).

Given the critical role of mitochondrial cristae in metabolic regulation, we next investigated whether MCL-1 inhibition-induced alterations in mitochondrial morphology at 48 h of S63845 treatment extended to cristae ultrastructure using transmission electron microscopy (TEM) and focused ion beam–scanning electron microscopy (FIB–SEM) (Boassa et al., 2022; Garza-Lopez et al., 2021; Tsang et al., 2018) (Fig. 2A). TEM images revealed atypical cristae morphology in NPCs treated with S63845 for 48 h, characterized by swollen matrices and sparse, disorganized cristae membranes, in contrast to the densely folded inner membrane architecture observed in control mitochondria (Fig. 2B). To further characterize these ultrastructural changes in three dimensions, we performed volumetric reconstruction of mitochondrial cristae with FIB–SEM-acquired images (Fig. 2C–E). Three-dimensional analyses revealed significant increases in cristae surface area and volume in S63845-treated cells relative to controls (Fig. 2D). Volumetric reconstruction of mitochondria (Fig. S2A) revealed that neither the mitochondrial branching index (MBI) nor sphericity were significantly affected (Fig. S2B). Collectively, these findings demonstrate that MCL-1 inhibition induces extensive disruption of cristae organization (Fig. 2A–E; Movies 1 and 2). These data implicate MCL-1 as a critical regulator of the structural integrity of the IMM.

**Fig. 2.**
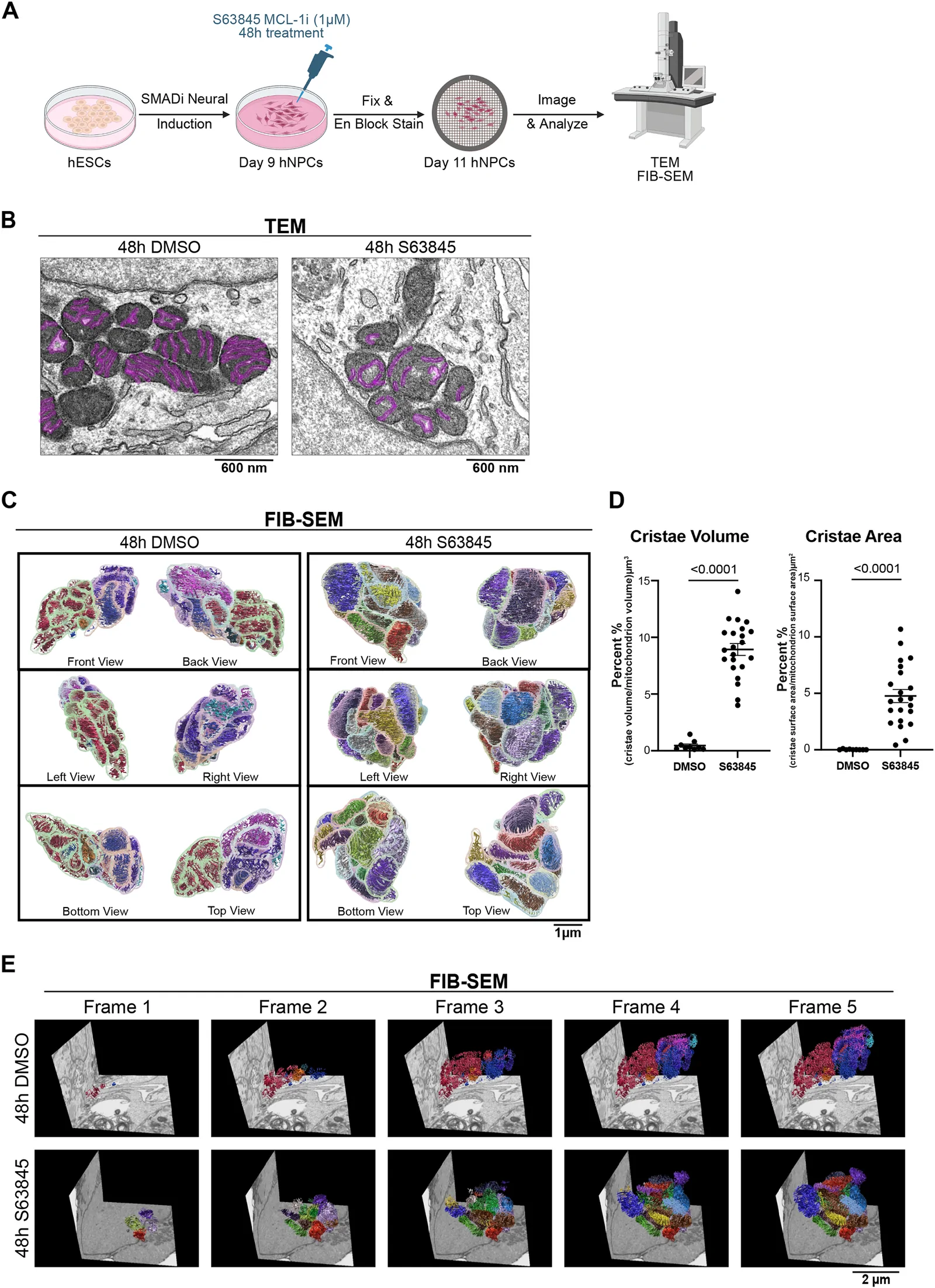
**S63845 treatment compromises mitochondrial cristae integrity.** (A) Schematic detailing the neural induction of hESCs using SMAD inhibition (SMADi) for the derivation of the human NPC (hNPC) tissue culture model. NPCs were maintained in neural induction medium (NIM) with daily medium changes until they were passaged on day 7. NPCs were given a 24 h acclimation period on day 8 before treatment on day 9 with 1 μM S63845 (MCL-1 inhibitor). Cells were then collected at 48 h post treatment on day 11 and processed for TEM or FIB–SEM. (B) Representative TEM images (*n*=3) of NPC mitochondrial cristae 48 h post treatment with DMSO control and S63845 MCL-1 inhibitor. Mitochondria traced in magenta. (C) Three-dimensional reconstructions of mitochondria and cristae. (D) Quantification of cristae changes from three-dimensional reconstruction. Each cristae volume and surface area was normalized to its corresponding mitochondrion volume and surface area, respectively, and multiplied by 100 to calculate the percentage of cristae-occupied mitochondrial volume or area. Each data point is an individual mitochondrion; data pooled from *n*=3 independent samples. Error bars represent s.e.m. Data were analyzed using a parametric two-tailed unpaired *t*-test with a Welch's correction (*P*-values are indicated in the figure). (E) Volumetric still frames of mitochondrial cristae three-dimensional reconstruction of NPCs at 48 h post treatment with DMSO control and S63845 (MCL-1 inhibitor) segmented from FIB–SEM volume images. Each color represents the cristae of an individual mitochondrion.

### MCL-1 activity preserves OPA1 proteostasis, MICOS complex stability and mitochondrial respiration

Given the crista defects observed upon MCL-1 inhibition, we next examined key molecular regulators of cristae architecture and junction formation (Fig. 3A). Cristae structure is maintained by the mitochondrial contact site and cristae-organizing system (MICOS), which comprises two core subcomplexes: the MIC60 subcomplex (MIC60, MIC19 and MIC25, also known as IMMT, CHCHD3 and CHCHD6, respectively), and the MIC10 subcomplex (MIC10, MIC26 and MIC27, also known as MICOS10, APOO and APOOL, respectively), bridged by MIC13 (also known as MICOS13 or QIL1) (Eramo et al., 2020). At the IMM, MICOS establishes cristae junctions, which are required for cristae biogenesis and expansion of IMM surface area to support ATP synthesis via oxidative phosphorylation (OXPHOS) through the electron transport chain (ETC) (Colina-Tenorio et al., 2020, 2026; Stephan et al., 2020). The large GTPase OPA1 further contributes to cristae architecture by stabilizing MICOS complex integrity (Glytsou et al., 2016). Specifically, the long isoform of OPA1 (OPA1-L) mediates IMM fusion (Hu et al., 2020) and maintains cristae lumen width, whereas the short isoforms (OPA1-S1 and OPA1-S2, collectively OPA1-S1/2) cooperatively regulate cristae lumen length (von der Malsburg et al., 2023).

**Fig. 3.**
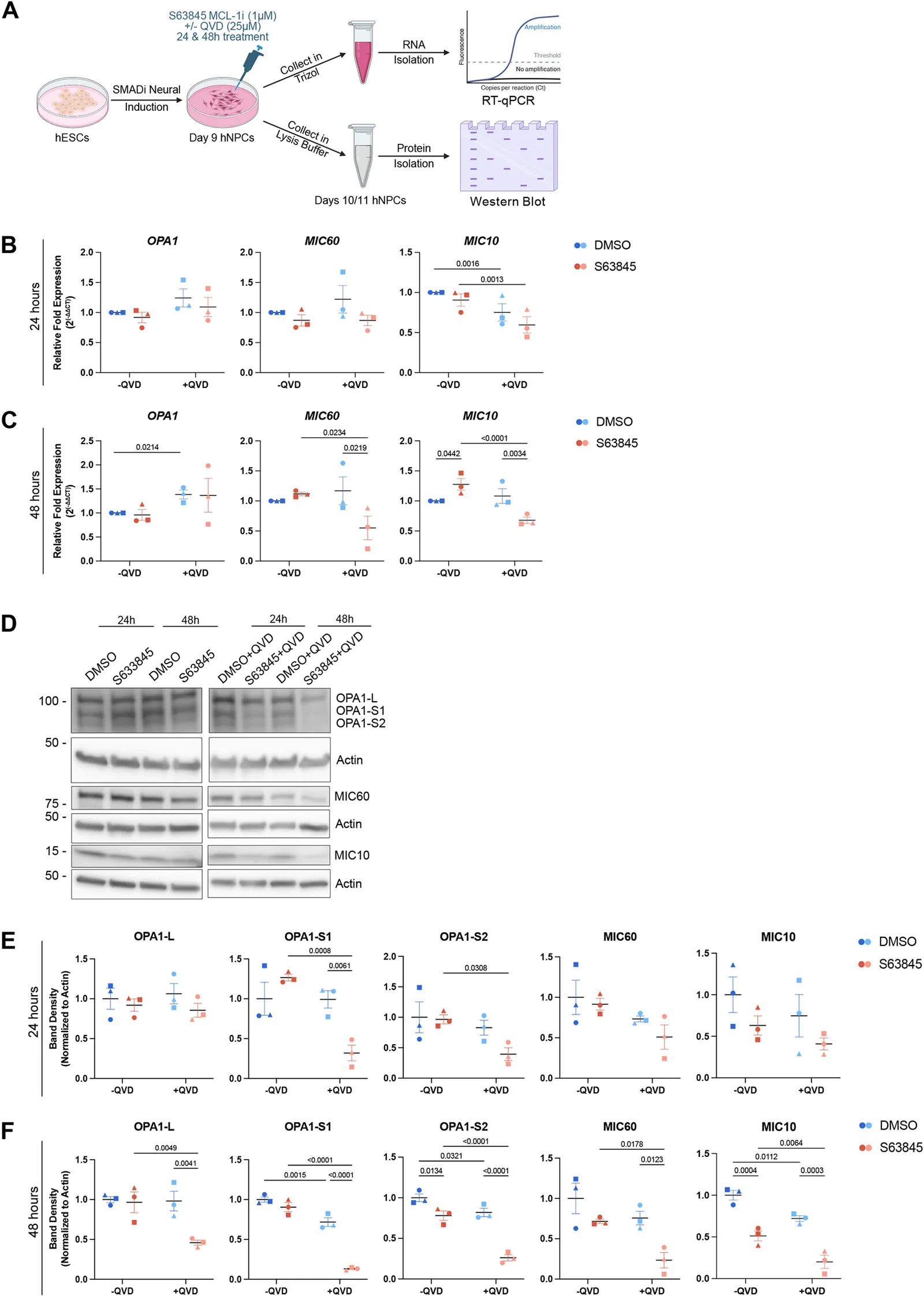
**S63845 and QVD co-treatment reveal altered levels of cristae-shaping proteins.** (A) Schematic detailing the neural induction of hESCs using SMAD inhibition (SMADi) for the derivation of the human NPC (hNPC) tissue culture model. NPCs were maintained in neural induction medium (NIM) with daily medium changes until they were passaged on day 7. NPCs were given a 24 h acclimation period on day 8 before treatment on day 9 with 1 μM S63845 (MCL-1 inhibitor) with or without 25 μM QVD (caspase inhibitor). Cells were then collected at 24 h and 48 h post treatment on days 10 and 11, respectively, in either Trizol for RNA extraction and RT-qPCR, or protease inhibitor lysis buffer for protein isolation and western blotting. (B,C) Quantified relative expression of *OPA1*, *MIC60* and *MIC10* after 24 h (B) and 48 h (C) treatments. Each data point represents a biological replicate (*n*=3). Shape of the data points corresponds to a biological replicate (circle for replicate 1, square for replicate 2 and triangle for replicate 3). Each data point is the average of two technical replicates for all RT-qPCR data. RT-qPCR data are displayed as relative fold expression derived from 2^(−ΔΔ*Ct*)^, and raw data were analyzed using ANCOVA as described in the Materials and Methods. (D) Representative western blot images (*n*=3) of OPA1-L, OPA1-S1, OPA1-S2, MIC60 and MIC10 after 24 h and 48 h treatments. Note that the actin loading control blots for MIC60 and MIC10 (no QVD) are shown again in Fig. S3A as the loading control blots for MIC25/MIC13 and MIC19 (no QVD), respectively. The actin loading control for MIC10 (+QVD) is shown again in Fig. S3A as the loading control for MIC19 (+QVD). Molecular masses are indicated in kDa. (E) Quantified western blot band density normalized to actin loading control derived from western blots of OPA1-L, OPA1-S1, OPA1-S2, MIC60 and MIC10 after 24 h (E) and 48 h (F) treatments. Each data point represents a biological replicate (*n*=3). Shape of the data points corresponds to a biological replicate (circle for replicate 1, square for replicate 2, and triangle for replicate 3). Band density data were analyzed using an ordinary two-way ANOVA with an uncorrected Fisher's LSD test for multiple comparisons. All tests used a 95% confidence level (*P*≤0.05) as the significance threshold. *P*-values are indicated in the figure. Error bars represent s.e.m.

To determine whether MCL-1 inhibition affects the transcriptional regulation of these structural components, mRNA expression of *OPA1*, *MIC60* and *MIC10* was assessed at 24 and 48 h following S63845 treatment (Fig. 3B,C). At 24 h, MCL-1 inhibition did not significantly alter the expression of *OPA1*, *MIC60* or *MIC10* (Fig. 3B). At 48 h, however, *MIC10* mRNA levels were significantly increased by MCL-1 inhibition, whereas *MIC60* and *OPA1* expression remained unchanged (Fig. 3C). Co-treatment with the pan-caspase inhibitor QVD captured a reduction in the expression of both *MIC60* and *MIC10* at 48 h, suggesting a caspase-dependent component to the transcriptional regulation of MICOS subunits under conditions of MCL-1 inhibition.

To determine whether the transcriptional changes observed at the mRNA level were reflected at the protein level, we next performed immunoblotting for OPA1 isoforms, MIC60 and MIC10 across all treatment conditions and timepoints (Fig. 3D). At 24 h, MCL-1 inhibition alone did not significantly alter OPA1 isoform levels; however, combined MCL-1 and caspase inhibition induced a significant reduction in OPA1-S1/2 protein levels (Fig. 3E). At 48 h, a reduction in OPA1-S1 and OPA1-S2 was observed across multiple conditions, with the most pronounced decrease occurring under combined S63845 and QVD co-treatment, and OPA1-L levels were also significantly reduced at this timepoint (Fig. 3F). These isoform-specific reductions suggest that MCL-1 inhibition disrupts the stoichiometric balance between fusion-competent long-form OPA1 (OPA1-L) and the OPA1 short isoforms (OPA1-S1/2), potentially reflecting an impairment of OPA1-L proteolytic processing rather than enhanced cleavage activity.

Assessment of MICOS core subunit abundance revealed no significant changes in MIC60 or MIC10 protein levels at 24 h under any condition (Fig. 3E). However, MIC13, which serves as a bridge between MIC60 and MIC10 subcomplexes, was significantly reduced in abundance at 24 h (Fig. S3A,B). At 48 h, MCL-1 inhibition alone induced a significant reduction in MIC10 protein levels, despite transcriptional upregulation observed at this timepoint (Fig. 3C), whereas MIC60 levels remained unchanged (Fig. 3F). MIC25 and MIC13 were also destabilized following 48 h of S63845 treatment (Fig. S3C). At 48 h, co-treatment with QVD further revealed a reduction in MIC60, MIC10, MIC19, MIC25 and MIC13 protein levels at 48 h (Fig. 3F; Fig. S3C), indicating that caspase inhibition unmasks a broader destabilization of the MICOS complex. Collectively, these findings identify MCL-1 as a critical upstream regulator of IMM organization, acting through parallel destabilization of OPA1 isoform homeostasis and MICOS complex integrity to maintain cristae architecture.

To assess the consequences of these mitochondrial changes on cellular respiration, we performed respirometry assays using an Agilent Seahorse XF Mito Stress Test to measure oxygen consumption rate (OCR). At 24 h of MCL-1 inhibition, there was a significant decrease in maximal respiration, spare respiratory capacity and the percentage of spare respiratory capacity, which were not rescued by QVD treatment (Fig. S4A,B). The failure of QVD to rescue any of the observed OCR deficits confirms that the respiratory impairment is not secondary to caspase activation or apoptotic mitochondrial permeabilization. Co-treatment with QVD further revealed decreased coupling efficiency and non-mitochondrial OCR (Fig. S4A,B). At 48 h of MCL-1 inhibition, the percentage of spare respiratory capacity remained decreased and was not rescued by QVD. Co-treatment with QVD at 48 h revealed a decrease in basal respiration, maximal respiration, ATP-linked production, non-mitochondrial OCR and spare respiratory capacity (Fig. S4C,D). Thus, cristae changes induced by MCL-1 inhibition cause a progressive, caspase-independent impairment of mitochondrial respiration, characterized by deficits in maximal respiration and spare respiratory capacity at 24 h that worsen by 48 h to include reductions in basal respiration and ATP-linked production.

### MCL-1 inhibition impairs FAO

We predicted that FAO transmembrane enzymes and transport proteins rely heavily on the correct assembly and structure of the OMM and IMM for proper activity (Fig. 4A). Previous investigations have shown close interactions between MCL-1 and FAO enzymes in cancer cells, and mouse studies have also established the importance of FAO for the maintenance of embryonic neurodevelopment and adult NSCs (Brinkmann et al., 2025; Prew et al., 2022). To explore whether the mitochondrial structural perturbations observed upon MCL-1 inhibition extend to mitochondrial metabolic function, we assessed key regulators of fatty acid β-oxidation (Fig. 4B). At 24 h, protein levels of the CPT1a isoform of CPT1 were increased following MCL-1 inhibition, whereas ACSL1, CPT2 and VLCAD levels were not significantly affected (Fig. 4C). Co-treatment with QVD at 24 h revealed a significant decrease in CPT1a, CPT2 and VLCAD levels compared to those in DMSO-treated cells, with ACSL1 also showing a reduction, albeit not significant compared to the vehicle control, but significantly reduced when compared to MCL-1 inhibition alone (Fig. 4C). At 48 h of MCL-1 inhibition alone, CPT1a protein levels remained elevated along with VLCAD, whereas CPT2 levels significantly decreased (Fig. 4D). Co-treatment with QVD at 48 h resulted in higher levels of CPT1a and a significant decrease in CPT2 and VLCAD levels compared to those in the control (Fig. 4D).

**Fig. 4.**
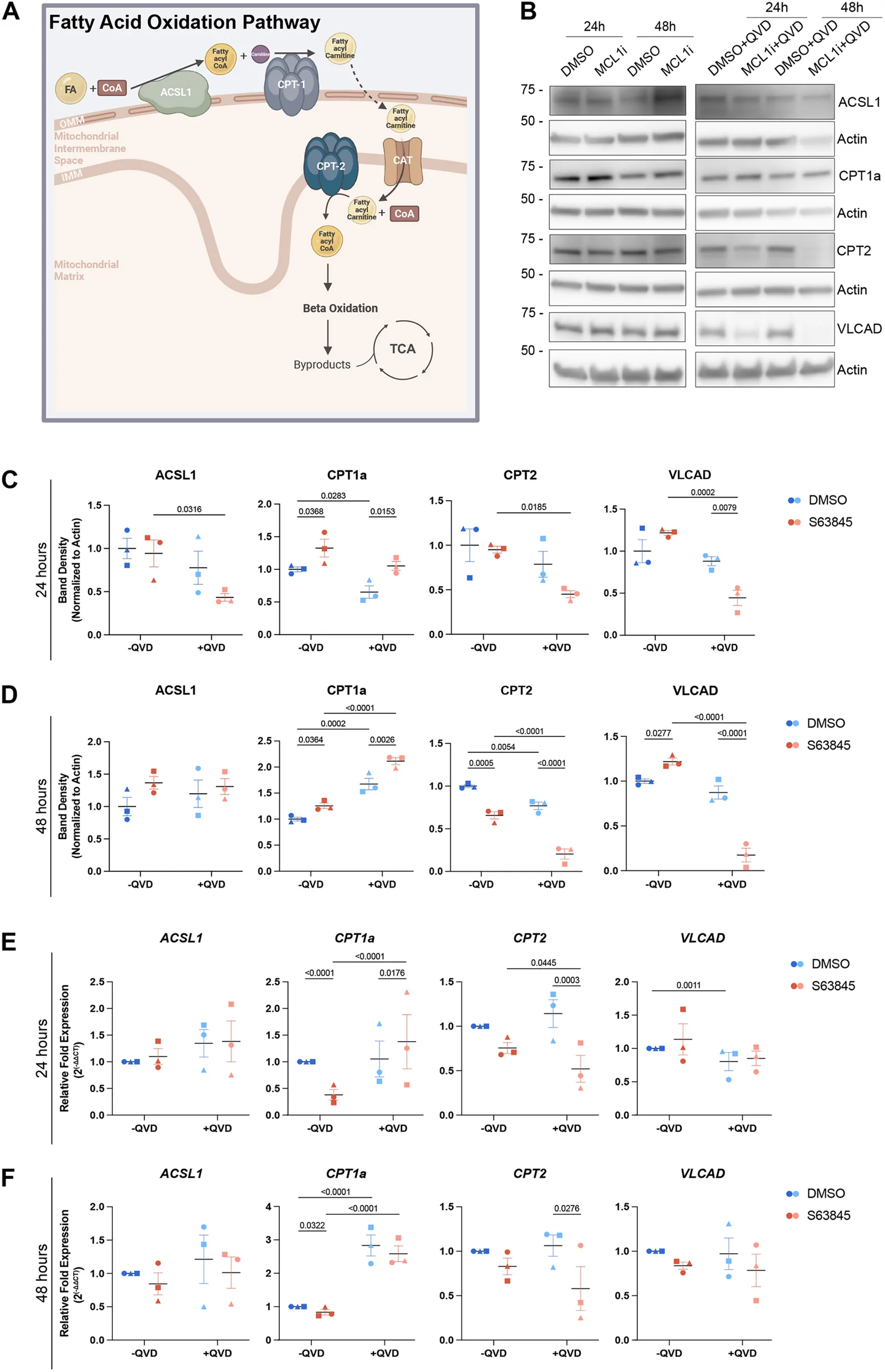
**S63845 and QVD co-treatment results in downregulation of carnitine palmitoyltransferase and altered levels of FAO enzymes.** (A) Schematic of the mitochondrial fatty acid β-oxidation pathway. CAT, carnitine-acylcarnitine translocase; FA, fatty acid. (B) Representative western blot images (*n*=3). Note that the actin control blots for VLCAD are shown again in Fig. S1B as the loading controls for MCL-1. MCL1i, MCL-1 inhibition with S63846. Molecular masses are indicated in kDa. (C,D) Quantified western blot band density normalized to actin loading control of FAO enzymes ACSL1, CPT1a, CPT2 and VLCAD after S63845 (MCL-1 inhibitor) treatment with or without QVD (caspase inhibitor) treatment for 24 h (C) and 48 h (D). Band density data were analyzed using an ordinary two-way ANOVA with an uncorrected Fisher's LSD test for multiple comparisons. *n*=3. (E,F) Quantified relative expression of FAO enzymes *ACSL1*, *CPT1a*, *CPT2* and *VLCAD* after 24 h (E) and 48 h (F) of the indicated treatments (*n*=3). Two technical replicates were used for all RT-qPCR data. RT-qPCR data are displayed as relative fold expression derived from 2^(−ΔΔ*Ct*)^, and raw data were analyzed using ANCOVA as described in the Materials and Methods. In C–F, each data point on the graph represents a biological replicate. Shape of data point corresponds to each biological replicate (circle for replicate 1, square for replicate 2 and triangle for replicate 3). All tests used a 95% confidence level (*P*≤0.05) as the significance threshold. *P*-values are indicated in the figure. Error bars represent s.e.m.

Previous studies have reported a global downregulation of the FAO pathway at the mRNA level upon MCL-1 deletion in B cell acute lymphoblastic leukemia (B-ALL cells) (Prew et al., 2022). We performed RT-qPCR-based mRNA expression analyses to examine the expression patterns of FAO genes in NPCs treated with S63845. At 24 and 48 h of MCL-1 inhibition alone, *ACSL1*, *CPT2* and *VLCAD* levels remained unchanged, whereas *CPT1a* was significantly downregulated (Fig. 4E,F) despite higher protein levels detected at both timepoints (Fig. 4C,D). This suggests that CPT1a protein accumulation occurs through a post-transcriptional mechanism that stabilizes the protein under combined MCL-1 and caspase inhibition rather than transcriptional upregulation. This relationship between *CPT1a* mRNA and protein persisted in the presence of QVD at 48 h (Fig. 4F). At 24 h, however, inhibition of MCL-1 produced a significant upregulation of *CPT1a* mRNA, albeit with high variability (Fig. 4E). QVD co-treatment resulted in upregulation of *CPT2* at both timepoints (Fig. 4E,F). Notably, this transcriptional decrease in *CPT2* was accompanied by a corresponding reduction in CPT2 protein (Fig. 4B–D), suggesting that caspase inhibition engages an additional transcriptional suppression of CPT2 beyond the post-transcriptional effects seen with MCL-1 inhibition alone. Consistent with the transcriptional suppression previously reported (Prew et al., 2022), we observe downregulation of *CPT1a* mRNA and protein with MCL-1 inhibition alone, while other genes encoding for FAO enzymes, including ACSL1, CPT2 and VLCAD, show no transcriptional change despite alterations in protein level. This discordance between mRNA and protein levels in our model points to post-translational mechanisms as the primary drivers of FAO enzyme loss, rather than the transcriptional downregulation previously reported upon MCL-1 deletion in cancer cells.

We next sought to determine whether enzyme dysregulation impairs FAO by directly assessing cellular fatty acid storage. Fatty acids are stored in lipid droplet organelles when they are not processed by β-oxidation. Accumulation of lipid droplets upon MCL-1 inhibition has previously been reported in cancer cells and murine liver as a marker of disrupted fatty acid β-oxidation (Wright et al., 2024). To further investigate the impact of MCL-1 inhibition on FAO, NPCs were cultured in nutrient-deficient DMEM medium supplemented only with palmitate during treatment with the MCL-1 inhibitor for 12 h, followed by a 2 h starvation period in HBSS under continuous MCL-1 inhibition with S63845 (Fig. 5A). Consistent with altered FAO enzyme levels detected by RT-qPCR and immunoblotting, and with previous reports (Wright et al., 2024), S63845-treated NPCs accumulated excess neutral lipids when challenged with palmitate as their only resource for fuel (Fig. 5B). Quantitative image analysis revealed a significant increase in average lipid droplet volume and a trending increase in maximum volume (Fig. 5C), indicative of reduced lipid turnover and a shift toward storage. These data suggest that MCL-1 sustains metabolic flux as fatty acids fail to import to the matrix and accumulate in lipid droplets.

**Fig. 5.**
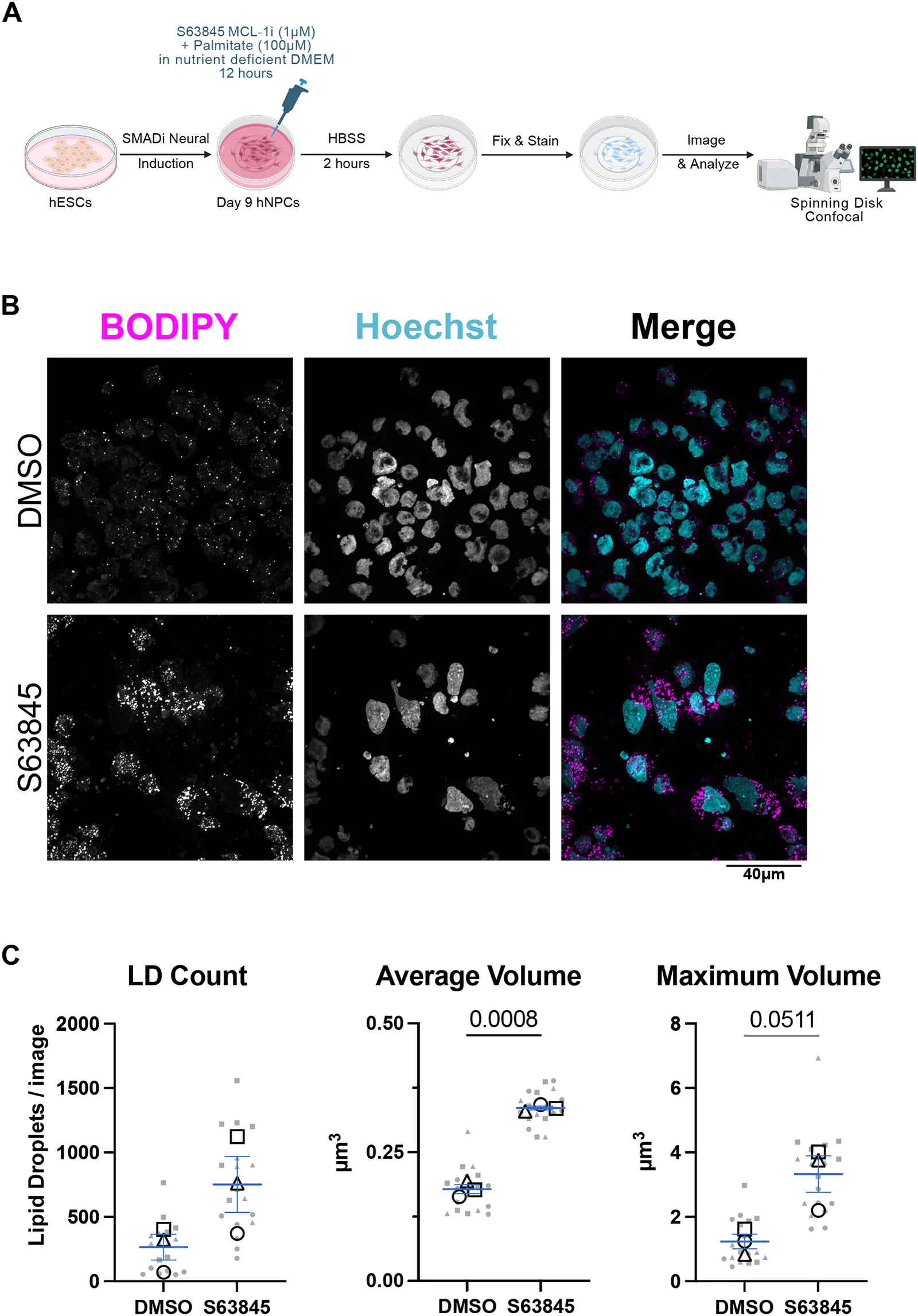
**NPCs supplemented with palmitate and treated with S63845 accumulate lipid droplets*.*** (A) Schematic detailing the neural induction of hESCs using SMAD inhibition (SMADi) for the derivation of the human NPC (hNPC) tissue culture model. NPCs were maintained in neural induction medium (NIM) with daily medium changes until they were passaged on day 7. NPCs were given a 24 h acclimation period on day 8 before treatment on day 9 with 1 μM S63845 (MCL-1 inhibitor, MCL-1i) in nutrient-deficient DMEM medium (no glucose, no glutamine, no pyruvate) and supplemented with 100 μM palmitate for 12 h followed by a 2 h starvation in HBSS. Cells were then live-stained with BODIPY and Hoechst prior to paraformaldehyde fixation for imaging on a spinning-disk confocal microscope. (B) Representative immunofluorescence images (*n*=3) of lipid droplets (BODIPY, magenta) and nuclei (Hoechst, cyan) were acquired on a spinning-disk confocal microscope at 100×. (C) Quantification of lipid droplets per image, average lipid droplet volume and maximum lipid droplet volume. Each large, open data point (black) represents the mean of a biological replicate, and each small, closed data point (transparent gray) represents a technical replicate (a single image). Shape of the data points corresponds to a biological replicate (*n*=3; circle for replicate 1, square for replicate 2 and triangle for replicate 3). Data were analyzed using an ordinary two-way ANOVA with an uncorrected Fisher's LSD test for multiple comparisons and a 95% confidence level (*P*≤0.05) as the significance threshold. *P*-values are indicated in the figure. Error bars represent s.e.m.

### MCL-1 inhibition delocalizes ACLS1 from the OMM

Previous studies in cancer cells have identified an ACSL1 BH3-like binding domain that mediates a direct interaction between ACSL1 and MCL-1 at the OMM (Escudero et al., 2018; Wright et al., 2024). Given that ACSL1 catalyzes the first committed step of the FAO cascade, its proper localization to the OMM is essential for efficient fatty acid activation and subsequent mitochondrial import. To investigate whether MCL-1 serves as a scaffold for ACSL1 recruitment or retention at the OMM, we generated a stable hESC line overexpressing ACSL1–HaloTag and differentiated these cells into NPCs for live-cell imaging. ACSL1–HaloTag was detected using a JFX dye, and mitochondria were labeled with MitoTracker Green FM. To achieve super-resolution, cells were live-imaged on a SoRa spinning-disk confocal microscope using a 100× objective with 2.8× magnification (Fig. 6A). Colocalization image analysis revealed a significant decrease in mitochondria-associated ACSL1 under two defining parameters following MCL-1 inhibition at both 24 and 48 h (Fig. 6B), consistent with a role for MCL-1 in the proper retention of ACSL1 at the OMM. Together with the lipid droplet accumulation data, these findings suggest that MCL-1 inhibition disrupts FAO at its earliest enzymatic step by displacing ACSL1 from its site of action, thereby reducing fatty acid activation and mitochondrial import capacity.

**Fig. 6.**
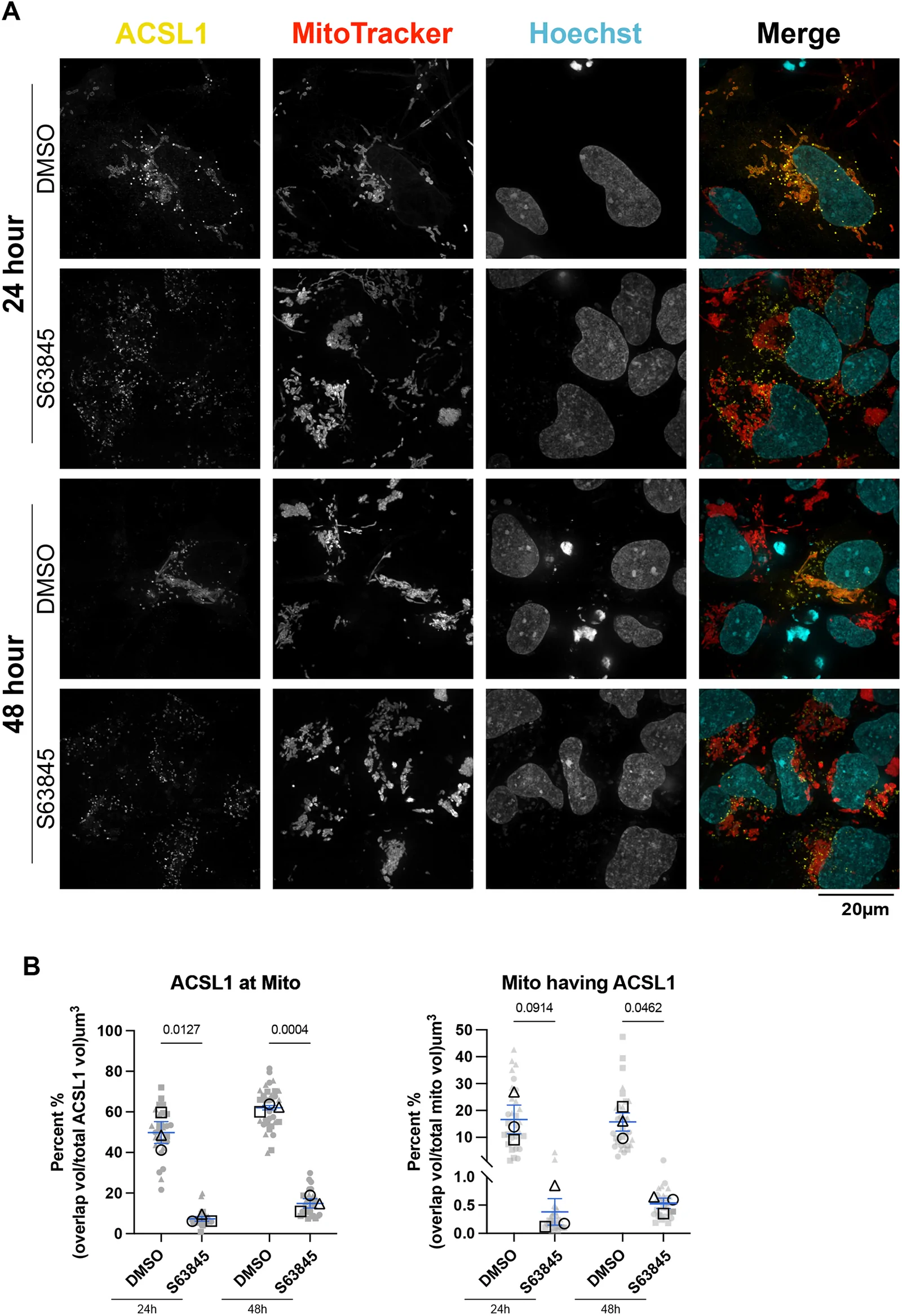
**S63845 treatment displaces ACSL1 from the mitochondria.** (A) Immunofluorescence images of ACSL1–HaloTag (JFX, yellow) mitochondria (MitoTracker Green FM, red) and nuclei (Hoechst, cyan) were acquired live on a SoRa spinning-disk confocal microscope at 100× with 2.8× magnification. Representative images (*n*=3) of NPCs treated with S63845 (MCL-1 inhibitor) or DMSO vehicle control for 24 h (top) and 48 h (bottom). (B) Quantification of total ACSL1 localized at the mitochondria (left) and calculated percentage of mitochondria with ACSL1 instances (right). Each large, open data point (black) represents the mean of a biological replicate, and each small, closed data point (transparent gray) represents a technical replicate (a single image). Shape of the data points corresponds to a biological replicate (*n*=3; circle for replicate 1, square for replicate 2, and triangle for replicate 3). Data were analyzed using an ordinary two-way ANOVA with an uncorrected Fisher's LSD test for multiple comparisons and a 95% confidence level (*P*≤0.05) as the significance threshold. *P*-values are indicated in the figure. Error bars represent s.e.m.

### MCL-1 maintains neural progenitor identity and supports neuronal differentiation

Given the role of MCL-1 in FAO and the mitochondrial and metabolic perturbations triggered by MCL-1 inhibition, we investigated the effects of S63845 treatment on neurogenic progression. All findings were observed on differentiation days 10 and 11 for 24 h and 48 h treatments, respectively. Although we did not detect changes in global protein levels of NPC identity markers (Fig. S5A–C), we observed a significant downregulation of various identity markers (Fig. S5D,E). At 24 h of MCL-1 inhibition alone, *PAX6* (a marker of NSC identity), *EOMES* (the gene encoding TBR2, a marker of IPCs), *TBR1* (a postmitotic marker of newborn glutamatergic projection neurons), and *TUBB3* (a neuronal microtubule marker and early marker for newborn neurons) were downregulated (Fig. S5D). Co-treatment with QVD at 24 h rescued *PAX6* and *EOMES* mRNA levels, but not those of *TBR1* and *TUBB3* (Fig. S5D). At 48 h of MCL-1 inhibition alone, *PAX6*, *EOMES* and *TBR1* remained downregulated, but not *TUBB3* (Fig. S5E). Co-treatment with QVD at 48 h persistently rescued *PAX6* as well as *TBR1* but not *EOMES* and maintained a downregulation in *TUBB3* (Fig. S5E). We followed these analyses with immunofluorescence imaging, which revealed a small population of IPCs (PAX6^−^ TBR2^+^) in the culture of NPCs. IPCs are progenitor cells that are committed to give rise to deep layer glutamatergic projection neurons. MCL-1 inhibition selectively reduced the proportion of IPCs at 24 h of MCL-1 inhibition without rescue by QVD co-treatment (Fig. 7A). Loss of IPCs persisted at 48 h (Fig. 7B). These findings suggest that MCL-1 is critical for maintaining the identity of this progenitor pool through non-apoptotic mechanisms. These imaging-based findings are supported by the RT-qPCR analysis reported above, which shows reduced *PAX6* and *EOMES* transcript levels upon MCL-1 inhibition (Fig. S5D,E), providing independent, marker-based validation of the reduction in progenitor identity observed by immunofluorescence. To determine how these effects on IPCs affect neuronal differentiation, we first examined cell proliferation using EdU labeling and found no significant changes in early or late S-phase or in global proliferation states across all treatment conditions and timepoints (Fig. S6A–D). To further investigate the potency of neural stem/progenitor cells, we examined their ability to differentiate and give rise to newborn neurons using βIII-tubulin (TUBB3) staining (Fig. 8A,B). Control cells formed dense neurite networks with extensive βIII-tubulin labeling, whereas MCL-1-inhibited neurons displayed sparse and truncated processes, reflecting defective neurite extension and maturation at both timepoints (Fig. 8A,B). When treated with the MCL-1 inhibitor, neural stem/progenitor cells give rise to fewer newborn neurons with retracted neuronal processes (Fig. 8C,D); however, most parameters were rescued upon co-treatment with QVD, except for average major axis length and average surface area at 48 h of S63845 treatment. Taken together, these findings shed light on the importance of MCL-1 for the potency of neural stem/progenitor cells to undergo proper differentiation and for newborn neurons to undergo proper maturation. The absence of overt cytotoxicity suggests that MCL-1 directly supports neuronal commitment rather than survival alone.

**Fig. 7.**
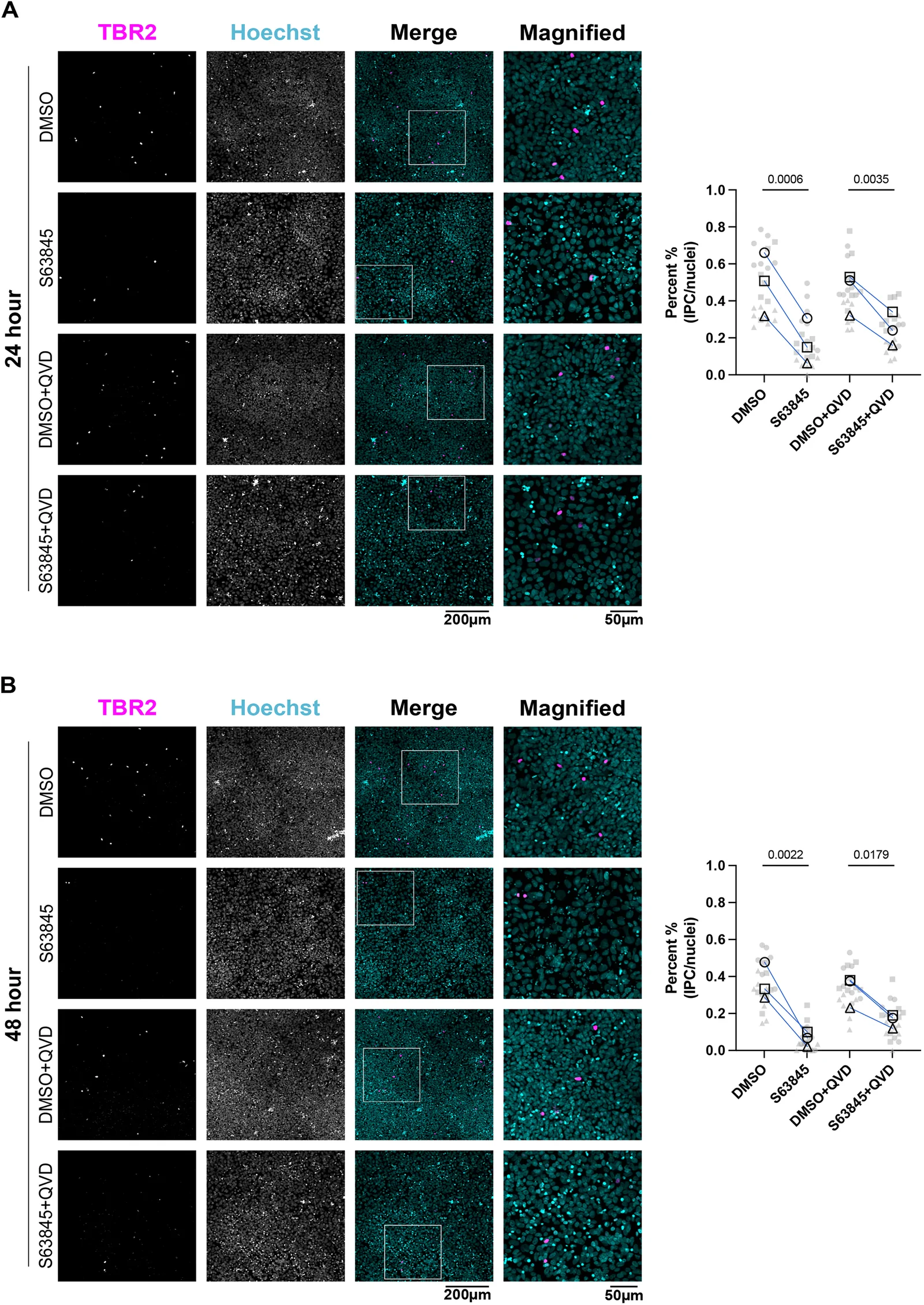
**S63845 treatment results in depletion of the TBR2^+^ PAX6^−^ IPC pool.** (A,B) Immunofluorescence images of TBR2 (magenta) and nuclei (Hoechst, cyan) were acquired on a spinning-disc confocal microscope at 20×. Left: representative images of NPCs treated with S63845 (MCL-1 inhibitor) with or without QVD (caspase inhibitor) for 24 h (A) and 48 h (B). Boxes in merge images indicate regions shown in magnified images. Right: quantification of TBR2^+^ IPCs calculated as percentage of total cell population for 24 h (A) and 48 h (B) treatments. Each large, open data point (black) represents the mean of a biological replicate, and each small, closed data point (transparent gray) represents a technical replicate (a single image). Shape of the data points corresponds to a biological replicate (*n*=3; circle for replicate 1, square for replicate 2, and triangle for replicate 3). Data were analyzed using a repeated measures two-way ANOVA with an uncorrected Fisher's LSD test for multiple comparisons and a 95% confidence level (*P*≤0.05) as the significance threshold. *P*-values are indicated in the figure. Error bars represent s.e.m.

**Fig. 8.**
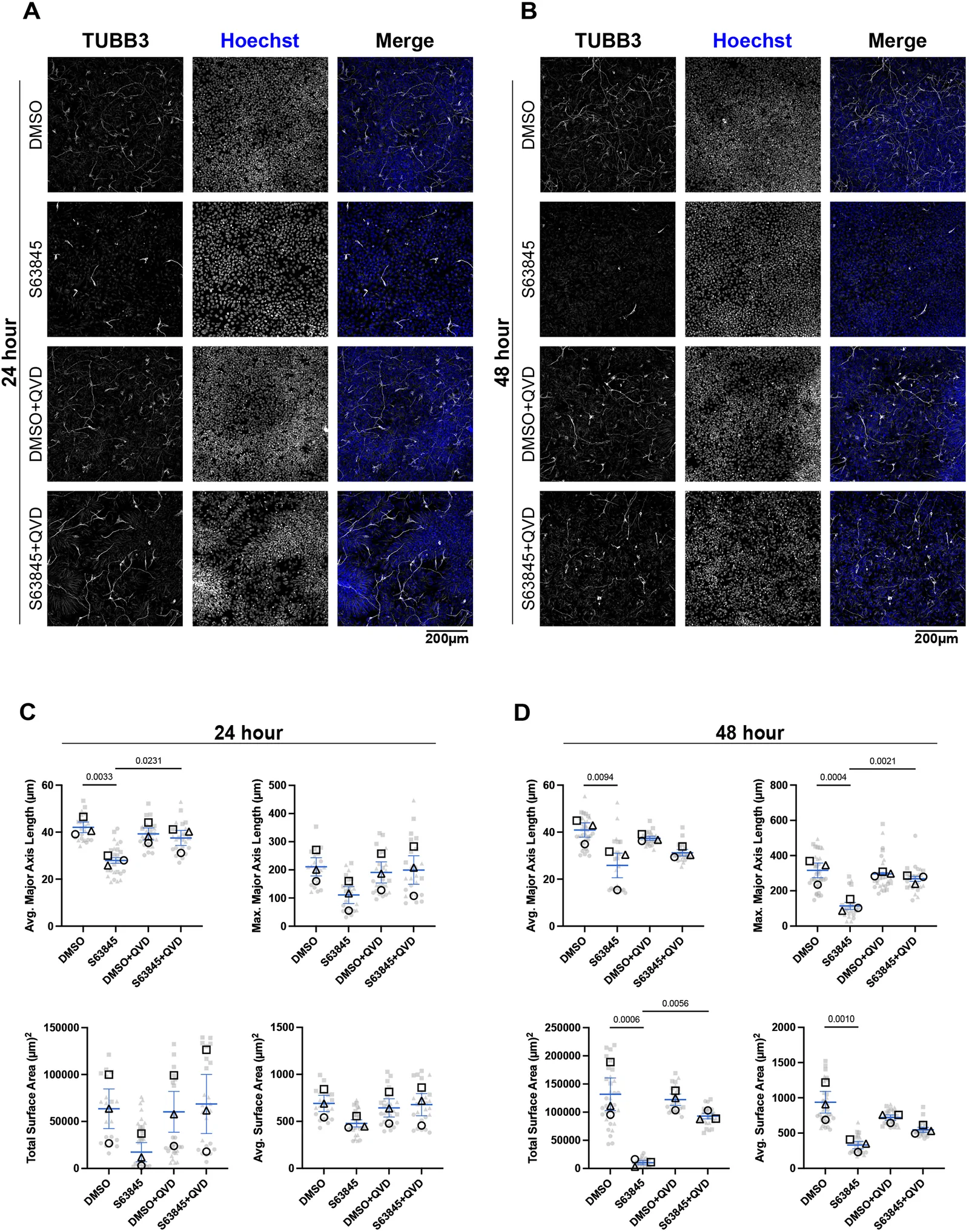
**S63845 treatment results in the depletion of newborn neurons detected by βIII-tubulin.** (A,B) Immunofluorescence images of TUBB3 (white) and nuclei (Hoechst, blue) were acquired on a spinning-disc confocal microscope at 20×. Representative images (*n*=3) of NPCs treated with S63845 (MCL-1 inhibitor) with or without QVD (caspase inhibitor) for 24 h (A) and 48 h (B). (C,D) Quantified morphology analysis of TUBB3 parameters, including average (Avg.) and maximum (Max.) major axis length and total and average surface area after 24 h (C) and 48 h (D) treatments. Each large, open data point (black) represents the mean of a biological replicate, and each small, closed data point (transparent gray) represents a technical replicate (a single image). Shape of the data points corresponds to a biological replicate (*n*=3; circle for replicate 1, square for replicate 2, and triangle for replicate 3). Data were analyzed using an ordinary two-way ANOVA with an uncorrected Fisher's LSD test for multiple comparisons and a 95% confidence level (*P*≤0.05) as the significance threshold. *P*-values are indicated in the figure. Error bars represent the s.e.m.

## DISCUSSION

The non-apoptotic functions of MCL-1 in maintaining mitochondrial homeostasis are increasingly recognized across diverse cell types (Brinkmann et al., 2025; Rasmussen et al., 2018, 2020; Wright et al., 2024). Our findings establish MCL-1 as a critical coordinator of mitochondrial architecture and metabolic competence in human NPCs, extending this paradigm to the developing human brain. Although individual features of this phenotype have been reported in other model systems, their coordinated manifestation in a primary human neurodevelopmental context, and their functional consequence for progenitor identity, distinguishes this work from previous studies conducted in simplified or non-neural systems. By integrating ultrastructural, molecular and functional analyses, we demonstrate that MCL-1 sustains cristae integrity through stabilization of OPA1 isoform homeostasis and the MICOS complex, while simultaneously retaining ACSL1 at the OMM to enable fatty acid activation. Disruption of either arm of this coordinated function impairs FAO metabolism, reduces mitochondrial oxygen consumption, and ultimately compromises the identity of IPCs and neuronal maturation. Together, these findings reposition MCL-1 from a canonical anti-apoptotic protein to a structural and metabolic molecule that is indispensable for proper human neurogenesis.

A central question raised by our experimental approach is whether the phenotypes we describe reflect genuine non-apoptotic functions of MCL-1 or instead represent downstream consequences of mitochondrial outer membrane permeabilization (MOMP) that persist despite caspase inhibition. While QVD blocks caspase activation, it does not prevent upstream BAX and BAK (BAK1) activation, cytochrome *c* release, or MOMP itself, and MOMP has traditionally been considered a committed, uniformly lethal event. However, live-cell imaging studies have shown that MOMP is not always complete or synchronous within a cell: a subset of mitochondria can remain unpermeabilized, and the persistence of this intact mitochondrial pool is associated with cellular recovery when caspase activity is blocked (Ichim et al., 2015). This raises the possibility that caspase-inhibited cells retain a functional mitochondrial population capable of supporting the phenotypes we describe, rather than being uniformly locked into an unresolved MOMP state. A direct genetic test of this question, restricted to a BAX and BAK-null background, is complicated by the fact that we have previously shown that BAX and BAK double-knockout hiPSC-derived cortical cells, which cannot undergo MOMP, display abnormal mitochondrial morphology and aberrant cortical structure on their own (Joshi et al., 2020), indicating that the core apoptotic machinery contributes to mitochondrial structural integrity independently of MOMP kinetics and complicating interpretation of this genetic model. Together, these considerations support our interpretation that the cristae and mitochondrial ultrastructural phenotypes reported here are unlikely to solely reflect an ongoing, unresolved MOMP process, though direct assessment of BAX and BAK activation status in our system remains an important goal for future work.

While MCL-1 has been reported to influence FAO in cancer cells and to localize to the IMM (Escudero et al., 2018; Wright et al., 2024; Perciavalle et al., 2012), its impact on cristae integrity in neural progenitors remained unexplored. Our findings bridge this gap by showing that MCL-1 is necessary for the maintenance of cristae organization and FAO competence in NPCs. The observed mitochondrial morphological swelling upon MCL-1 inhibition likely reflects a compensatory response to the loss of metabolic function, as evidenced by lipid droplet accumulation and impaired oxygen consumption. We find this disruption to be coupled to a decline in FAO enzymes. Consistent with findings in cancer cells (Wright et al., 2024), we demonstrate that MCL-1 inhibition causes displacement of ACSL1 from the OMM in NPCs. Given that all altered FAO enzyme levels were downstream of ACSL1, this raises the question of whether ACSL1 represents a rate-limiting step in the mitochondrial FAO pathway in NPCs. If ACSL1 is displaced from the mitochondria, this may disrupt a feedback loop that maintains the expression and stabilization of downstream FAO enzymes, thereby dismantling the FAO cascade and driving lipid accumulation. The convergence of ACSL1 delocalization with cristae structural defects suggests that MCL-1 coordinates fatty acid activation at the OMM and oxidative capacity at the IMM through spatially distinct but functionally coupled mechanisms. We note that this conclusion is based on imaging of an exogenously expressed ACSL1–HaloTag fusion and should be considered suggestive rather than definitive; biochemical approaches such as co-immunoprecipitation, together with functional confirmation of the fusion protein, will be needed to more rigorously establish altered MCL-1–ACSL1 association.

Beyond the broad transcriptional suppression of FAO genes previously reported upon MCL-1 deletion in cancer cells (Prew et al., 2022), our data indicate post-translational mechanisms, including altered protein stability, as the primary drivers of FAO enzyme dysregulation in NPCs. This distinction is conceptually important: it suggests that in neural progenitors, MCL-1 supports FAO enzyme function primarily through structural maintenance of the mitochondrial membrane environment rather than through transcriptional control and raises the possibility that the cristae collapse further impairs the stability and localization of membrane-associated FAO enzymes.

We propose a model in which MCL-1 operates through two coordinated and caspase-independent mechanisms to sustain mitochondrial function in NPCs. At the OMM, MCL-1 retains ACSL1 to enable fatty acid activation upstream of CPT1 and CPT2, consistent with the direct BH3-like interaction between MCL-1 and ACSL1 previously identified in cancer cells. At the IMM, MCL-1 preserves cristae integrity through stabilization of OPA1 isoform stoichiometry and the MICOS complex, maintaining the structural platform required for OXPHOS and FAO enzyme function. The combined loss of OMM-level fatty acid activation and IMM-level cristae integrity and respiration infers that these functions operate in parallel and require the non-apoptotic capacities of MCL-1. Distinguishing the direct OMM scaffolding role of MCL-1 for ACSL1 from potential indirect effects of cristae collapse on ACSL1 retention will require future studies using structure-specific MCL-1 mutants or domain-selective approaches.

The metabolic intermediates generated by FAO, particularly acetyl-CoA, are well-established regulators of chromatin state through their role as substrates for histone acetyltransferases (Martínez-Reyes and Chandel, 2020). A reduction in mitochondrial acetyl-CoA availability secondary to FAO impairment could alter histone acetylation at neurogenic loci, thereby influencing transcriptional programs that govern progenitor identity and lineage commitment. This metabolic–epigenetic axis represents a plausible mechanism by which MCL-1-dependent FAO supports the maintenance of IPCs and constitutes a testable model for future investigation. In the context of neurodevelopment, where bioenergetically demanding progenitor cells must maintain a precise metabolic profile, mitochondrial competence functions as a quality control checkpoint for lineage progression. We emphasize that this metabolic–epigenetic link, along with the broader question of how MCL-1 inhibition mechanistically alters differentiation programs, remains an open question that our current data do not resolve. Identifying the downstream pathways responsible for the differentiation phenotype we describe, for example through transcriptomic profiling or targeted pathway inhibition, is an important direction for follow-up work rather than a mechanistic conclusion supported by the present study.

The clinical precedent established by venetoclax, a selective BCL-2 inhibitor approved for hematologic malignancies (DiNardo and Konopleva, 2021; Kater et al., 2025), underscores both the therapeutic potential and the selectivity challenges inherent to targeting BCL-2 family members. The potential cardiotoxicity and emerging neural concerns associated with MCL-1 inhibitors, relative to the comparatively favorable central nervous system tolerability profile of venetoclax, reinforce the notion that BH3-mimetic selectivity can be influenced by tissue-specific factors. Our data suggest that cells undergoing active neurogenesis are particularly vulnerable to MCL-1 inhibition because they rely on the non-apoptotic roles of MCL-1 rather than its pro-survival function, a distinction that the clinical experience with venetoclax does not capture and that could be critical when evaluating MCL-1-targeting agents in oncology regimens.

The contribution of BCL-XL to mitochondrial bioenergetics provides an important parallel for interpreting our findings. BCL-XL has been shown to interact directly with subunits of the ETC, particularly complexes I and III, enhancing proton flux efficiency and increasing ATP synthesis independently of its anti-apoptotic function (Chen et al., 2011; Park et al., 2015). These studies have established the precedent that BCL-2 family members can act as direct modulators of ETC stoichiometry and complex assembly, factors that MCL-1 activity in the IMM could also modulate. Revealing the contribution of MCL-1 to these critical aspects of mitochondrial function will require profiling respiratory complex assembly under MCL-1 inhibition to help clarify whether impaired respiration in NPCs is also due to direct effects on the ETC.

The mechanism by which MCL-1 inhibition destabilizes OPA1 isoform stoichiometry likely converges on the inner membrane proteases OMA1 and YME1L, which execute opposing cleavage events to regulate the balance between long and short OPA1 isoforms (Anand et al., 2014). Under basal conditions, YME1L-mediated constitutive processing of OPA1 is balanced against stress-induced OMA1 activation, which rapidly converts long OPA1 isoforms to short forms and thereby promotes cristae widening and cytochrome *c* release competence. Mitochondrial membrane depolarization and matrix stress are well-established triggers for OMA1 hyperactivation (Rainbolt et al., 2016). The metabolic impairment and FAO failure we observe upon MCL-1 inhibition could create an environment conducive to OMA1 activation, shifting the OPA1 equilibrium toward short isoforms and destabilizing the cristae junctions that MCL-1 normally maintains. MCL-1 is likely targeted for degradation by YME1L, but whether OPA1 cleavage imbalance is a downstream consequence of the broader metabolic failure we describe remains an important open question for future mechanistic studies.

The influence of mitochondrial architecture on developmental fate decisions is increasingly recognized. *In vivo* and *in vitro* studies have identified a postmitotic window of neurogenic plasticity controlled by mitochondrial morphology (Iwata et al., 2020), and that disruption of the FAO pathway promotes asymmetric differentiation of adult mouse pluripotent and neural stem cells, underscoring the importance of mitochondrial morphology and FAO in stem cell fate regulation (Knobloch et al., 2017). Our data reveal that MCL-1 inhibition disrupts this balance through mechanisms extending beyond FAO itself, implicating cristae architecture and ACSL1-mediated lipid activation as additional determinants of progenitor identity. Importantly, mutations in cristae-shaping proteins, including OPA1 and MICOS subunits, are associated with severe neurodevelopmental and neurodegenerative disorders such as dominant optic atrophy, and Parkinson's disease-associated neurodegeneration (Eramo et al., 2020). The mechanistic parallels between these genetic disorders and the phenotypes induced by MCL-1 inhibition in NPCs reinforce the notion that mitochondrial function is indispensable for these bioenergetically demanding neural cells.

Taken together, our results position MCL-1 upstream of OPA1, MICOS and ACSL1, and define a previously unappreciated dimension of MCL-1 function in human neurogenesis. MCL-1 is emerging as a metabolic modulator coordinating cristae architecture, ACSL1 mitochondrial localization, lipid oxidation, mitochondrial respiration and lineage progression, all independently of its canonical anti-apoptotic role. These findings carry potential translational relevance. MCL-1 inhibitors have been explored as therapeutic agents for chemoresistant malignancies, but clinical development has been limited by cardiotoxicity. We have previously reported that MCL-1 compromises cytoskeletal integrity in cardiomyocytes as detected by phalloidin (Rasmussen et al., 2020), and here we report failed maturation of newborn neurons as detected by cytoskeletal marker, TUBB3. Our data suggest that the non-apoptotic consequences of MCL-1 inhibition in neural progenitors require consideration when evaluating the neurodevelopmental safety of BH3-mimetic therapies, particularly in pediatric contexts when active neurogenesis is ongoing. Further investigation into how MCL-1 coordinates cristae organization and lipid metabolism in progenitor cells may illuminate new vulnerabilities in neurodevelopmental and neurodegenerative disorders.

### Limitations

Using caspase inhibitors to assess apoptosis is a limitation; ideally, apoptosis would be more specifically blocked by targeting BAX and BAK. However, these cells exhibit dramatic changes in mitochondrial morphology that would confound results (Joshi et al., 2020). We reasoned that phenotypes detected with S63845 alone, but not with S63845 co-treatment with QVD, are rescued by inhibition of apoptosis, suggesting they are attributable to the canonical anti-apoptotic functions of MCL-1. Conversely, phenotypes detected with S63845 and QVD co-treatment, but not with S63845 alone, likely represent a snapshot of cellular changes occurring in vulnerable cells before they undergo apoptosis and are therefore also attributable to the canonical anti-apoptotic role of MCL-1. Finally, phenotypes detected under S63845 that persist with QVD co-treatment, thus not rescued by caspase inhibition, are attributed to non-apoptotic functions of MCL-1. We prioritized QVD for assays where caspase-dependent effects were most likely to confound interpretation of non-apoptotic phenotypes and omitted it in other assays because of factors such as experiment duration, compatibility with downstream readouts or prior validation showing that its inclusion did not alter outcomes in related experiments. Our inference of impaired FAO relies on protein-level changes in FAO enzymes, lipid droplet accumulation and oxygen consumption; direct biochemical confirmation through isotope-labeled palmitate tracing would provide more definitive evidence of altered fatty acid oxidative flux and is an important goal for follow-up studies.

In this study, we aimed to disrupt the BH3 binding domain of MCL-1 through pharmacological inhibition using S63845. The small-molecule inhibitor S63845 selectively occupies the BH3-binding groove of MCL-1, blocking its protein–protein interactions while preserving MCL-1 protein levels (Kotschy et al., 2016). This approach allowed us to specifically investigate the consequences of BH3 domain blockade in isolation from broader transcriptional and translational adaptations that accompany genetic depletion. As demonstrated by our immunoblot data, S63845 treatment increases total MCL-1 protein levels, consistent with stabilization of the protein when its BH3 domain is occupied. This approach also holds direct clinical relevance, as S63845 and related BH3 mimetics are pharmacological agents under therapeutic investigation, making the phenotypes described here translatable to the consequences of therapeutic MCL-1 inhibition in human neural cells. Nevertheless, we acknowledge that stabilized but inhibited MCL-1 may retain partial scaffolding or structural functions at the IMM or OMM that would be absent in a true loss-of-function model. Future studies using genetic swap tools (Brinkmann et al., 2025) or inducible genetic deletion of MCL-1 in NPCs will be valuable for distinguishing the consequences of BH3 domain blockade from complete loss of MCL-1 protein, and for determining whether the mitochondrial structural and metabolic phenotypes described here are fully recapitulated under conditions of genetic loss of function. A further challenge in fully dissecting the distinct contributions of MCL-1 at the OMM and IMM is the lack of tools that selectively restrict MCL-1 localization to one membrane compartment without perturbing the protein itself. Current approaches to enforce IMM-restricted localization rely on adding exogenous targeting domains (Perciavalle et al., 2012), which may alter MCL-1 conformation, interaction partners or function. Developing refined tools that enable compartment-specific interrogation of MCL-1 function without modifying the endogenous protein is an important priority for the field.

Lastly, our findings on the IPC pool depletion upon S63845 treatment strongly suggest a unique reliance on MCL-1. However, the differentiation protocols used to generate neural progenitor cultures *in vitro* yield a minority pool of TBR2-expressing IPCs that constitutes fewer than 1% of the total cell population. Before investigating the effects of S63845 on this cell type, we optimized IPC derivation from various human pluripotent stem cell (hPSC) lines. Each study should identify the stage at which the TBR2-expressing population reaches maximal expansion during hPSC differentiation to NPCs, as hESC and hiPSC lines exhibit variable competence in generating TBR2-positive IPCs, and the timing of peak generation varies across lines. These limitations highlight the need to develop methods to isolate and expand the IPC population from hPSCs, which would be a valuable tool for future studies.

## MATERIALS AND METHODS

### Human embryonic stem cells

The hESC line, H9 (WA09), was obtained from WiCell Research Institute (Wisconsin). H9s were maintained in mTeSR (STEMCELL Technologies, 85850) medium on 6-well plates coated with Matrigel (Corning, 354277) at 37°C with 5% CO_2_. Culture medium was changed daily. Cells were checked daily for differentiation and were passaged every 3–4 days using Gentle cell dissociation solution (STEMCELL Technologies, 07174). All experiments were performed under the supervision of the Vanderbilt Institutional Human Pluripotent Cell Research Oversight (VIHPCRO) Committee. Cells were periodically checked for contamination.

### hESC-derived NPC culture

hESCs (H9) were routinely characterized for pluripotency and tested for contamination. Cells were cultured to 70% to 80% confluency with minimal differentiation at colony edges, then dissociated with 1 ml of Gentle Cell Dissociation Reagent (STEMCELL Technologies, 100-0485) at 37°C for 8 min. Then, 2 ml of DMEM-F12 (Thermo Scientific, 11320033) supplemented with 10 µM ROCK inhibitor (STEMCELL Technologies, 72307) was added to each well, and cells were gently scraped, transferred to a conical tube and centrifuged at 300 ***g*** for 5 min. The pellet was resuspended in STEMdiff SMADi Neural Induction Medium (NIM; STEMCELL Technologies, 08581) supplemented with 10 µM ROCK inhibitor, counted and plated at 2.0×10⁶ to 2.5×10⁶ cells per well in a 6-well plate. Cells were maintained at 37°C for 7 days with daily NIM feeding. Where indicated, NPCs were treated with 1 µM MCL-1 inhibitor (MedKoo, 406849), 100 µM palmitate (Cayman Chemical Company, 29558) or 25 µM QVD (SM Biochemicals, SMPH0001), or starved in HBSS (Thermo Scientific, 14175095) for 2 h.

#### Lentivirus cloning and production

A lentiviral vector kindly provided by the Brunger lab (Vanderbilt University, Nashville, TN, USA), containing an EFf1α promoter under puromycin resistance, was used as the lentiviral vector backbone. The pCVMV-ACSL1 C-terminally tagged HaloTag plasmid, provided by the Opferman lab (St. Jude Children's Research Hospital, Memphis, TN, USA), was used as the template. Primers for the ACSL1–HaloTag insert (Molecular Cell Biology Resource Core's Integrated DNA Technologies portal) were: forward, 5′-GTAATAGCGGCCGCATGCAAGCCCATGAGCTGT-3′; and reverse, 5′-GGCAGCGCTTAATTAAGCCGGAAATCTCGAGCGTC-3′. Primers were designed with overhangs including NotI and PacI restriction cut sites found in the multiple cloning site (MCS) of the lentiviral vector. A polymerase chain reaction (PCR) of 35 cycles was performed on the ACSL1–HaloTag plasmid using the primers above and the Q5 Hot Start High-Fidelity 2× Master Mix (New England Biolabs, M0494S). The PCR cycle included a 95°C denaturation step, a 64°C annealing step and a 72°C extension step at 15 s/kb. The PCR was followed by a double restriction digest of NotI (New England Biolabs, R3189L) and PacI (New England Biolabs, R0547L) on the PCR product of the ACSL1–HaloTag plasmid and the lentiviral vector backbone at 37°C for 1 h. The digest was purified via gel extraction (Takara Bio, 740609.250), quantified via Nanodrop, and a 1:3 vector-to-insert molar ratio was used for the ligation step. The digested ACSL1–HaloTag insert and lentiviral backbone were incubated with T4 DNA Ligase (New England Biolabs, M0202L) overnight at 16°C. The next day, the ligation reaction was treated with Exonuclease V (New England Biolabs, M0345S) for 30 min at 37°C. The treated ligation product was used for transformation of Mix & Go! DH5 Alpha Competent Cells (Zymo Research, T3007). Bacteria were plated on 100 μg/ml ampicillin Luria–Bertani (LB) agar plates, incubated overnight at 37°C. The following day, bacterial colonies were picked and grown in 10 ml of LB broth overnight at 37°C, followed by a QIAprep Spin Miniprep (Qiagen, 27104) to isolate plasmid DNA. Sequencing of the clones was performed by GeneWiz, and the corrected lentiviral clones with the ACSL1–HaloTag insert were selected and expanded using the QIAGEN Plasmid Midi Kit (Qiagen, 12143). The pLV-EF1a-ACSL-1-HaloTag-PGK-Puro plasmid is available through Addgene (Addgene plasmid 257893).

#### Lentiviral production

A 6-well plate was seeded with 700,000 HEK293T cells (Clontech) per well by plating 350,000 cells/ml after dissociating with TrypLE Express Enzyme (Thermo Fisher Scientific, 12604013). Upon HEK293T cells reaching ∼70–80% confluence, Lipofectamine 3000 (Thermo Fisher Scientific, L3000015) was used to transfect 2.0 μg of EF1α ACSL1–HaloTag plasmid, 1.5 μg of pCMVdR8.91 gag/pol packaging plasmid (Creative Biogene, OVT2971) and 0.6 μg of pMD2.G envelope plasmid (Addgene plasmid 12259), following the manufacturer's instructions. The DNA lipid complex was added dropwise to HEK293T cells cultured with DMEM/F-12 medium (Thermo Fisher Scientific, 11-330-057) and fresh 10% heat-inactivated fetal bovine serum (FBS) (Sigma Aldrich, F2442-500ML). One day after transfection, the medium was replaced with 2 ml of Opti-MEM (Gibco, 31985070). On days 2 and 3 following transfection, viral medium was collected and filtered with a 0.45 μm PES filter (EMD Millipore, SLFH05010). The supernatant was combined with one volume of Lenti-X Concentrator with three volumes of clarified supernatant, mixing by gentle inversion and incubating overnight at 4°C. The sample was centrifuged at 1500 ***g*** for 45 min at 4°C. The pellet was resuspended in 550 µl of Opti-MEM medium, and aliquots were stored at −80°C for long-term storage.

#### Transduction

For transduction of hESCs, one confluent 35 mm well was dissociated by incubation with 1 ml of Gentle Cell Dissociation Reagent (Stem Cell Technologies, 100-0485) for 8 min at 37°C. Dissociation was quenched with 2 ml of DMEM/F-12 supplemented with 10 μM ROCKi (Stem Cell Technologies, 72307), and cells were collected into a 15 ml conical tube. Cells were pelleted by centrifugation at 200 ***g*** for 4 min, the supernatant was aspirated, and the pellet was resuspended in 1 ml of mTeSR1 (Stem Cell Technologies, 85857) supplemented with 10 μM ROCKi. Lentivirus was added at a final concentration of 5% (v/v) with 4 µg/ml of polybrene (Sigma Aldrich, H9268-5G), cells were plated onto a 24-well plate and incubated overnight at 37°C (16–18 h).

The following morning, wells were replenished with fresh mTeSR1 and incubated overnight at 37°C. After 48 h, antibiotic selection began with puromycin at 10 μg/ml (Stem Cell Technologies, 73342). The medium was replaced daily with puromycin-containing mTeSR1, and cells were passaged as needed to maintain selection.

### Immunoblot analysis

Cells were harvested in lysis buffer containing 1% Triton X-100 (Sigma, T9284), 1× complete protease inhibitor cocktail (Roche, 04693159001), 1× PhosSTOP phosphatase inhibitor (Roche, 04906837001) and 0.5 mM PMSF (RPI, P20270). Samples were kept on ice and vortexed for 30 s every 10 min. Lysates were centrifuged at 14,100 ***g*** for 30 min at 4°C. Supernatant was collected, and protein concentrations were quantified using the Pierce BCA Protein Assay Kit (ThermoScientific, 23225). Western blotting was performed using the Bio-Rad Mini-PROTEAN Electrophoresis System (Bio-Rad, 1658036). Samples were run on 4–20% Mini-PROTEAN TGX gels (Bio-Rad, 4561095). Protein was transferred from gels onto methanol-activated PVDF membranes (Bio-Rad, 1620177). All membranes were washed in Tris-buffered saline with 0.1% Tween-20 (TBST) and blocked in TBST containing 5% milk for 1 h at room temperature. Primary antibodies (Table S1) were incubated at the listed dilution in TBST containing 5% milk overnight at 4°C. Secondary antibodies were used at the listed dilution in TBST containing 5% milk and incubated for 1 h at room temperature. Protein signal was developed using either Pierce ECL Western Blot Substrate (32109, ThermoScientific) or SuperSignal West Femto (34094, ThermoScientific), depending on the sensitivity of the primary antibody. Protein signal was visualized using the Amersham Imager 600 (29-0834-61, GE Life Sciences). Acquired images were quantified using Thermo Fisher Scientific iBright Analysis Software and representative images were composed using Affinity Designer 2.

### RNA extraction and cDNA synthesis

Cells were collected in 1000 μl TRIzol reagent. 200 μl of chloroform was added, and the samples were incubated at room temperature for 5 minutes prior to centrifugation at 12,000 ***g*** at 4°C. The aqueous phase was collected, and 500 μl of isopropanol was added to precipitate RNA. Samples were incubated for 25 min at room temperature, followed by centrifugation at 12,000 ***g*** for 10 min at 4°C. The RNA pellet was washed with 75% ethanol, semi-dried and resuspended in 30 μl of DEPC-treated water. Concentration was measured, and 2 μg per sample was treated with DNAse (New England Biolabs, M0303) prior to generating cDNA using the manufacturer's protocol (Thermo Fisher, 4368814).

### RT-qPCR

50 ng of cDNA sample was used to run RT-qPCR using 20 μM of the primers listed in Table S2. QuantStudio 3 Real-Time PCR machine, SYBR green master mix (Thermo Fisher #4364346), and manufacturer instructions were used to set up the assay.

### Immunostaining

Cells were plated on 35 mm glass-bottom dishes (glass thickness of 0.16–0.19 mm) for confocal spinning-disk immunofluorescence. Cells were fixed with 4% paraformaldehyde for 20 min and permeabilized in 1% Triton-X-100 for 10 min at room temperature. After blocking in 10% bovine serum albumin (BSA) for 1 h, cells were treated with primary and secondary antibodies using standard methods. Primary antibodies were incubated at the listed dilution in 10% BSA overnight at 4°C (Table S1). Secondary antibodies were diluted in 10% BSA and incubated for 1 h at room temperature. The nuclear stain used was Hoechst 33342 (herein referred to as Hoechst; Thermo Scientific, H3570) and cells were mounted in Fluoromount-G (Electron Microscopy Sciences, 17984-25) prior to imaging. Cells were labeled for EdU (5-ethynyl-2'-deoxyuridine) using the manufacturer's protocol (Thermo Fisher, C10340). Lipid droplet staining was done live using BODIPY 493/503 (herein referred to as BODIPY; Cayman Chemical Company, 25892). Cells were incubated at 37°C for 15 min in 4 μM BODIPY prior to fixation in 4% paraformaldehyde.

### Spinning-disk confocal image acquisition

Images were acquired using a Nikon spinning-disk confocal microscope outfitted with a Yokogawa CSU-X1 spinning-disk head, Andor DU-897 EMCCD camera, high-speed piezo [*z*] stage, and Nikon Plan Apo VC 20×/0.75 NA and Plan Apo λ 100×/1.45 NA oil immersion objectives. All 100× images were acquired with a step size of 0.1 μm, whereas 20× images were acquired with a step size of 2 μm. Image processing and quantification was performed using NIS Elements (Nikon) and all representative images were generated using FIJI (https://imagej.net/software/fiji/) and NIS Elements (Nikon).

Quantification of mitochondrial morphology was performed in NIS-Elements version 5.42.06 (Nikon). Mitochondria were segmented in three dimensions, and data were extracted from the resulting three-dimensional mask. Major axis length, surface area, volume and count measurements were exported into Excel, and data were analyzed using GraphPad Prism v9.

### Tissue preparation for TEM and FIB–SEM

NPCs were plated at 1.5×10^6^ cells per 35 mm dish on Matrigel. The cells were washed three times with PBS, fixed in 2.5% glutaraldehyde in 0.1 M cacodylate prewarmed to 37°C, left to cool down at room temperature for 1 h, and placed at 4°C for an additional 24–48 h of fixation. Cells were washed from fixative three times with PBS. Samples were sequentially post-fixed using the OTO method: 1.5% potassium ferrocyanide, 1% OsO_4_ in 0.1 M cacodylate for 30 min on ice, then 1% thiocarbohydrazide for 10 min, followed by 1% OsO_4_ in double-distilled H_2_O for 10 min. Samples were then in-block stained with 2% uranyl acetate followed by lead aspartate. The samples were then dehydrated in a graded ethanol series and infiltrated with Durcupan resin (Electron Microscopy Sciences, 14040) using acetone as the transition solvent. The resin was polymerized at 60°C for 48 h. For TEM, sections were cut on a Leica UC7 at a nominal thickness of 50 nm with no post-section staining. For FIB-SEM, samples were prepared as previously described (Boassa et al., 2022).

### TEM image acquisition and cristae scoring

Sections were imaged with either a Thermo Fisher Scientific Tecnai T12 operating at 100 keV using an AMT nanosprint5 CMOS camera or a JEOL 2100+ operating at 200 keV using an AMT nanosprint15 II CMOS camera. In both cases, AMT acquisition software was used for imaging. The TEM images were analyzed using cristae scoring (Lam et al., 2021). The files were first renamed to anonymize the scoring process. The images were then scored by a researcher who was not aware of the sample identities. A score was provided for each mitochondrion based on the quality of the cristae. The scoring rubric was as follows: 0, there were no crista; 1, more than 50% of the mitochondrial area did not have cristae; 2, more than 25% of the mitochondria was without cristae; 3, lots of cristae but irregular in structure; and 4, many cristae that are regular structures.

### FIB–SEM image acquisition

Sections were imaged with Zeiss Crossbeam 550 FIB–SEM using ATLAS5 software for automated volumetric acquisition. Sample blocks were polished with a diamond knife and coated with platinum. Platinum pads were deposited via GIS on regions of interest, followed by milling in tracking marks and coating with GIS carbon. Milling was performed using a 700 pA Ga beam at 8 nm slice depth, imaging was performed with a 1.5 keV electron beam using the in-column ESB detector for imaging at 8 nm pixels to make isotropic voxels. Volumetric slice alignments were performed in ATLAS5 using the two-window alignment method.

### FIB–SEM mitochondrial image analysis

FIB–SEM stacks were first cropped using FIJI. In the FIB–SEM stacks, none of the cells imaged could be visualized completely. Therefore, the crop size and location were selected by choosing a cell with bright, visible mitochondria. The crop was 500×500 pixels with a 3000 nm depth. The cropped image was then imported into the FIJI LabKit plugin for segmentation (Arzt et al., 2022). For segmentation, each mitochondrion was manually traced with a separate label ID. Quality control was then performed following each mitochondrion through the entirety of the *Z*-stack and tracking all locations where the membranes connect. Any mitochondria that merged in any of the frames were labeled with the same label ID. The cropped FIB–SEM image and the proofread segmentation masks were imported into the IMARIS software. Ortho-slices in the *XY* and *XZ* planes were added, and the proofread mask was selected to be identified by ID. This identifies each separate mitochondrion. Any mitochondria that were still split were manually combined in the IMARIS software using the unify feature. A clipping plane was then added. Animations were made using the animations tab in IMARIS with 50 frames per second and 1000 frames total. Still images were captured using the screenshot feature. Volume quantification was done by volume measurement provided in the statistics tab in IMARIS. Surface area quantification was manually calculated using volume and sphericity measurements from the IMARIS statistics tab using the equation: 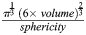. Lastly, the mitochondrial branching index (MBI), was calculated by obtaining the BoundingBox00 length A (longest side) and BoundingBox00 length C (shortest side) in the Vantage 2D tab and then dividing length C/length A manually (Vue et al., 2023).

### FIB–SEM cristae image analysis

The same FIB–SEM cropped images were used for the segmentation of the cristae. The cropped images were imported into FIJI, and Contrast Limited Adaptive Histogram Equalization (CLAHE) was applied to increase contrast. They were then imported into the LabKit plugin for segmentation. Each crista was manually traced, and all the cristae within one mitochondrion were labeled with the same label ID. Quality control was then performed, ensuring each mitochondrion had all its cristae labeled with the same ID. The cropped FIB–SEM image and the proofread segmentation masks were imported into the IMARIS software. Ortho-slices in the *XY* and *XZ* planes were added, and the proofread mask was selected by ID. This identifies the cristae of each separate mitochondrion. Any errors in the cristae mask were merged using the unify feature in IMARIS. A clipping plane was then added to show the mitochondria throughout the entire *z*-plane. Animations were made using the animations tab in IMARIS with 50 frames per second and with 1000 frames total. Still images were taken using the snapshot feature. Volume quantification was done by volume measurement provided in the statistics tab in IMARIS. Surface area quantification was manually calculated using volume and sphericity measurements from IMARIS statistics tab using the equation: 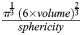. Cristae measurements were then normalized to the corresponding mitochondria by dividing the crista measurement by the mitochondrion measurement (i.e. 
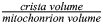
). The mitochondria segmentation was then added to the same IMARIS file as a second surface. The label was chosen by ID, and the surface type was changed to transparent to show the cristae within those mitochondria. Images were taken using the snapshot function, and animations were made with 50 frames per second and 1000 frames total.

### Statistical analysis

All experiments were performed with at least three biological replicates. Statistical significance was determined by one-way, repeated measures one-way ANOVA, or *t*-test as appropriate for each experiment. Significance was assessed using Fisher's protected least significance difference (LSD) test.

GraphPad Prism v9 was used for most statistical analyses and data visualization, unless otherwise specified in the figure legend. Error bars in all bar graphs represent the s.e.m. unless otherwise noted in the figure. No outliers were removed from the analyses. For all statistical analyses, a significant difference was accepted when *P*<0.05.

### Modeling *Ct* from RT-qPCR data

Cycles to threshold, *Ct*, measured using RT-qPCR can be modeled as previously shown (Hampton et al., 2025):
(1)

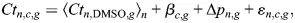
where *n* is the biological replicate, *c* is the treatment condition and *g* is the gene of interest. The first term, 〈*Ct*_*n*,DMSO,*g*_〉_*n*_, is *Ct* for gene *g* under control condition DMSO averaged across all biological replicates. The next term, coefficient *β*_*c*,*g*_, quantifies the effect of treatment *c* on gene *g*. Coefficient Δ*p*_*n*,*g*_=*p*_*n*,*g*_−*p*_1,*g*_ is a plate effect, allowing for differences between plates *n* for gene *g* (Sundberg et al., 2006). To maintain parameter identifiability, the plate effect is defined as the difference between plate *n* and plate *n*=1. Plate number is indicated by the same index as the biological replicates since each plate contains a single biological replicate. The remaining term 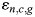 models unobserved random errors.

From this we derive the quantity of interest, ΔΔ*Ct*_*n*,*c*,*g*_, given by:
(2)

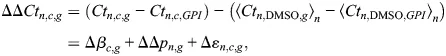
where Δ*β*_*c*,*g*_=*β*_*c*,*g*_−*β*_*c*,*GPI*_, ΔΔ*p*_*n*,*g*_=Δ*p*_*n*,*g*_−Δ*p*_*n*,*GPI*_, and 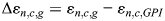. We fit this model to measured data using weighted linear regression with the Python package stats models (available at https://proceedings.scipy.org/articles/Majora-92bf1922-011). We assume that intra-gene variance is approximately homoscedastic, while inter-gene variance is heteroscedastic. Thus, weights are set at the gene level, giving:
(3)

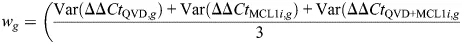
where *Var*() is the sample variance for gene *g* and indicated treatment across all biological replicates.

## Supplementary Material



10.1242/joces.265172_sup1 Supplementary information
