# Supplementary material for: Pharmacological inhibition of MCL-1 disrupts mitochondrial cristae and depletes the human neural progenitor cell pool
Source: J Cell Sci. 2026 Oct 15;139(19):jcs265172. doi: 10.1242/jcs.265172 (PMC13741951; doi:10.1242/jcs.265172)
Supplement: Supplementary file 1 [file joces-139-265172-s1.pdf]

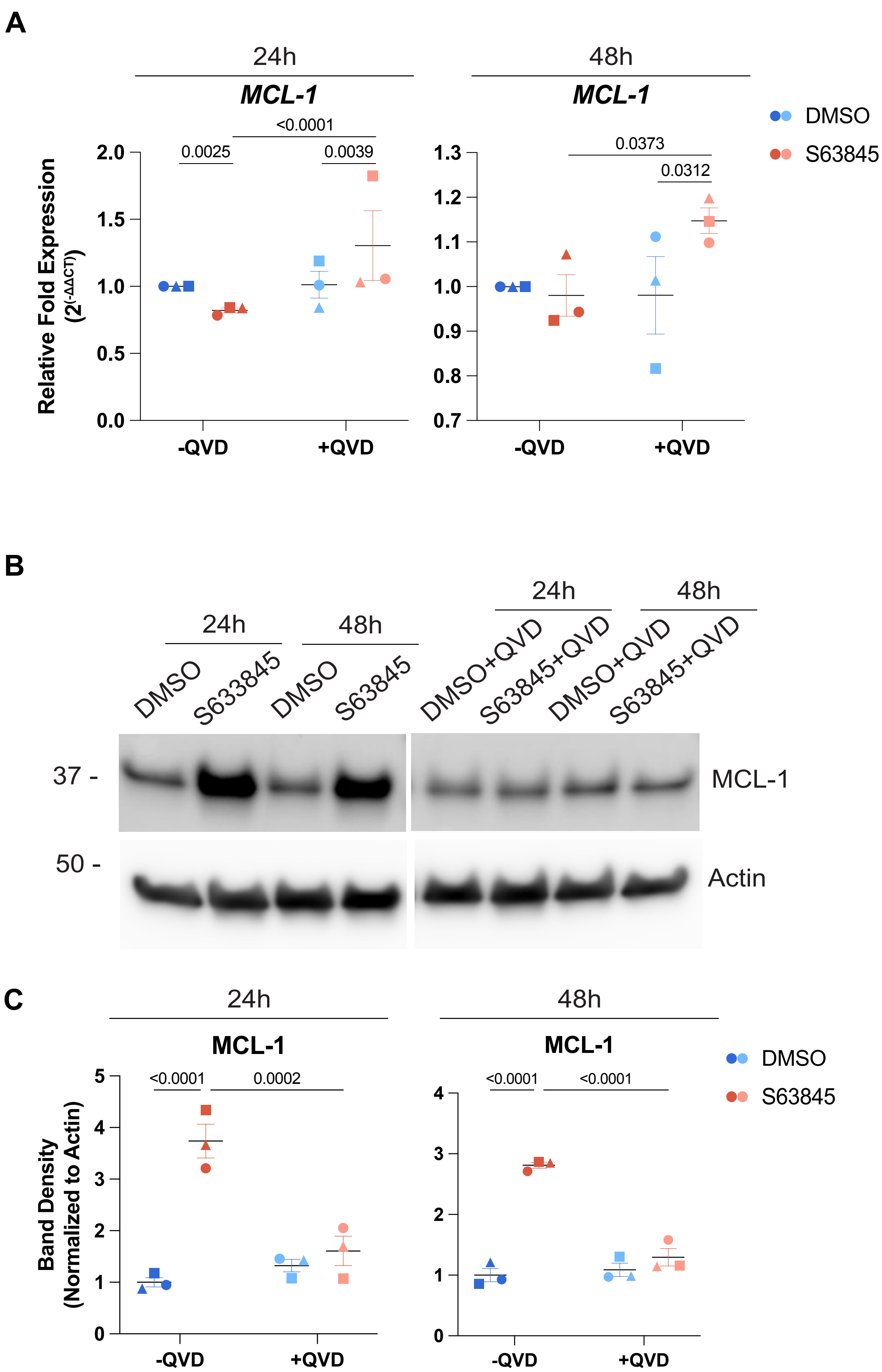

Journal of Cell Science • Supplementary information

Fig. S1. S63845 effectively inhibits MCL-1 in human NPCs. (**A**) Quantified relative expression measured by RT-qPCR of *MCL-1* after 24- and 48-hour treatment with S63845 (MCL-1 inhibitor) -/+ QVD (caspase inhibitor). Two technical replicates were used for all RT-qPCR data. RT-qPCR data are displayed as relative fold expression derived from $2^{(-\Delta\Delta CT)}$, and raw data were analyzed using ANCOVA as described in the methods section. (**B**) Representative images (n=3) . Note that the actin control blots for MCL-1 are shown again in Fig. 4B as the loading controls for VLCAD. (**C**) Quantified band density normalized to Actin loading control of MCL-1 after 24- and 48-hour treatments. Each data point on the graphs represents a biological replicate. Band density data were analyzed using an ordinary two-way ANOVA with an uncorrected Fisher's LSD test for multiple comparisons. Shape of the data points corresponds to a biological replicate (circle for n=1, square for n=2, and triangle for n=3). All tests used a confidence level of 95% (p-value ≤ 0.05 as the significance threshold). Error bars represent standard error of the mean.

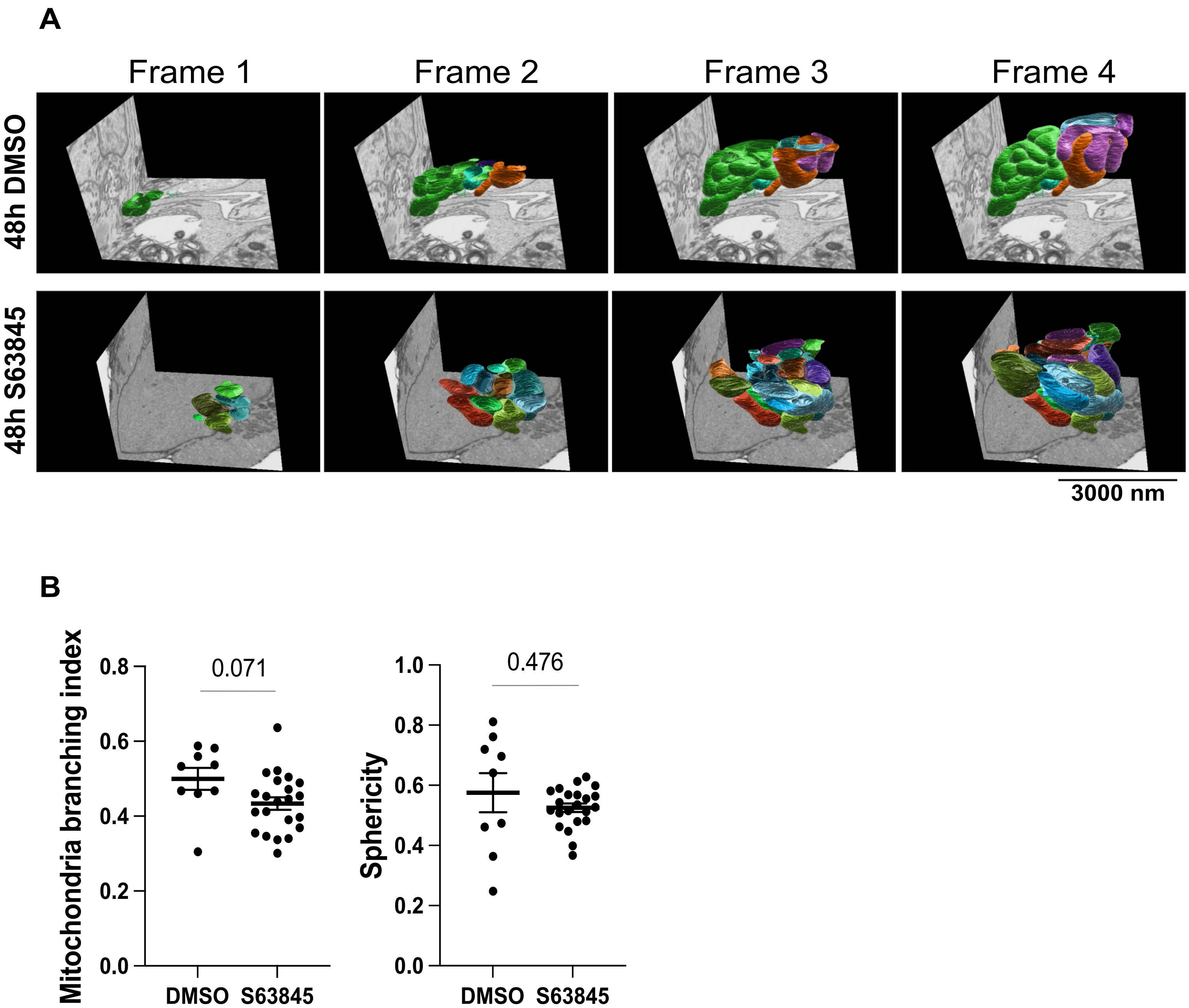

Fig. S2. S63845 does not alter mitochondria branching index and sphericity. (**A**) FIB-SEM 3-dimensional reconstruction of mitochondria after NPCs were treated with S63845 (MCL-1 inhibitor) for 48h (scale bar = 3000 nm). (**B**) Mitochondrial branching index (MBI) and sphericity were quantified as described in the methods section. Each data point represents an individual instance of a mitochondrion. Data analyzed using a parametric two-tailed unpaired t-test with Welch's Correction and a 95% confidence level (p-value ≤ 0.05 as the significance threshold). Error bars represent standard error of the mean.

Journal of Cell Science • Supplementary information

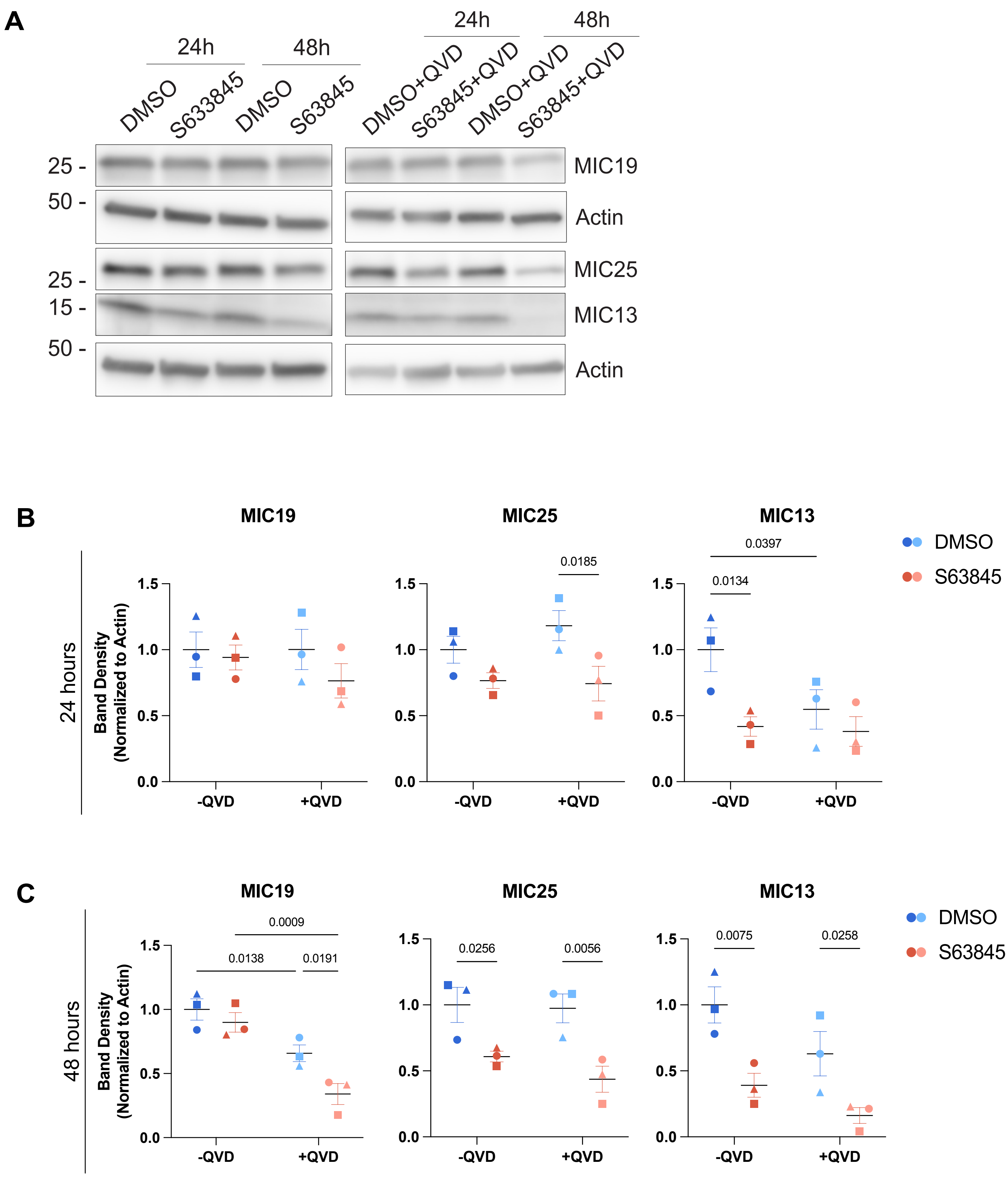

Fig. S3. S63845 induces changes subcomplex MICOS proteins. (A) Representative images (n=3). Note that the actin loading control blots for MIC19 and MIC25/MIC13 (no QVD) are the same as those in Fig. 3D for MIC10 and MIC60 (no QVD), respectively. The actin loading control for MIC19 (+QVD) is shown again in Fig. 3D as the loading control for MIC10 (+QVD). **(B,C)** Quantified band density normalized to Actin loading control of subcomplex MICOS proteins, MIC19, MIC25, and MIC13, after 24- **(B)** and 48-hours **(C)** of treatment with S63845 (MCL-1 inhibitor) -/+ QVD (caspase inhibitor). Each data point represents a biological replicate. Shaopf eth e data points corresponds to a biological replicate (circle for n=1, square for n=2, and triangle for n=3). Data were analyzed using ano rdinary two-way ANOVA with an uncorrectedF isher's LSD test for multiple comparisons and a 95% confidence level (p-value ≤ 0.05 as the significance threshold). Error bars represent standard error of the mean.

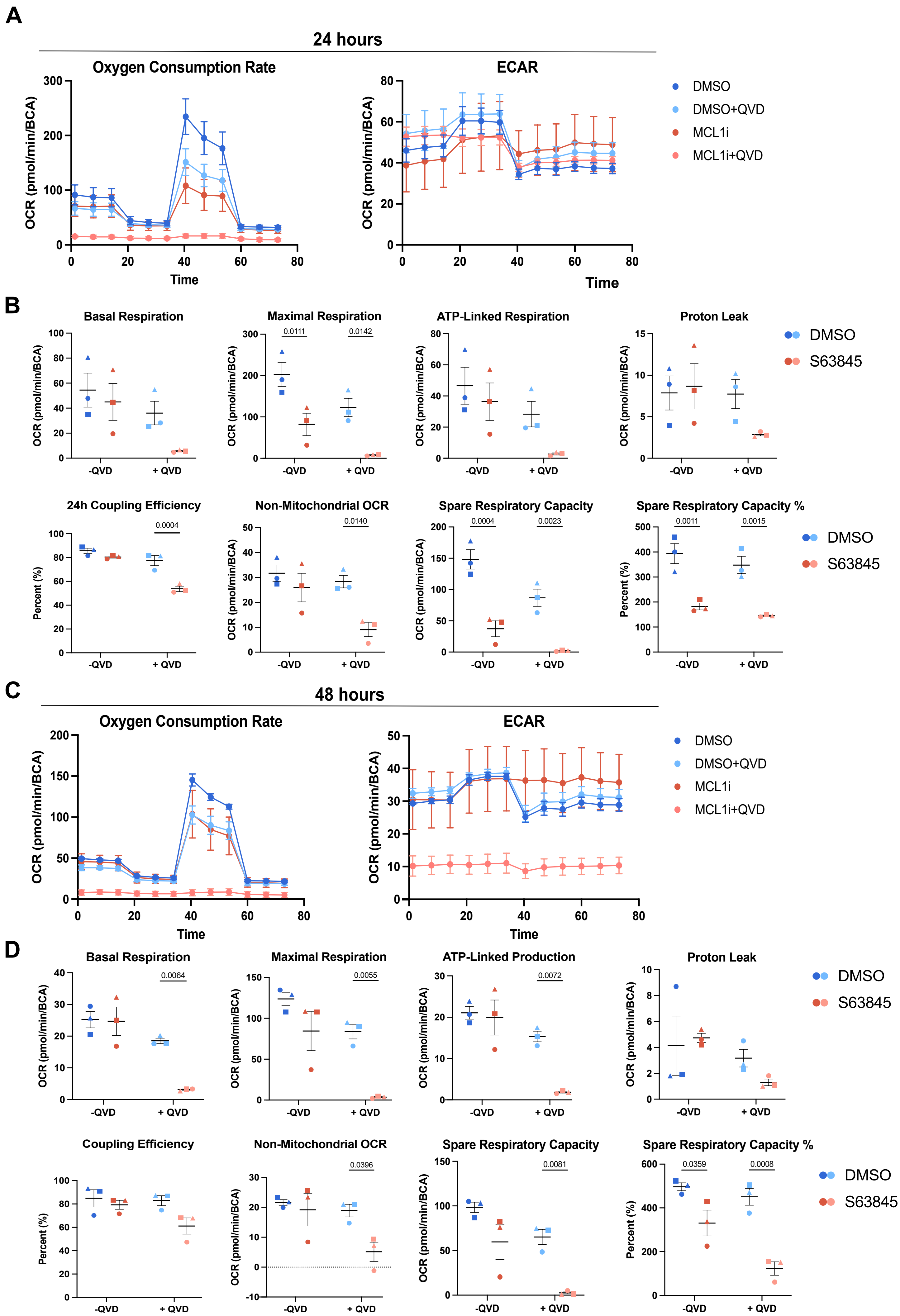

Journal of Cell Science • Supplementary information

Fig. S4. S63845 impairs mitochondrial respiration in human NPCs at 24 and 48 hours. (**A**) Oxygen consumption rate (OCR) and extracellular acidification rate (ECAR) traces over time following 24-hour treatment with S63845 (MCL-1 inhibitor) -/+ QVD (caspase inhibitor), measured by Seahorse XF Mitochondrial Stress Assay. (**B**) Quantified metabolic parameters derived from OCR trace for 24h treatments, including basal respiration, maximal respiration, ATP-linked respiration, proton leak, coupling efficiency, non-mitochondrial OCR, spare respiratory capacity, and spare respiratory capacity (%), shown for DMSO and S63845-treated cells -/+ QVD. (**C**) OCR and ECAR traces over time following 48-hour treatment with S63845 -/+ QVD. (**D**) Quantified metabolic parameters derived from OCR trace 48h treatments, including basal respiration, maximal respiration, ATP-linked production, proton leak, coupling efficiency, non-mitochondrial OCR, spare respiratory capacity, and spare respiratory capacity (%), shown for DMSO and S63845-treated cells -/+ QVD. Each data point on the graphs represents a biological replicate. Shape of the data points corresponds to a biological replicate (circle for n=1, square for n=2, and triangle for n=3). Data were analyzed using an ordinary two-way ANOVA with an uncorrected Fisher's LSD test for multiple comparisons and a 95% confidence level (p-value ≤ 0.05 as the significance threshold). Error bars represent standard error of the mean.

Journal of Cell Science • Supplementary information

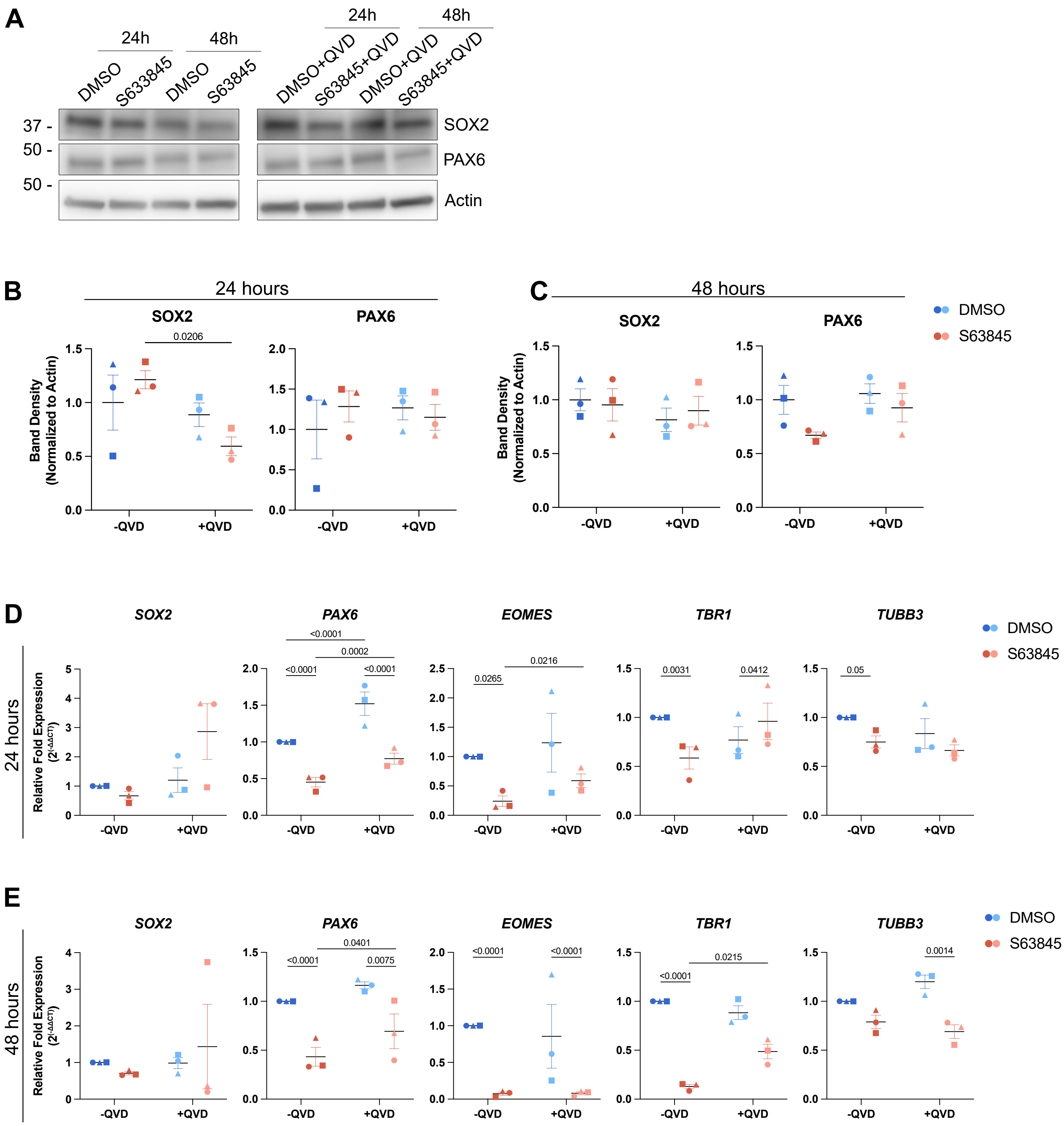
A
24h
48h
DMSO
S633845
DMSO
S63845
24h
48h
DMSO+QVD
S63845+QVD
DMSO+QVD
S63845+QVD
37 -
50 -
50 -
SOX2
PAX6
Actin
B
24 hours
SOX2
PAX6
0.0206
Band Density
(Normalized to Actin)
-QVD
+QVD
C
48 hours
SOX2
PAX6
DMSO
S63845
D
SOX2
PAX6
EOMES
TBR1
TUBB3
24 hours
Relative Fold Expression
<0.0001
0.0002
0.0265
0.0216
0.0031
0.0412
0.05
E
48 hours
0.0401
0.0075
0.0215
0.0014

Fig. S5. Downregulation of key NPC identity markers caused by S63845 persists in the absence of caspase-mediated cell death. (**A-C**) Representative images (n=3) and quantified band density normalized to Actin loading control of NPC identity markers, PAX6 and SOX2 after treatment with S63845 (MCL-1 inhibitor) -/+ QVD (caspase inhibitor). Band density data were analyzed using an ordinary two-way ANOVA with an uncorrected Fisher's LSD test for multiple comparisons. (**D,E**) Quantified relative expression measured by RT-qPCR of identity markers, *SOX2*, *PAX6*, *EOMES*, *TBR1*, and *TUBB3*, after 24- (**D**) and 48-hour (**E**) treatments. Two technical replicates were used for all RT-qPCR data. RT-qPCR data are displayed as relative fold expression derived from $2^{(-\Delta\Delta CT)}$, and raw data were analyzed using ANCOVA as described in the methods section. Each data point represents a biological replicate. Shape of the data points corresponds to a biological replicate (circle for n=1, square for n=2, and triangle for n=3). All tests used a confidence level of 95% (p-value ≤ 0.05 as the significance threshold). Error bars represent standard error of the mean.

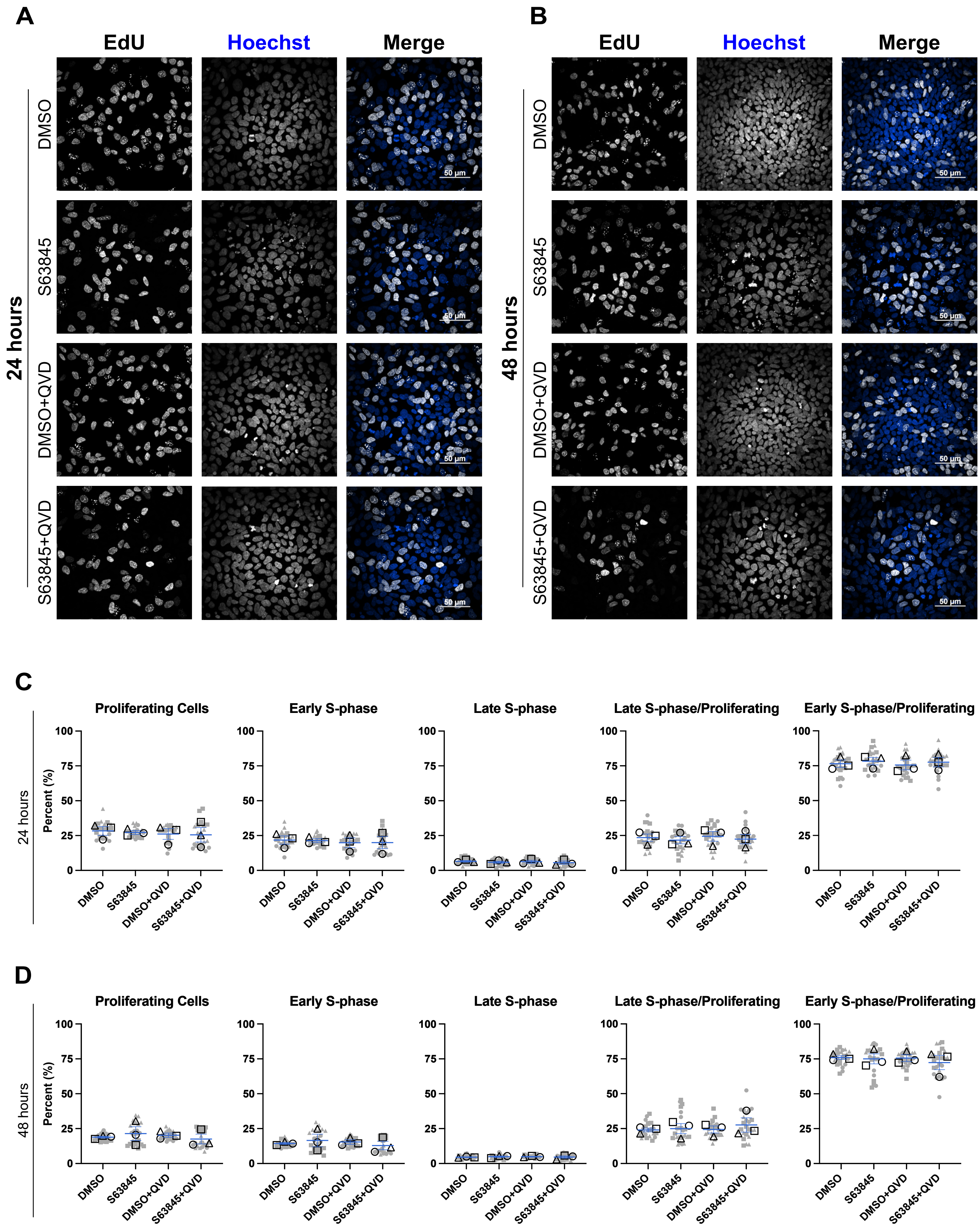

Journal of Cell Science • Supplementary information

Fig. S6. S63845 does not affect hNPC proliferation. (**A,B**) Immunofluorescent images of proliferating cells (EdU in white) and nuclei (Hoechst in cyan) acquired on SDC microscope at 60X (scale bar = 50μm). Representative images (n=3) of hNPCs treated with S63845 (MCL-1 inhibitor) -/+ QVD (caspase inhibitor) after 24- (**A**) and 48-hours (**B**). (**C,D**) Percent of EdU-labeled hNPCs after 24- (**C**) and 48-hour (**D**) treatments. Graphs from left to right: cell count of total proliferating cells, early S-phase (whole nuclear labeling), and late S-phase (punctate nuclear labeling) normalized to total cell count; cell count of early S-phase, and late S-phase normalized to total proliferating cells. Each large, opened-shape data point (black) represents the mean of a biological replicate, and each small, closed-shape data point (transparent gray) represents a technical replicate (a single image). Shape of the data points corresponds to a biological replicate (circle for n=1, square for n=2, and triangle for n=3). Data were analyzed using an ordinary two-way ANOVA with an uncorrected Fisher's LSD test for multiple comparisons and a 95% confidence level (p-value ≤ 0.05 as the significance threshold). Error bars represent standard error of the mean.

Journal of Cell Science • Supplementary information

Table S1. List of Antibodies

Target	Antibody Name	Host species	Vendor	Catalog Number	RRID	Application	Dilution
**ACSL1**	ACSL1 (D2H5)	rabbit	Cell Signaling Technology	9189S	AB_10891616	Western blot	1:1,000
**beta 3 Tubulin**	Beta III Tubulin	rabbit	Abcam	ab18207	AB_444319	IF	1:1,000
**beta Actin**	anti-b-Actin	mouse	Sigma-Aldrich	A1978	AB_476692	Western blot	1:50,000
**CPT1a**	anti-CPT1a	mouse	Proteintech	66039-01-Ig	AB_2920275	Western blot	1:5,000
**CPT2**	anti-CPT2	rabbit	Abcam	ab181114	AB_2687503	Western blot	1:1,000
**MCL-1**	anti-MCL-1	rabbit	Cell Signaling Technology	94296S	AB_2722740	Western blot	1:5,000
**MIC10**	MINOS1 (OTI4H7)	mouse	NovusBio	NBP2-45550	AB_3309700	Western blot	1:1,000
**MIC13**	anti-C19orf70	rabbit	Proteintech	25514-1-AP	AB_2880112	Western blot	1:1,000
**MIC19**	anti-CHCHD3	rabbit	Proteintech	25625-1-AP	AB_2687533	Western blot	1:1,000
**MIC25**	anti-CHCHD6	rabbit	Proteintech	20639-1-AP	AB_10697667	Western blot	1:1,000
**MIC60**	anti-IMMT	rabbit	Atlas Antibodies	HPA036164	AB_2674979	Western blot	1:5,000
**Mitochondria**	anti-mitochondria	mouse	Abcam	ab92824	AB_10562769	IF	1:1,000
**OPA1**	anti-OPA-1	rabbit	Cell Signaling Technology	67589S	AB_2799728	Western blot	1:5,000
**PAX6**	anti-PAX6	mouse	BD Biosciences	561462	AB_10715442	IF	1:500
**PAX6**	PAX6 (D3A9V) XP	rabbit	Cell Signaling Technology	60433S	AB_2797599	Western blot	1:5,000
**SOX2**	SOX2 (D6D9) XP	rabbit	Cell Signaling Technology	3579S	AB_2195767	Western blot	1:5,000
**TBR2**	anti-TBR2	rabbit	Millipore	AB2293	AB_10806889	IF	1:1,000
**VLCAD**	ACADVL	mouse	Thermo Fisher	PA5-29959	AB_2547433	Western blot	1:2,000

Journal of Cell Science • Supplementary information

Table S2. List of Primers

Target	Forward Primer	Reverse Primer
ACSL1	CCA TGA GCT GTT CCG GTA TTT	CCG AAG CCC ATA AGC GTG TT
CPT1a	TCC AGT TGG CTT ATC GTG GTG	TCC AGA GTC CGA TTG ATT TTT GC
CPT2	CTG GAG CCA GAA GTG TTC CAC	AGG CAC AAA GCG TAT GAG TCT
EOMES	GTG CCC ACG TCT ACC TGT G	CCT GCC CTG TTT CGT AAT GAT
GAPDH	ACA ACT TTG GTA TCG TGG AAG G	GCC ATC ACG CCA CAG TTT C
GPI	GTG TAC CTT CTA GTC CCG CC	GGT CAA GCT GAA GTG GTT GAA GC
MCL-1	GTG CCT TTG TGG CTA AAC ACT	AGT CCC GTT TTG TCC TTA CGA
MIC10	GAT GCG GTC GTG AAG ATA GGT	CCA GAA CCG AAG GCT AAT GG
MIC60 (IMMT)	CGA TTC AGT CGG GTC CAC TAA	AGC TGG AGT ATC TCC CTT TTG T
OPA1	TGT GAG GTC TGC CAG TCT TTA	TGT CCT TAA TTG GGG TCG TTG
PAX6	TGG GCA GGT ATT ACG AGA CTG	ACT CCC GCT TAT ACT GGG CTA
SOX2	CCA TGC AGG TTG ACA CCG TTG	TCG GCA GAC TGA TTC AAA TAA TAC AG
TBR1	GCA GCA GCT ACC CAC ATT CA	AGG TTG TCA GTG GTC GAG ATA
TUBB3	GGC CAA GGG TCA CTA CAC G	GCA GTC GCA GTT TTC ACA CTC
VLCAD	ACA GAT CAG GTG TTC CCA TAC C	CTT GGC GGG ATC GTT CAC TT

Journal of Cell Science • Supplementary information

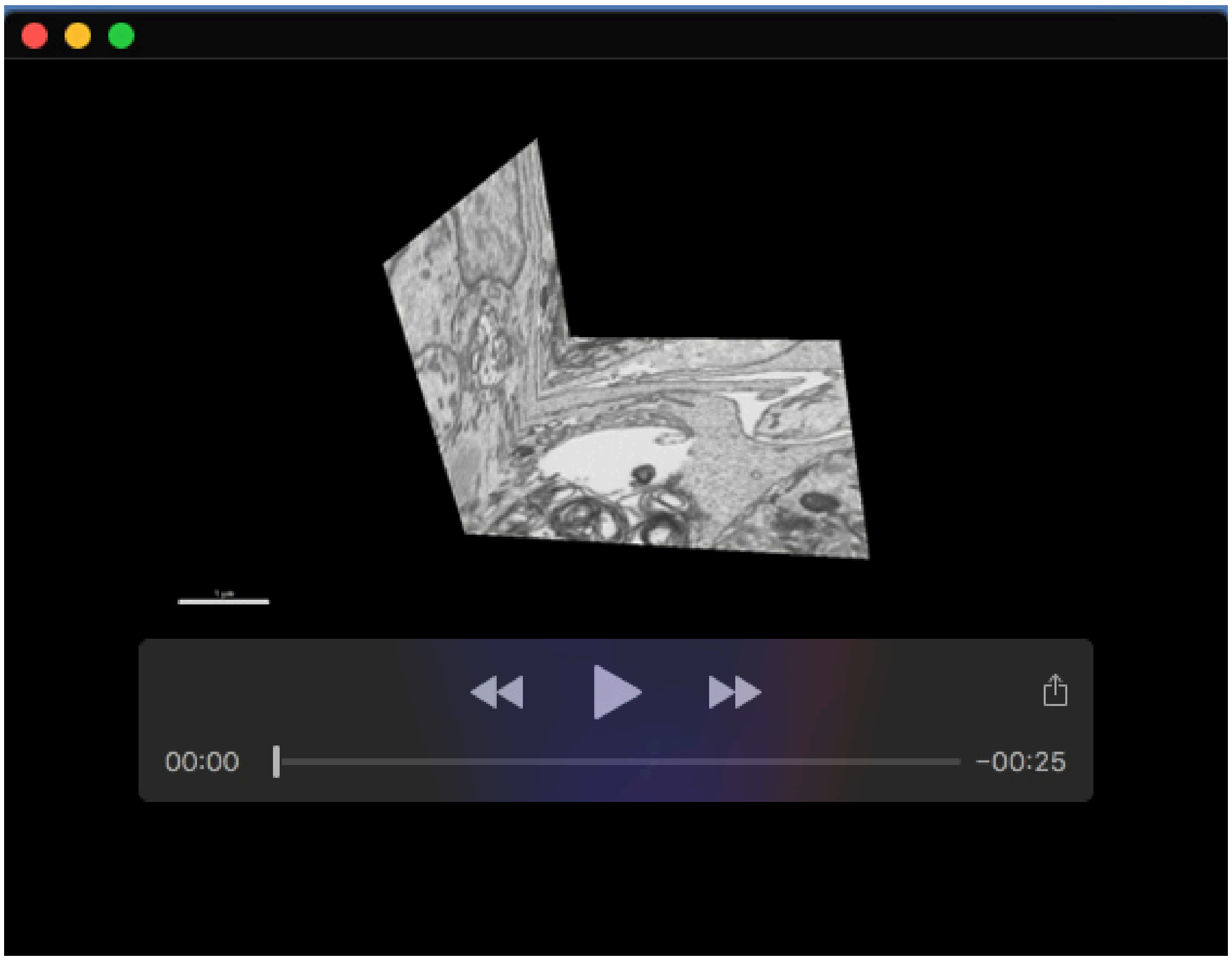

Movie 1. Volumetric reconstruction of mitochondrial cristae with FIB-SEM-acquired images of NPCs treated with DMSO. Animations were made from the FIB–SEM cropped images at 50 frames per second and 1000 frames total. Scale bars = 1μm.

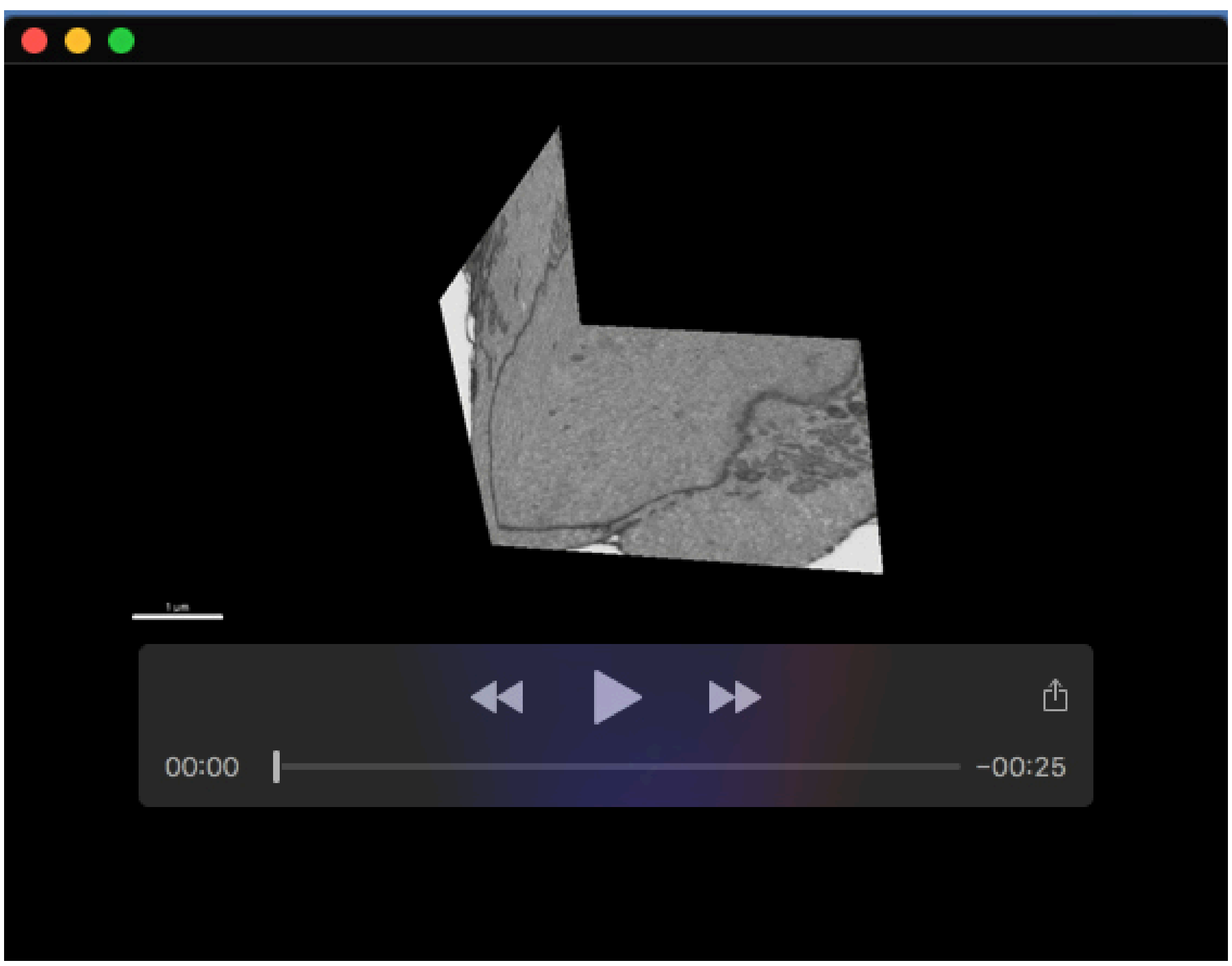

Movie 2. Volumetric reconstruction of mitochondrial cristae with FIB-SEM-acquired images of NPCs treated with S63845 (MCL-1 inhibitor). Animations were made from the FIB–SEM cropped images at 50 frames per second and 1000 frames total. Scale bars = 1μm.

Journal of Cell Science • Supplementary information
