# Supplementary material for: Pharmacological inhibition of MCL-1 disrupts mitochondrial cristae and depletes the human neural progenitor cell pool
Source: J Cell Sci. 2026 Oct 15;139(19):jcs265172. doi: 10.1242/jcs.265172 (PMC13741951; doi:10.1242/jcs.265172)
Supplement: Supplementary file 2 [file joces-139-265172_review_history.pdf]

Pharmacological inhibition of MCL-1 disrupts mitochondrial cristae and depletes the human neural progenitor cell pool

Marina R. Hanna, James Costanzo, Madison Yarbrough, Melanie Gil, Matthew Murrow and Vivian Gama

DOI: 10.1242/jcs.265172

Editor: Richa Rikhy

Review timeline

Submission to Review Commons:	22 December 2025
Submission to Journal of Cell Science:	06 July 2026
Editorial decision:	28 July 2026
First revision received:	01 August 2026
Accepted:	02 September 2026

Reviewer 1

Evidence, reproducibility and clarity

Summary: This study claims that beyond its canonical anti-apoptotic function, MCL-1 has essential non-apoptotic roles in human neurodevelopment. Pharmacologic inhibition of MCL-1 in human neural stem cells disrupts mitochondrial inner membrane architecture by destabilizing cristae and the OPA1-MICOS complex, leading to swollen mitochondria with disorganized cristae. These structural defects impair fatty acid oxidation and lipid droplet homeostasis, linking cristae integrity to metabolic competence. Independently of apoptosis or proliferation, MCL-1 inhibition selectively depletes intermediate neural progenitors, indicating a direct role in lineage progression. Overall, the work positions MCL-1 as a key regulator of mitochondrial structure-metabolism coupling that instructs neural progenitor identity and human neurogenesis.

Overall: The study does a good job of using (in most assays) caspase inhibition (e.g., QVD treatment) to block apoptotic responses induced by MCL-1 inhibition. As a result, many of the phenotypes caused by inhibition are likely to be independent of caspase activation. As a result, this manuscript would be of interest to researchers that study the topics of the BCL-2 family and cell death signaling, mitochondrial bioenergetics and dynamics, neurodevelopment, and cellular metabolism. However, as currently presented the manuscript is only descriptive and lacks mechanistic insight.

Major Concerns:

1) The authors only use a single MCL-1 inhibitor and never use other non-targeting BH3- mimetics (such as venetoclax) as negative controls. This seems like a missed opportunity to demonstrate that the phenotypes observed are MCL-1 dependent.

2) There is no mechanism proposed in this study other than reliance upon QVD as not affecting the phenotypes. As submitted, the manuscript only can speculate that these phenotypes are due to non-apoptotic roles of MCL-1 inhibition. The authors have missed an opportunity to explore MCL-1's non-apoptotic functions directly.

Other concerns exist that weaken the impact of the study.

1. Figure 1 should include the fact that QVD inhibition (shown in Sup Fig 2) does not obviate the phenotype induced by pharmacological inhibition of MCL-1 on mitochondrial morphology.

© 2026. Published by The Company of Biologists under the terms of the Creative Commons Attribution License (https://creativecommons.org/licenses/by/4.0/).

2. Figure 2 would benefit from evidence that caspase inhibition does not repress the phenotype on mitochondrial cristae morphology (volume and area). Furthermore, the FIB- SEM data are very hard to appreciate as the size precludes visualization of individual mitochondria.

3. Figure 3 reports that MIC60 and OPA1 appear to be downregulated in response to MCL-1 inhibition, but these appear to be more significant only when QVD is added. Why would the phenotype be obscured in the non-QVD setting (Fig. 2B&C). How does MCL-1 inhibition lead to changes in MIC60/MICOS/OPA1? This seems quite preliminary at this point.

4. The loss of MIC60 and OPA1 should repress electron transport chain function, are such impacts observed in the cultured cells? This could be shown by assessing oxygen consumption, etc. Such data would enhance the authors' conclusion that MCL-1 inhibition leads to defects in mitochondrial physiology.

5. In Figure 4, the differences between transcripts (qPCR data) and protein (immunoblot) data are often confusing and not well explained. Why do the authors propose that mRNA expression is decreasing whereas the protein expression is increasing? Example CPT1. Furthermore, it is unclear what these data mean functionally? Is this reflective of enhanced lipid oxidation or simply a response to inhibition of fatty acid oxidation? Clarification of the impact of these findings is necessary.

6. The increase in lipid droplet number induced by MCL-1 inhibition has been previously documented, but it is unclear whether this increase is related to an inability to oxidize lipid (defective fatty acid oxidation) that leads to increases in the cellular abundance or whether this indicates that MCL-1 inhibition leads to enhanced storage. Do other inhibitors of fatty acid oxidation lead to similar increases in lipid droplet size and abundance? Does QVD inhibition affect this phenotype?

7. For Figure 6, while these data may be very meaningful, as presented they are very hard to appreciate. Insets that show the neuronal populations would help to convey the point that the differentiation is impacted. Also, are there other methods that could confirm these observations (qPCR to show changes in differentiation).

8. Figure 7 is also very hard to appreciate. What is the reader to see? Can these be quantified? It seems that QVD may be rescuing in this figure, does this suggest that MCL-1 inhibition might be inducing death. All of this needs to be quantified.

9. BCL-XL has also been implicated in affecting mitochondrial electron transport chain function (See PMID: 19255249, 21926988, 21987637). Can BCL-XL inhibitors affect any of the phenotypes associated here?

10. Please be carefully avoid using the term "MCL-1 loss", when talking about pharmacological inhibition. Only genetic ablation (e.g. knockout, silencing, etc.) should be termed loss.

Significance

The study advances in human cells the impacts of MCL-1 inhibition. They replicate many impacts previously observed in mouse systems and refine analyses to impacts on MICOS complex, lipid droplet storage, and neuronal differentiation. While these findings are important and would be well received by a wide audience, the study fails to provide almost any mechanistic insight into how these phenotypes are being induced. The only common theme is that blocking caspase activation in many assays fails to block the phenotype.

Reviewer 2

Evidence, reproducibility and clarity

Summary:

© 2026. Published by The Company of Biologists under the terms of the Creative Commons Attribution License (https://creativecommons.org/licenses/by/4.0/).

This manuscript by Hanna et al. investigates non-apoptotic roles of MCL-1 in human neural stem cells and connects MCL-1 inhibition to mitochondrial cristae formation and beta- oxidation. Connecting these roles to brain development, the authors also show a reduction in the number of progenitor cells upon MCL-1 inhibition, independently of caspase activity. Throughout their work, the authors make use of an impressive array of imaging techniques.While the methods used offer sufficient evidence to connect MCL-1 inhibition to cristae architecture, the mechanistic underpinnings of this effect remain unexplored.

Major comments:

- In Fig. 1B, the very same representative images are shown for both conditions (DMSO and S63845) at 48 hours.
- For Western Blot analysis, it looks like the authors only quantified the band density of their proteins of interest without considering varying levels of control protein (Actin) levels. Normalizing the protein levels to actin would account for any differences in loaded protein amounts (although a Ponceau staining might be preferable still to exclude this). This is especially relevant for Fig. 4E, where actin levels visibly differ between the conditions.
- The authors offer evidence that MCL-1 inhibition impedes proteolytic cleavage of OPA1-L into the OPA-1-S isoforms, yet do not explore the mechanism behind this. Since OPA1 is cleaved by both OMA1 and YME1L, determination of the levels of these proteases could help shed some light on the mechanism leading to cristae reorganization.
- Generally speaking, while the authors show all those effects (cristae defects, FAO dysfunction) upon MCL-1 inhibition, it would be interesting to see whether any of those effects can be rescued by blocking FA import e.g. through carnitine palmitoyl- transferase 1a (CPT1a) inhibition with etomoxir to understand if they are downstream of altered Fa supply. This could affect cristae morphology through altered Cardiolipin biogenesis.
- In line 262 the authors discuss that mitochondria lose metabolic function upon MCL-1 inhibition. This claim would require additional experiments. While the authors look at lipid droplet accumulation and FAO enzymes, there are many more aspects to mitochondrial metabolic function that should be investigated. While measuring the oxygen consumption rate via Seahorse might require additional resources (optional), measurements of ATP production, ROS generation or determination of the mitochondrial membrane potential should be feasible.
- While the authors "propose a model in which MCL-1 associates with MICOS", they do not offer direct scientific to support this hypothesis. Co-immunoprecipitation experiments or e.g. proximity ligation assays would better support the proposed model.
- While Fig. 7 shows representative images, quantification e.g. for the truncation of neuronal processes is missing.
- In lines 219f. the authors state that they "observed a significant downregulation of PAX6 and EOMES at 24 hours that was not rescued by QVD co-treatment". While there is still a trend towards a downregulation, there is no statistical significance anymore. In fact, PAX6 levels almost mirror those of SOX2 which is not described as "downregulated" by the authors. In order to be more consistent, I would suggest rephrasing this part, or at least reword it to be less absolute.
- Brinkmann et al. (2025) also investigated cristae structure upon MCL-1 deletion in vivo and found no effect when MCL-1 was replaced with other Bcl-2 family members. It would be interesting to combine MCL-1 inhibition with overexpression of MCL-1 versus BCL-XL to reconsolidate some of the discrepant findings.

Minor comments:

- In Supp. Fig. 1C the MCL-1 protein is shown both to run above 37kDa (upper panel) and below 37 kDa (lower panel). Could the authors please comment on why this is the case?
- In line 64 of the introduction the authors mention clinical trials yet do not give a citation for these trials making it hard to judge whether the content of these trials is actually related to the brain.
- MCL-1 as well as ACSL-1 are sometimes written without the hyphen both in the text and figures.
- Lines 92-94 and 106-108 essentially highlight the same existing knowledge gap. Maybe the content of these two paragraphs could be combined in order to avoid repetition.

© 2026. Published by The Company of Biologists under the terms of the Creative Commons Attribution License (https://creativecommons.org/licenses/by/4.0/).

- In Fig. 1A, the authors provide a schematic for their experimental design. While the figure legend is very thorough, some of this information (like the days of collection) could also be included in the figure itself. The same is true for schematics in the following figures.
- Fig. 2A includes a typo (analyze) but would maybe also be more suitable for the supplement figures or could even be combined with Fig. 1A as not much new content is added.
- Regarding statistical analysis, could the authors please comment on why they did not consider one-sample t-tests suitable for the cases where control values were set at 1 (e.g. Fig. 4B, C for the relative expression).
- In lines 247f. the authors state that "inhibition of MCL-1 leads to [...] and disassembly of the MICOS complex as well as OPA1". This sounds like OPA1 is still cleaved upon MCL-1, which is not at all what the authors showed and further discuss. Rewording of the sentence would help in avoiding any misunderstandings.
- In lines 210f. the authors state that "quantitative imaging increased the average and maximum volume of lipid droplets". While there is definitely a trend towards an increase for the maximum volume, the increase is in fact not statistically significant. This should be reflected in the wording.
- In Fig. 6 the overlap between TBR2 and PAX6 is hard to judge when printed out. Including a zoom-in may make it easier to judge.
- In Fig. 7 the color-coding is listed in the figure legend but is missing from the figure itself. If the authors could include this, as they did for the other figures, it would further improve this figure.
- Line 238 should reference Fig. 7A, as Fig 7B does not exist.
- In the figure legends the authors state that biological replicates were used. Were technical replicates also performed?

Significance

The authors make use of a wide array of imaging techniques to further elucidate non- apoptotic roles of MCL-1. The study has the potential to offer new insights into mitochondrial biology on the level of basic research rather than translational. While the methods used offer sufficient evidence to connect MCL-1 inhibition to cristae architecture, the mechanistic underpinnings of this effect remain unexplored. Nevertheless, the study offers additional knowledge on the role of MCL-1 in human neural stem cells, whereas previous research mostly focused on cardiomyocytes or cancer cells.

Reviewer 3

Evidence, reproducibility and clarity

Summary:

In this study, Gama et al. describe a non-canonical role for the anti-apoptotic protein Myeloid Cell Leukemia-1 (MCL1) in mitochondrial cristae organization and suggest a role of MCL1 in regulating metabolism and neuronal differentiation. Using fluorescence microscopy imaging and electron microscopy, the authors show changes to mitochondrial morphology upon treatment with MCL1 inhibitor S63845. MCL1 inhibition results in altered protein and transcript levels of some key proteins involved in mitochondrial cristae organization and fatty acid metabolism. While some of the findings are interesting and indeed point towards a non- canonical role of MCL1, several key conclusions of the authors are not sufficiently supported by the data shown in the manuscript.

Major comments:

1. The authors try to disentangle the apoptotic and non-apoptotic role of MCL1 through addition of a caspase inhibitor. However, I am not convinced that phenotypes found under the addition of caspase inhibitor are necessarily caused by non-canonical functions independent of apoptosis. It could also be that the observed changes happen upstream of caspase activation. In addition, many of the described finding, such as CPT1 expression changes, only happen in the presence of the caspase inhibitor. If one follows the logic of the authors, changes associated by non-canonical MCL1 functions should happen under MCL1 inhibition and caspase inhibition, but not with MCL1

© 2026. Published by The Company of Biologists under the terms of the Creative Commons Attribution License (https://creativecommons.org/licenses/by/4.0/).

inhibition only.

2. The authors show no data on the viability of the cells in response to the MCL1 inhibitor. To exclude secondary effects of the inhibitor, at least some of the results should be validated with an MCL1 knock down.

3. In Figure 1, the authors show immunofluorescence data of mitochondria and nucleus staining and conclude that MCL1 inhibition alters mitochondrial morphology. Based on the images shown in Fig. 1, I do not think that individual mitochondria can be segmentd to measure their volume and length. In addition, some metrics such as mitochondrial content are not explained in the text or methods.

4. In Fig. 2 B-D, the authors show TEM and FIB-SEM imaging to demonstrate alterations in the cristae architecture upon treatment with MCL1 inhibitor. However, based on the images shown, it looks that cristae area and density is reduced under S63845 treatment in TEM images, while the FIB-SEM data come to the opposite conclusion. In addition, the quantification of cristae volume quantified as cristae volume in percentage is unclear to me.

5. The change in CPT1/2 protein levels (Fig. 4) is interesting but does not directly proof that fatty acid oxidation is altered, as concluded by the authors. For this, the authors would need to directly measure fatty acid oxidation for example using Seahorse or metabolic tracing experiments. Also, to prove that the MCL1 inhibition affects neural differentiation through fatty acid oxidation, a rescue experiment should be performed through CPT1 overexpression.

6. In Figure 6, the authors show decreased intermediate progenitor cells after MCL1 inhibition by immunofluorescence staining. I am not convinced that this can be concluded from the data shown, since the concentration of intermediate progenitor cells is very close to the noise levels. Since the MCL1 treated cells look much less sparse, I don't think the percentages can be compared (total counts are between 2-20). Although this data might give some indication that differentiation could be impaired, the measured effect could be very well due to lower viability of th
e cells. The authors need to control for this or come up with a different method for measuring differentiation.

7. Figure 7 is missing quantification

Significance

General assessment: The manuscript reports an interesting finding, which suggest a non-canonical role of MCL1 in mitochondrial remodeling, regulation of fatty acid oxidation and neuronal fate. While this finding would be highly interesting and relevant, the presented data do not sufficiently support this conclusion. Further experiments would have to be performed to proof causality.

Advance: Should the authors manage to proof their hypothesis by additional experiments, this would indeed advance the field on mitochondrial remodeling and its effect on neuronal differentiation by identifying a novel molecular player.

Audience: mitochondrial biology, cell biology, developmental neuroscience

Own expertise: mitochondrial biology, cell biology, advanced imaging techniques

---

Author response to reviewers' comments

Manuscript number: Refereed Preprint RC-2025-03357
Corresponding author(s):

© 2026. Published by The Company of Biologists under the terms of the Creative Commons Attribution License (https://creativecommons.org/licenses/by/4.0/).

Vivian, Gama

[The "revision plan" should delineate the revisions that authors intend to carry out in response to the points raised by the referees. It also provides the authors with the opportunity to explain their view of the paper and of the referee reports.

The document is important for the editors of affiliate journals when they make a first decision on the transferred manuscript. It will also be useful to readers of the reprint and help them to obtain a balanced view of the paper.

If you wish to submit a full revision, please use our "Full Revision" template. ***It is important to use the appropriate template to clearly inform the editors of your intentions****.]*

1. General Statements [optional]

This section is optional. Insert here any general statements you wish to make about the goal of the study or about the reviews.

We thank all reviewers for the valuable feedback and critical insight on our study. We acknowledge the concern that the manuscript, in its initial form, appeared descriptive and did not provide the mechanistic insight inferred from the current data. In the revised manuscript, we will (i) more clearly delineate what mechanistic inferences can be drawn from the existing data, (ii) expand our discussion of the caspase-independent mechanisms, and (iii) incorporate additional experiments/analyses aimed at identifying downstream effectors that mediate the observed phenotypes.

1. Specifically, to address questions about mechanistic insight, we generated stable ACSL1:HaloTag expressing hESCs. Presented as **new Figure 6** in the revised manuscript. ACSL1 is a critical enzyme that catalyzes the first step of faPy acid oxidation at the outer mitochondrial membrane. Our previous analysis and work from the Opferman lab demonstrated that ACSL1 contains a BH3-like domain. Thus, we examined the effects of MCL-1 inhibition with S63845 on the mitochondrial localization of this enzyme. Our findings pinpoint that MCL-1 inhibition is causing the displacement of ACSL1 from the mitochondria (**new Figure 6** in the revised manuscript). Our interpretations of the effects of MCL-1 inhibition are 2-fold: 1) as we show in our data, MCL-1 inhibition causes disruption of the mitochondrial cristae, altering the microenvironment for faPy acid oxidation, and 2) as seen in cancer cells, the MCL-1 inhibitor may also displace ACSL1 from the mitochondria. In the new version of the manuscript, we will focus on these 2 mechanisms as mechanistic outcomes of MCL-1 inhibition.

2. We have now included data on the effects of MCL-1 inhibition on the respiratory capacity of human neural progenitor cells (NPCs), as suggested by all reviewers. These data are included in the **new Supplementary Figure 4** of the revised manuscript.

3. As requested by all reviewers, we have now performed quantification on the early-born neurons treated with the MCL-1 inhibitor, shown as **new Figure 8B**.

4. We have included data of cells treated with Perhexiline (CPT1/2 inhibitor), and Etomoxir (CPT1a inhibitor) (**Figure 1 for reviewers**).

All referenced figure numbers correspond to the manuscript document. Modifications to original figures have been specified "**new Figure X** in the revised manuscript." We have included four figures available only to reviewers, which are referred to as "**Figure X for reviewers.**"

2. Description of the planned revisions

Insert here a point-by-point reply that explains what revisions, additional experimentations and analyses are planned to address the points raised by the referees.

© 2026. Published by The Company of Biologists under the terms of the Creative Commons Attribution License (https://creativecommons.org/licenses/by/4.0/).

Reviewer #1 (Evidence, reproducibility and clarity (Required)):

Summary: This study claims that beyond its canonical anti-apoptotic function, MCL-1 has essential non- apoptotic roles in human neurodevelopment. Pharmacologic inhibition of MCL-1 in human neural stem cells disrupts mitochondrial inner membrane architecture by destabilizing cristae and the OPA1-MICOS complex, leading to swollen mitochondria with disorganized cristae. These structural defects impair faHy acid oxidation and lipid droplet homeostasis, linking cristae integrity to metabolic competence. Independently of apoptosis or proliferation, MCL-1 inhibition selectively depletes intermediate neural progenitors, indicating a direct role in lineage progression. Overall, the work positions MCL-1 as a key regulator of mitochondrial structure-metabolism coupling that instructs neural progenitor identity and human neurogenesis.

Overall: The study does a good job of using (in most assays) caspase inhibition (e.g., QVD treatment) to block apoptotic responses induced by MCL-1 inhibition. As a result, many of the phenotypes caused by inhibition are likely to be independent of caspase activation. As a result, this manuscript would be of interest to researchers that study the topics of the BCL-2 family and cell death signaling, mitochondrial bioenergetics and dynamics, neurodevelopment, and cellular metabolism. However, as currently presented the manuscript is only descriptive and lacks mechanistic insight.

We thank Reviewer 1 for the insightful evaluation of our work. We are encouraged that the reviewer finds the study relevant to investigators in the fields of BCL-2 family biology, mitochondrial dynamics and bioenergetics, neurodevelopment, and cellular metabolism. We also thank the reviewer for pointing out the need to increase the mechanistic insight of our findings. As mentioned above, in the revised manuscript, we provide evidence of a mechanistic link between MCL-1 and ACSL1.

Major Concerns:

1) The authors only use a single MCL-1 inhibitor and never use other non-targeting BH3-mimetics (such as venetoclax) as negative controls. This seems like a missed opportunity to demonstrate that the phenotypes observed are MCL-1 dependent.

We thank the reviewer for this suggestion. While we agree that venetoclax treatment would provide a useful comparator, this experiment fell outside the scope of the current study, as our efforts were directed towards prioritizing experiments more central to the core findings; such as, the previously reported interaction between ACSL-1’s BH3-like domain and MCL-1’s BH3-binding domain. We have, however, added this to the discussion as a valuable avenue for future investigation.

2) There is no mechanism proposed in this study other than reliance upon QVD as not affecting the phenotypes. As submiHed, the manuscript only can speculate that these phenotypes are due to non-apoptotic roles of MCL-1 inhibition. The authors have missed an opportunity to explore MCL-1's non-apoptotic functions directly.

We appreciate Reviewer 1’s comment. In the new version of the manuscript, we propose that MCL-1 is acting in 2 ways: 1) as demonstrated by our data, MCL-1 inhibition causes disruption of the mitochondrial cristae, consequently altering the microenvironment for faPy acid oxidation, and 2) as seen in cancer cells, MCL-1 inhibitors may also displace ACSL1 from the mitochondria.

Since receiving the initial reviews, we have focused on testing the laPer possibility, since the accumulation of lipids that we observed was also seen in cancer cells (PMID: 38ti03284). We generated stable ACSL1:HaloTag expressing hESCs to investigate the hypothesis that MCL-1 interacts with ACSL1 to aid in the localization of the enzyme at the outer mitochondrial membrane (OMM). Our findings, included in the new version of the manuscript, provide compelling evidence that ACSL1 is displaced from the mitochondria by MCL-1 inhibition with the BH-3 mimetic, S6384ti, in NPCs (**new Figure 6** in the revised manuscript).

© 2026. Published by The Company of Biologists under the terms of the Creative Commons Attribution License (https://creativecommons.org/licenses/by/4.0/).

Other concerns exist that weaken the impact of the study.

1. Figure 1 should include the fact that QVD inhibition (shown in Sup Fig 2) does not obviate the phenotype induced by pharmacological inhibition of MCL-1 on mitochondrial morphology.

We would like to clarify that QVD does prevent the phenotypes induced by MCL-1 inhibition on mitochondrial morphology. In Fig1B, we report an increase in volume and surface area at 24h and 48h along with a decrease in mitochondrial content at 48h when NPCs were treated with S63845 only. However, NPCs co-treated with QVD in Supp Fig 2B did not exhibit any significant morphological phenotypes on average or at min/max values. Reviewer 1 may be referring to Fig 1B's corresponding min/max values presented in Supplementary Figure 2A where we reported an increase in **max** volume.

**Figure #**	**Volume**	**Surface Area**
**Fig 1B** (S63845 **only, avg values)**	*Increase (avg vol)*	*increase (avg)*
**Supp Fig 2B** (S63845 **+QVD)**	no change	no change
**Supp Fig 2A** (S63845 **only, max/min values)**	*increase (max vol)*	no change (max)

For clarity, we will move Supplementary Figure 2A into Supplementary Figure 1.

It is important to clarify that we have reevaluated our initial findings. In our first submission, the data were separated by both treatment timepoint (24h or 48h) and condition (-/+QVD), however, we recognize that in order to soundly claim whether or not QVD obviates a phenotype, a multiple comparison analysis was necessary. In the revised manuscript, data are now organized by timepoint (24h or 48h). We analyzed measurements using an ordinary two-way ANOVA with multiple comparison to statistically detect differences between S63845-only and S63845+QVD conditions at either timepoint. **New Figure 1** in the revised manuscript presents new insights and complexities in the relationship between S6384ti and QVD where some phenotypes (e.g., at 24h average surface area and volume) are rescued by QVD while others (e.g., at 48h average surface area and volume, maximum surface area and equatorial diameter) persist in the presence of QVD co-treatment. This suggests that MCL-1 is needed for long-term maintenance of mitochondrial morphology, functioning independently of its antiapoptotic role in caspase-initiated, apoptosis-independent conditions.

2. Figure 2 would benefit from evidence that caspase inhibition does not repress the phenotype on mitochondrial cristae morphology (volume and area). Furthermore, the FIB-SEM data are very hard to appreciate as the size precludes visualization of individual mitochondria.

While we included the visualization of the segmented mitochondria and cristae (Figure 2C), as well as snapshots through the z-stack for segmented cristae only (Figure 2E) and segmented mitochondria separately (Supp Figure 3A) in the original manuscript, we are also now aPaching the FIB-SEM 3D reconstruction videos (**new Supplementary Videos 1-2** in the revised manuscript) (1. Mitochondria and cristae, 2. Cristae only, 3. Mitochondria only) for ease of visualization purposes.

3. Figure 3 reports that MIC60 and OPA1 appear to be downregulated in response to MCL-1 inhibition, but these appear to be more significant only when QVD is added. Why would the phenotype be obscured in the non-QVD se\ng (Fig. 2B&C). How does MCL-1 inhibition lead to changes in MIC60/MICOS/OPA1? This seems quite preliminary at this point.

© 2026. Published by The Company of Biologists under the terms of the Creative Commons Attribution License (https://creativecommons.org/licenses/by/4.0/).

We did not report a downregulation of *MIC60* or *OPA1* mRNA transcripts in the initial submission. In Figures 3B and 3C, however, we reported decreased protein levels of short-form OPA1 and MIC10 only, not MIC60. In the cases where phenotypes are more significant when QVD is added, we infer that the addition of QVD allows us to capture a snapshot of NPC populations that are more vulnerable to MCL-1 inhibition. Although these cell types may not survive the absence of MCL-1 function, we cannot aPribute it to the protein's anti-apoptotic canonical function by default, since it is possible that they do not survive mass dismantling of cristae architecture and/or disruption in FAO. We interpret this complex phenotype to be aPributed to either heavily rely on MCL-1's anti- apoptotic properties or noncanonical properties; such as, FAO oxidation. This is most apparent with our discovery in the depletion of the intermediate progenitor cell (IPC) pool upon S63845 -/+ QVD treatments at both timepoints. We argue that our data with QVD shows that the cell death function of MCL-1 (i.e., inhibiting cell death effectors from initiating the caspase cascade) is not the main trigger of the phenotypes we report (cristae dysregulation and faPy acid oxidation disruption), however, cells without a functional cristae and/or defects in FAO, may not be able to survive long-term. Thus, QVD treatment preserves these cells that may not survive the dismantling of such an essential structure. To confirm this, we have performed immunofluorescence of cleaved caspase 3 (**Figure 2 for reviewers**). These results show that, indeed, MCL-1 inhibition at the time points of our study does not result in increased activation of Caspase-3. We reported similar results of MCL-1 inhibition in oligodendrocyte precursor cells (Gil and Hanna et al., Glia, 202ti, PMID: 41420072).

To more directly address the question of how MCL-1 inhibition may lead to changes in cristae-shaping proteins, we recognize our data, as it stands, is somewhat preliminary. It has been previously reported and widely recognized that a truncated form of MCL-1 localizes to the inner mitochondrial membrane (IMM) without a known function. In this manuscript, we report for the first time that MCL-1 inhibition leads to disorganized cristae architecture. Taken together, these findings point towards a new role for MCL-1 in maintaining cristae folding, potentially through modulation or support of cristae-shaping proteins. Here, we observe decreased levels of short-form OPA1 and a core MICOS subcomplex protein, MIC10. We plan to further explore this by performing immunoprecipitation and proximity ligation assays between MCL-1 and cristae-shaping proteins.

4. The loss of MIC60 and OPA1 should repress electron transport chain function, are such impacts observed in the cultured cells? This could be shown by assessing oxygen consumption, etc. Such data would enhance the authors' conclusion that MCL-1 inhibition leads to defects in mitochondrial physiology.

We completely agree with this comment by Reviewer 1. We have included an assessment of mitochondrial oxygen consumption rate, using the Seahorse XF Mito Stress Test on NPCs treated with S6384ti -/+ QVD at both timepoints. These data are now presented as **new Supplementary Figure 4** in the revised manuscript.

ti. In Figure 4, the differences between transcripts (qPCR data) and protein (immunoblot) data are oden confusing and not well explained. Why do the authors propose that mRNA expression is decreasing whereas the protein expression is increasing? Example CPT1. Furthermore, it is unclear what these data mean functionally? Is this reflective of enhanced lipid oxidation or simply a response to inhibition of faHy acid oxidation? Clarification of the impact of these findings is necessary.

We have now made sure to clarify our interpretation of these findings, both in the Results and the Discussion sections of the revised manuscript. It is important to bring aPention to the phenomenon of opposing mRNA and protein levels. This is most evident with *MCL-1* mRNA and MCL-1 protein, where there is an increase in protein levels despite mRNA downregulation. Accumulation of MCL-1 protein has been previously reported as an effect of MCL-1 inhibition and is aPributed to post-translational modifications to the protein that increase stability and prevent degradation. This is a possible explanation for other similar observations of other proteins, such as, CPT1a.

To directly address the question regarding the RT-qPCR and immunoblot findings at the mRNA and protein levels, respectively, we revisited our approach to analysis with the intention to ensure proper analysis of these semi- quantitative approaches prior to making inferences about our

© 2026. Published by The Company of Biologists under the terms of the Creative Commons Attribution License (https://creativecommons.org/licenses/by/4.0/).

observations. We collaborated with a bioinformatician to design a model for our study using a published python package that is more suited and bePer fit to interpret RT-qPCR data than the traditional delta-delta CT fold-change (see **new methods secMon** in revised manuscript). We chose to still graph data using $2^{(-\Delta\Delta CT)}$ as it is suitable for visual representation, however, this approach removes variability from the control (DMSO) thus eliminating the equal variance assumption required for ANOVA and rendering such statistical tests unreliable. The analysis of the data, however, was conducted using ANCOVA as outlined in the revised methods section. Results are included in **new Figure 4** and **new Supplementary Figure ti** in the revised manuscript.

Reviewer 1's comment was also relevant to the immunoblot data presented in the study. Immunoblot data are also relative but with higher blot-to-blot variability. To address this, we rescaled the data while maintaining the relative variance of the points. Since immunoblot pixel density varies significantly across biological replicates due to factors independent of treatment (e.g. exposure time, antibody affinity), we controlled for these effects by rescaling pixel density measurements such that the average pixel density across treatments for each biological replicate was equal. This allows for more reliable ANOVA testing and bePer interpretation of immunoblot observations. These results are in **new Figure 3** and **new Supplementary Figure 3** in the revised manuscript.

6. The increase in lipid droplet number induced by MCL-1 inhibition has been previously documented, but it is unclear whether this increase is related to an inability to oxidize lipid (defective faHy acid oxidation) that leads to increases in the cellular abundance or whether this indicates that MCL-1 inhibition leads to enhanced storage. Do other inhibitors of faHy acid oxidation lead to similar increases in lipid droplet size and abundance? Does QVD inhibition affect this phenotype?

This is a great point raised by Reviewer 1, and one we have also considered. We conducted an experiment using C16 BODIPY to address this point (**Figure 3 for Reviewers**). We observed no changes in C16 lipid droplet accumulation in count, volume, or surface area when cells were treated with S6384ti for 24 hours total with or without a starvation period during the last 6 hours of treatment. However, we observed significant pan-lipid droplet accumulation in the same conditions. This contrast suggests that FAO of exogenous long chain faPy acids is not reliant on MCL-1. This contrasting finding does not discount the requirement of MCL-1 for other FAO processes, especially given the major limitation of how much C16 BODIPY (fluorescent palmitate) can be administered to the cells (10μM) which was 10-fold less than what we exogenously supplied to the cells for the pan-BODIPY experiment (100μM, see Figure ti). It is possible that this small dose was insufficient to detect lipid droplet accumulation.

We have now also included experiments with etomoxir and perhexiline to assess their effects on TBR2/PAX6 (see **Figure 1 for reviewers**). The results indicate that inhibiting the FAO pathway does not fully mimic the effects of S6384ti on TBR2. However, we show that S6384ti displaces ACSL1 from the mitochondria (**new Figure 6** in the revised manuscript**)**, a step that is upstream of CPT1 and 2. We suggest a model in which the coordinated non- apoptotic function of MCL-1 at the outer mitochondrial membrane promotes ACSL1 activity and, at the inner mitochondrial membrane, regulates mitochondrial cristae morphology. While our data point to this model, we are limited by the tools to investigate it further, but it will be a great direction for future experiments.

7. For Figure 6, while these data may be very meaningful, as presented they are very hard to appreciate. Insets that show the neuronal populations would help to convey the point that the differentiation is impacted. Also, are there other methods that could confirm these observations (qPCR to show changes in differentiation).

We agree with Reviewer 1. In the new version of the manuscript, we included panels that zoom into the cell populations we quantified (**new Figure 7** in the revised manuscript**)**. Additionally, we have included *TBR1* and *TUBB3* to the qPCR panel in **new Supplementary Figure ti** in the revised manuscript.

8. Figure 7 is also very hard to appreciate. What is the reader to see? Can these be quantified? It seems that QVD may be rescuing in this figure, does this suggest that MCL-1

© 2026. Published by The Company of Biologists under the terms of the Creative Commons Attribution License (https://creativecommons.org/licenses/by/4.0/).

inhibition might be inducing death. All of this needs to be quantified.

We now provide quantification of BIII-tubulin branching in the **new Figure 8** of the revised manuscript.

9. BCL-XL has also been implicated in affecting mitochondrial electron transport chain function (See PMID: 192titi249, 21926988, 21987637). Can BCL-XL inhibitors affect any of the phenotypes associated here?

We thank the reviewer for highlighting this interesting aspect of BCL-XL biology. While we agree that BCL-XL's role in mitochondrial electron transport chain function is relevant to the field, investigating it falls outside the scope of this study, which focuses on the clinical relevance of pharmacological MCL-1 inhibition with BH3 mimetics. We have noted this as a potential avenue for future investigation in the revised discussion.

10. Please be carefully avoid using the term "MCL-1 loss", when talking about pharmacological inhibition. Only genetic ablation (e.g. knockout, silencing, etc.) should be termed loss.

We appreciate Reviewer 1 for pointing this out. We have now removed the reference to MCL-1 loss in line 199.

Reviewer #1 (Significance (Required)):

The study advances in human cells the impacts of MCL-1 inhibition. They replicate many impacts previously observed in mouse systems and refine analyses to impacts on MICOS complex, lipid droplet storage, and neuronal differentiation. While these findings are important and would be well received by a wide audience, the study fails to provide almost any mechanistic insight into how these phenotypes are being induced. The only common theme is that blocking caspase activation in many assays fails to block the phenotype.

Reviewer #2 (Evidence, reproducibility and clarity (Required)):

Summary:
This manuscript by Hanna et al. investigates non-apoptotic roles of MCL-1 in human neural stem cells and connects MCL-1 inhibition to mitochondrial cristae formation and beta-oxidation. Connecting these roles to brain development, the authors also show a reduction in the number of progenitor cells upon MCL-1 inhibition, independently of caspase activity. Throughout their work, the authors make use of an impressive array of imaging techniques. While the methods used offer sufficient evidence to connect MCL-1 inhibition to cristae architecture, the mechanistic underpinnings of this effect remain unexplored.

We thank Reviewer 2 for the thoughtful and positive assessment of our manuscript. We appreciate the reviewer's recognition that our study reveals non-apoptotic roles of MCL-1 in human neural stem cells. We are also grateful for the acknowledgment of the imaging approaches employed, which enabled us to link MCL-1 function to cristae architecture using multiple complementary techniques. We acknowledge the reviewer's point that the mechanistic basis of MCL-1's influence on cristae structure remains insufficiently defined. In the revised manuscript, we will clarify the limitations of the current data, expand our discussion of potential mechanisms, and incorporate additional analyses to identify downstream effectors that mediate these structural and metabolic changes.

Major comments:

- In Fig. 1B, the very same representative images are shown for both conditions (DMSO and S63845) at 48 hours.

We deeply appreciate Reviewer 2 for catching this unintentional duplication that occurred during figure preparation. We have now corrected this issue.

© 2026. Published by The Company of Biologists under the terms of the Creative Commons Attribution License (https://creativecommons.org/licenses/by/4.0/).

- For Western Blot analysis, it looks like the authors only quantified the band density of their proteins of interest without considering varying levels of control protein (Actin) levels. Normalizing the protein levels to actin would account for any differences in loaded protein amounts (although a Ponceau staining might be preferable still to exclude this). This is especially relevant for Fig. 4E, where actin levels visibly differ between the conditions.

All WB quantifications were normalized to Actin (this detail is now added to the y-axis of all band density graphs and figure legends). We agree with the reviewer that there is inherent technical variability in immunobloqng. We more thoroughly address this issue by rescaling the pixel density data normalized to Actin (please see response to Reviewer 1, comment 5). By minimizing the gel-to-gel variability in our data, we were able to more reliably detect differences in samples using an ordinary two-way ANOVA with multiple comparison test. These results are in **new Figure 3** and **new Supplementary Figure 3** in the revised manuscript.

- The authors offer evidence that MCL-1 inhibition impedes proteolytic cleavage of OPA1-L into the OPA-1-S isoforms, yet do not explore the mechanism behind this. Since OPA1 is cleaved by both OMA1 and YME1L, determination of the levels of these proteases could help shed some light on the mechanism leading to cristae reorganization.

We thank Reviewer 2 for this interesting point. As our study is focused on the direct mechanisms by which MCL-1 inhibition alters FAO, we prioritized experiments central to this question. We agree, however, that the mechanism by which MCL-1 impedes proteolytic cleavage of OPA1L in this context is a compelling avenue for future investigation.

- Generally speaking, while the authors show all those effects (cristae defects, FAO dysfunction) upon MCL-1 inhibition, it would be interesting to see whether any of those effects can be rescued by blocking FA import e.g. through carnitine palmitoyl- transferase 1a (CPT1a) inhibition with etomoxir to understand if they are downstream of altered Fa supply. This could affect cristae morphology through altered Cardiolipin biogenesis.

This is an excellent point, which was also raised by reviewer 1. We have now included experiments using etomoxir and perhexiline to assess their effects on TBR2/PAX6 (**Figure 1 for Reviewers**). As mentioned above, the results indicate that inhibiting the FAO pathway does not fully mimic the effects of S63845 on TBR2. However, we show that S63845 displaces ACSL1 from the mitochondria, a step that is upstream of CPT1 and 2. We suggest a model in which the coordinated non-apoptotic function of MCL-1 at the outer mitochondrial membrane promotes ACSL1 activity and, in the inner mitochondrial membrane, regulates mitochondrial cristae morphology. While our data point to this model, we are limited by the tools to investigate it further, but it will be a great direction for future experiments. The suggestion of Reviewer 2 that the effects on FAO could impact cardiolipin biogenesis is a very exciting possibility. However, difficult to test with the tools available.

- In line 262 the authors discuss that mitochondria lose metabolic function upon MCL-1 inhibition. This claim would require additional experiments. While the authors look at lipid droplet accumulation and FAO enzymes, there are many more aspects to mitochondrial metabolic function that should be investigated. While measuring the oxygen consumption rate via Seahorse might require additional resources (optional), measurements of ATP production, ROS generation or determination of the mitochondrial membrane potential should be feasible.

We fully agree with Reviewer 2's comment, which was also raised by Reviewer 1. In our revision, we now include a complete assessment of the mitochondrial oxygen consumption rate of NPCs treated with S63845, measured using the Seahorse analyzer (mitochondrial stress test). These data are presented as **new Supplementary Figure 4** in the revised manuscript.

- While the authors "propose a model in which MCL-1 associates with MICOS", they do not offer direct scientific to support this hypothesis. Co-immunoprecipitation experiments or e.g. proximity ligation assays would beHer support the proposed model.

We agree with this statement. As a preliminary step, we performed proximity ligation assays and

© 2026. Published by The Company of Biologists under the terms of the Creative Commons Attribution License (https://creativecommons.org/licenses/by/4.0/).

immunoprecipitation analyses to test for this interaction (**see below and Figure 4 for reviewers**). The results indicate an interaction with MIC60, albeit very weak. The complexity of these analyses and their interpretation under endogenous conditions can make interpretation difficult.

NOTE: We have removed unpublished data that had been provided for the referees in confidence.

- While Fig. 7 shows representative images, quantification e.g. for the truncation of neuronal processes is missing.

We thank Reviewer 2 for this important point. We have now included quantification alongside the images provided (new **Figure 8** in the revised manuscript).

- In lines 219f. the authors state that they "observed a significant downregulation of PAX6 and EOMES at 24 hours that was not rescued by QVD co-treatment". While there is still a trend towards a downregulation, there is no statistical significance anymore. In fact, PAX6 levels almost mirror those of SOX2 which is not described as "downregulated" by the authors. In order to be more consistent, I would suggest rephrasing this part, or at least reword it to be less absolute.

In the new version, we clarified that while QVD rescued *PAX6* and *EOMES* transcript levels at 24h, it did not rescue *EOMES* at 48h (**new Supplementary Figure 5** in the revised manuscript). It is important to note that in our first submission, the data were separated by both treatment timepoint (24h or 48h) and condition (-/+QVD). We recognize, however, that in order to soundly claim whether QVD rescues a phenotype, a multiple comparison analysis was necessary. In the revised manuscript, data are now organized by timepoint (24h or 48h) and analyzed using ANCOVA to statistically detect differences between S63845-only and S63845+QVD conditions at either timepoint.

- Brinkmann et al. (2025) also investigated cristae structure upon MCL-1 deletion in vivo and found no effect when MCL-1 was replaced with other Bcl-2 family members. It would be interesting to combine MCL-1 inhibition with overexpression of MCL-1 versus BCL-XL to reconsolidate some of the discrepant findings.

While this is a great suggestion for future studies, there are some complications. Specifically, it is likely that the inhibitor may also target the overexpressed MCL-1 and thus, a mutant form is needed.

To address this, we generated a Flag-tagged MCL-1 construct with a mutated BH3 domain, previously described by Kotschy et al. Nature 2016. We validated the construct in HeLa cells, but unfortunately, the mutant protein appears to be significantly less stable than the WT construct, complicating analysis of this experiment.

NOTE: We have removed unpublished data that had been provided for the referees in confidence.

Minor comments:

- In Supp. Fig. 1C the MCL-1 protein is shown both to run above 37kDa (upper panel) and below 37 kDa (lower panel). Could the authors please comment on why this is the case?

The observed variation is caused by dris in the gel during SDS-PAGE electrophoresis. In Fig 1C, the protein ladder is on the edge of the gel, whereas in Fig 1E, the protein ladder is in the middle of the gel, and the last sample is on the edge thus exhibiting some edge dris.

- In line 64 of the introduction the authors mention clinical trials yet do not give a citation for these trials making it hard to judge whether the content of these trials is actually related to the brain.

This information is anecdotal, based on an Amgen press release.

© 2026. Published by The Company of Biologists under the terms of the Creative Commons Attribution License (https://creativecommons.org/licenses/by/4.0/).

- MCL-1 as well as ACSL-1 are sometimes wriHen without the hyphen both in the text and figures.

We thank Reviewer 2 for this comment. We have carefully checked the manuscript before submission to correct this inconsistency.

- Lines 92-94 and 106-108 essentially highlight the same existing knowledge gap. Maybe the content of these two paragraphs could be combined in order to avoid repetition.

We thank Reviewer 2 for this suggestion. We have accounted for this in the new version of the manuscript to avoid repetition.

- In Fig. 1A, the authors provide a schematic for their experimental design. While the figure legend is very thorough, some of this information (like the days of collection) could also be included in the figure itself. The same is true for schematics in the following figures.

We agree with this and have incorporated the suggestion in the schematics of the revised manuscript (**Figures 1A, 2A, 3A, tia**).

- Fig. 2A includes a typo (analyze) but would maybe also be more suitable for the supplement figures or could even be combined with Fig. 1A as not much new content is added.

We thank the reviewer for catching these typos. We have incorporated these changes into the revised version of the manuscript.

- Regarding statistical analysis, could the authors please comment on why they did not consider one-sample t- tests suitable for the cases where control values were set at 1 (e.g. Fig. 4B, C for the relative expression).

We thank the reviewer for this thoughtful comment. For comparisons involving multiple treatment groups, we have now used the ANCOVA framework as suggested by Hampton et al. This approach was selected to allow simultaneous comparison across all groups while accounting for the lack of variability in the control sample DMSO with ΔΔCT. We believe the revised statistical approach more appropriately addresses the important question raised by Reviewer 2. For more details, please see our response to Reviewer 1, comment 5, and the methods section in the revised manuscript.

- In lines 247f. the authors state that "inhibition of MCL-1 leads to [...] and disassembly of the MICOS complex as well as OPA1". This sounds like OPA1 is still cleaved upon MCL-1, which is not at all what the authors showed and further discuss. Rewording of the sentence would help in avoiding any misunderstandings.

We agree with this comment and have now reworded our interpretation: "These isoform-specific reductions suggest that MCL-1 inhibition disrupts the stoichiometric balance between fusion-competent long-form OPA1 (OPA1-L) and its short isoforms (OPA1-S1/2), potentially reflecting an impairment of OPA1-L proteolytic processing rather than enhanced cleavage activity…. Collectively, these findings identify MCL-1 as a critical upstream regulator of mitochondrial inner membrane organization, acting through parallel destabilization of OPA1 isoform homeostasis and MICOS complex integrity to maintain cristae architecture."

- In lines 210f. the authors state that "quantitative imaging increased the average and maximum volume of lipid droplets". While there is definitely a trend towards an increase for the maximum volume, the increase is in fact not statistically significant. This should be reflected in the wording.

We have reworded this to "Quantitative imaging analysis revealed a significant increase in average lipid droplet volume and a trending increase in maximum volume."

- In Fig. 6 the overlap between TBR2 and PAX6 is hard to judge when printed out. Including a

© 2026. Published by The Company of Biologists under the terms of the Creative Commons Attribution License (https://creativecommons.org/licenses/by/4.0/).

zoom-in may make it easier to judge.

We agree with Reviewer 2. We have included panels that zoom into the cell populations we quantified (**new Figure 7** in the revised manuscript).

- In Fig. 7 the color-coding is listed in the figure legend but is missing from the figure itself. If the authors could include this, as they did for the other figures, it would further improve this figure.

We agree. We have specified the channel color in the **new Figure 7**.

- Line 238 should reference Fig. 7A, as Fig 7B does not exist.

Thanks for catching this. It is corrected in the revised manuscript.

- In the figure legends the authors state that biological replicates were used. Were technical replicates also performed?

Yes, technical replicates were performed for RT-qPCR. This information is now included in the figure legend.

Reviewer #2 (Significance (Required)):

Significance

The authors make use of a wide array of imaging techniques to further elucidate non-apoptotic roles of MCL-1. The study has the potential to offer new insights into mitochondrial biology on the level of basic research rather than translational. While the methods used offer sufficient evidence to connect MCL-1 inhibition to cristae architecture, the mechanistic underpinnings of this effect remain unexplored. Nevertheless, the study offers additional knowledge on the role of MCL-1 in human neural stem cells, whereas previous research mostly focused on cardiomyocytes or cancer cells

Reviewer #3 (Evidence, reproducibility and clarity (Required)):

Summary:

In this study, Gama et al. describe a non-canonical role for the anti-apoptotic protein Myeloid Cell Leukemia-1 (MCL1) in mitochondrial cristae organization and suggest a role of MCL1 in regulating metabolism and neuronal differentiation. Using fluorescence microscopy imaging and electron microscopy, the authors show changes to mitochondrial morphology upon treatment with MCL1 inhibitor S63845. MCL1 inhibition results in altered protein and transcript levels of some key proteins involved in mitochondrial cristae organization and faHy acid metabolism. While some of the findings are interesting and indeed point towards a non-canonical role of MCL1, several key conclusions of the authors are not sufficiently supported by the data shown in the manuscript.

We thank Reviewer 3 for the careful evaluation of our manuscript. We appreciate the reviewer's recognition that our study identifies a potential non-canonical role for MCL-1 in mitochondrial cristae organization, metabolism, and neuronal differentiation. As with Reviews 1 and 2, we are encouraged that the reviewer finds these observations interesting and suggestive of previously unappreciated functions for MCL-1. We agree that stronger evidence is required to firmly link MCL-1 inhibition to specific changes in MICOS organization and metabolic regulation. In the revised manuscript, we (i) more clearly distinguish between observations and mechanistic inferences, (ii) temper conclusions where appropriate, and (iii) incorporate additional analyses and controls to bePer substantiate the proposed model.

Major comments:

© 2026. Published by The Company of Biologists under the terms of the Creative Commons Attribution License (https://creativecommons.org/licenses/by/4.0/).

1. The authors try to disentangle the apoptotic and non-apoptotic role of MCL1 through addition of a caspase inhibitor. However, I am not convinced that phenotypes found under the addition of caspase inhibitor are necessarily caused by non-canonical functions independent of apoptosis. It could also be that the observed changes happen upstream of caspase activation. In addition, many of the described finding, such as CPT1 expression changes, only happen in the presence of the caspase inhibitor. If one follows the logic of the authors, changes associated by non-canonical MCL1 functions should happen under MCL1 inhibition and caspase inhibition, but not with MCL1 inhibition only.

The reviewer is on the right track, we expect non-canonical functions that occur under MCL-1 inhibition only to also occur under co-treatment with caspase inhibition. Our data with QVD shows that the cell death function of MCL-1 (i.e., inhibiting cell death effectors from initiating the caspase cascade) is not the main trigger of the phenotypes we report (cristae dysregulation and faPy acid oxidation disruption), however, cells without a functional cristae and/or defects in FAO, may not be able to survive long-term (please see our response to reviewer 1, comment 3). Thus, QVD treatment preserves these cells that may not survive the dismantling of such an essential structure. To confirm this, we have performed immunofluorescence of cleaved caspase 3 (**Figure 2 for reviewers**). These results show that, indeed, MCL-1 inhibition at the time points of our study does not result in increased Caspase-3 activation. We reported similar results of MCL-1 inhibition in oligodendrocyte precursor cells (Gil and Hanna et al., Glia, 2025, PMID: 41420072).

2. The authors show no data on the viability of the cells in response to the MCL1 inhibitor. To exclude secondary effects of the inhibitor, at least some of the results should be validated with an MCL1 knock down.

We thank the reviewer for this suggestion. We initially aPempted MCL-1 knockdown using five independent ASOs; however, knockdown efficiency was consistently poor, averaging less than 30% and highly variable, making these experiments uninterpretable. We recognize the importance of genetic validation and could pursue siRNA- mediated knockdown as an alternative strategy. This, however, would stray from the scope of this study, which focuses on the clinical relevance of pharmacological MCL-1 inhibition with BH3 mimetics.

3. In Figure 1, the authors show immunofluorescence data of mitochondria and nucleus staining and conclude that MCL1 inhibition alters mitochondrial morphology. Based on the images shown in Fig. 1, I do not think that individual mitochondria can be segmentd to measure their volume and length. In addition, some metrics such as mitochondrial content are not explained in the text or methods.

We can achieve mitochondrial segmentation with a SoRa Spinning Disk Confocal Microscope, which has a lateral (XY) resolution of approximately 120 nm to 150 nm and an axial (Z) resolution of approximately 300 nm–320 nm. All images are first denoised prior to being deconvolved using the Richardson-Lucy algorithm. Additionally, the FIB- SEM data are consistent with the IF data (both showing increase in mitochondria volume and surface area with no effect on the sphericity of the mitochondria network).

We agree with Reviewer 3 and added efforts to ensure defining our quantified parameters of mitochondrial morphology. In the initial submission, “mitochondrial content” referred to the count of individual mitochondrion instances per image z-stack. In the revised manuscript, however, we report mitochondria count normalized to total cell count for each individual image.

4. In Fig. 2 B-D, the authors show TEM and FIB-SEM imaging to demonstrate alterations in the cristae architecture upon treatment with MCL1 inhibitor. However, based on the images shown, it looks that cristae area and density is reduced under S63845 treatment in TEM images, while the FIB-SEM data come to the opposite conclusion. In addition, the quantification of cristae volume quantified as cristae volume in percentage is unclear to me.

We apologize for the confusion. No conclusions about the cristae area and density were made using the TEM data, as TEM data represent a single snapshot section of a mitochondrion without a discernible orientation. Cristae from TEM were described as “aberrant” and preliminarily revealed qualitative changes in cristae, and were followed up with FIB-SEM, 3D reconstruction of intact

© 2026. Published by The Company of Biologists under the terms of the Creative Commons Attribution License (https://creativecommons.org/licenses/by/4.0/).

mitochondria, and quantification of volume.

In the new version of the manuscript, we specify that the cristae volume is normalized to the volume of its respective mitochondria (i.e., how much of the mitochondrial volume is aPributed to cristae).

5. The change in CPT1/2 protein levels (Fig. 4) is interesting but does not directly proof that faHy acid oxidation is altered, as concluded by the authors. For this, the authors would need to directly measure faHy acid oxidation for example using Seahorse or metabolic tracing experiments. Also, to prove that the MCL1 inhibition affects neural differentiation through faHy acid oxidation, a rescue experiment should be performed through CPT1 overexpression.

We have optimized respirometry assays using Seahorse XF analysis and present our findings in **new Supplementary Figure 4** in the revised manuscript. Unfortunately, metabolic tracing experiments with labeled palmitate would be needed to draw conclusions about the direct effects of MCL-1 inhibition on faPy acid oxidation.

Regarding the CPT1 overexpression rescue experiment, we agree this would provide valuable mechanistic insight; however, given the complexity and scope of this experiment, we think it is an important point for future investigation.

6. In Figure 6, the authors show decreased intermediate progenitor cells ader MCL1 inhibition by immunofluorescence staining. I am not convinced that this can be concluded from the data shown, since the concentration of intermediate progenitor cells is very close to the noise levels. Since the MCL1 treated cells look much less sparse, I don't think the percentages can be compared (total counts are between 2-20). Although this data might give some indication that differentiation could be impaired, the measured effect could be very well due to lower viability of the cells. The authors need to control for this or come up with a different method for measuring differentiation.

We understand the comment and agree that the number of TBR2 intermediate progenitor cells is low, a well- recognized limitation of the differentiation. However, we disagree with the reviewer's assessment of noise levels. We focused on cells that express only TBR2 without PAX6 and rigorously examined this population with a blinded manual cell count. The percentages compared account for the normalized IPC count to the total nuclei (Hoechst) cell count. Moreover, the immunofluorescence images are complemented by RT-qPCR, which shows significant downregulation of *EOMES* (the gene encoding TBR2).

7. Figure 7 is missing quantification

We appreciate this comment and have now included this quantification in the revised version of the manuscript.

Reviewer #3 (Significance (Required)):

<u>General assessment</u>: The manuscript reports an interesting finding, which suggest a non-canonical role of MCL1 in mitochondrial remodeling, regulation of faHy acid oxidation and neuronal fate. While this finding would be highly interesting and relevant, the presented data do not sufficiently support this conclusion. Further experiments would have to be performed to proof causality.

<u>Advance</u>: Should the authors manage to proof their hypothesis by additional experiments, this would indeed advance the field on mitochondrial remodeling and its effect on neuronal differentiation by identifying a novel molecular player.
<u>Audience</u>: mitochondrial biology, cell biology, developmental neuroscience
<u>Own expertise</u>: mitochondrial biology, cell biology, advanced imaging techniques

3. Description of analyses that authors prefer not to carry out

© 2026. Published by The Company of Biologists under the terms of the Creative Commons Attribution License (https://creativecommons.org/licenses/by/4.0/).

Please include a point-by-point response explaining why some of the requested data or additional analyses might not be necessary or cannot be provided within the scope of a revision. This can be due to time or resource limitations or in case of disagreement about the necessity of such additional data given the scope of the study. Please leave empty if not applicable.

The authors show no data on the viability of the cells in response to the MCL1 inhibitor. To exclude secondary effects of the inhibitor, at least some of the results should be validated with an MCL1 knockdown.

We thank the reviewer for this suggestion. We initially aPempted MCL-1 knockdown using five independent ASOs; however, knockdown efficiency was consistently poor, averaging less than 30% and highly variable, making these experiments uninterpretable. We recognize the importance of genetic validation and could pursue siRNA-mediated knockdown as an alternative strategy. This, however, would fall outside the scope of this study, which focuses on the clinical relevance of pharmacological MCL-1 inhibition using BH3 mimetics.

While the authors "propose a model in which MCL-1 associates with MICOS", they do not offer direct scientific to support this hypothesis. Co-immunoprecipitation experiments or e.g. proximity ligation assays would beHer support the proposed model.

We performed proximity ligation assays and immunoprecipitation analyses to test for this interaction (**Figure 4 for reviewers**). The results indicate an interaction with MIC60, albeit very weak. To follow up, we would need to screen seven MICOS components, as well as OPA-1. The complexity of these analyses and their interpretation under endogenous conditions can make them difficult to interpret.

Brinkmann et al. (2025) also investigated cristae structure upon MCL-1 deletion in vivo and found no effect when MCL-1 was replaced with other Bcl-2 family members. It would be interesting to combine MCL-1 inhibition with overexpression of MCL-1 versus BCL-XL to reconsolidate some of the discrepant findings.

While this is a great suggestion for future studies, there are some complications. Specifically, it is likely that the inhibitor may also target the overexpressed MCL-1 and thus, a mutant form is needed. To address this, we generated a Flag-tagged MCL-1 construct with a mutated BH3 domain, previously described by Kotschy et al. Nature 2016. We validated the construct in HeLa cells, but unfortunately, the mutant protein appears to be significantly less stable than the WT construct, complicating analysis of this experiment.

NOTE: We have removed unpublished data that had been provided for the referees in confidence.

First decision letter

MS ID#: jcs.265172

MS TITLE: Pharmacological inhibition of MCL-1 disrupts mitochondrial cristae and depletes the human neural progenitor cell pool

AUTHORS: Marina Hanna; Madison Yarbrough; James Costanzo; Melanie Gil; Matthew Murrow; Vivian Gama
ARTICLE TYPE: Review Commons Transfer

Dear Dr Gama,

Many thanks for transferring your paper to Journal of Cell Science from Review Commons. Following initial assessment of the revisions, we have sought further input from the Review Commons

© 2026. Published by The Company of Biologists under the terms of the Creative Commons Attribution License (https://creativecommons.org/licenses/by/4.0/).

referees. I have now received their feedback. Please respond to the queries raised by the reviewer(s).

Further, please attend to all of the reviewers' comments and ensure that you clearly highlight all changes made in the revised manuscript. Please avoid using 'Tracked changes' in Word files as these are lost in PDF conversion. I should be grateful if you would also provide a point-by-point response detailing how you have dealt with the points raised by the reviewers in the 'Response to Reviewers' box. If you do not agree with any of their criticisms or suggestions please explain clearly why this is so.

Reviewer 1

SUMMARY OF THE ADVANCE MADE IN THIS PAPER AND ITS POTENTIAL SIGNIFICANCE TO THE FIELD

This study describes non-apoptotic roles of MCL-1 in human neural stem cells and connects MCL-1 inhibition to mitochondrial cristae formation and beta-oxidation. Connecting these roles to brain development, the authors also show a reduction in the number of progenitor cells upon MCL-1 inhibition, independently of caspase activity. This further links MCL-1 to a function in mitochondrial morphology maintenance and metabolism beyond its traditional role in apoptosis inhibition.

SUGGESTIONS TO AUTHORS

The authors have addressed all major concerns.

Reviewer 2

SUMMARY OF THE ADVANCE MADE IN THIS PAPER AND ITS POTENTIAL SIGNIFICANCE TO THE FIELD

Accumulating evidence indicates that MCL-1 regulates cellular processes beyond its well-established role in suppressing apoptosis. Despite these advances, the non-apoptotic functions of MCL-1 have remained largely unexplored in human primary cells. Addressing this important gap, Gama and colleagues demonstrated that pharmacologic inhibition of MCL-1 with the selective inhibitor S63845 in human neural progenitor cultures leads to marked defects in mitochondrial cristae organization, fatty acid oxidation, and neuronal differentiation. These findings were substantiated through multiple orthogonal approaches, including quantitative fluorescence microscopy, transmission electron microscopy (TEM), three-dimensional focused ion beam scanning electron microscopy (FIB-SEM), and biochemical analyses of protein expression.

The major strength of this manuscript is its focus on defining the non-apoptotic functions of MCL-1 in human primary neural progenitor cultures. By extending previous studies into a physiologically relevant human model, the work represents a timely and important advance in our understanding of MCL-1 biology.

SUGGESTIONS TO AUTHORS

Despite these strengths, the manuscript is limited by its largely descriptive nature. While the authors identify several phenotypes, these findings are primarily extensions of previous studies and provide only limited mechanistic insight into how MCL-1 inhibition gives rise to the observed effects. Most importantly, the study does not convincingly distinguish the reported mitochondrial and differentiation phenotypes from consequences of mitochondrial outer membrane permeabilization (MOMP) induced by MCL-1 inhibition. As a result, it remains unclear whether these phenotypes reflect bona fide non-apoptotic functions of MCL-1 or are secondary to MOMP and its downstream cellular effects.

Major Concerns

1. The experimental strategy does not convincingly distinguish non-apoptotic functions of MCL-1 from its canonical anti-apoptotic activity.

© 2026. Published by The Company of Biologists under the terms of the Creative Commons Attribution License (https://creativecommons.org/licenses/by/4.0/).

To separate the anti-apoptotic and non-apoptotic functions of MCL-1, the authors rely on the pan-caspase inhibitor Q-VD-OPh (QVD) in a subset of experiments. While QVD effectively prevents caspase activation and substrate cleavage downstream of apoptosis, it is not expected to block the upstream mitochondrial events triggered by MCL-1 inhibition. In the presence of S63845, BAX/BAK activation, mitochondrial outer membrane permeabilization (MOMP), cytochrome c release, and apoptosome assembly would still be predicted to occur despite caspase inhibition. Consequently, many of the reported phenotypes, particularly the alterations in mitochondrial ultrastructure, including cristae morphology, could arise as indirect consequences of MOMP rather than reflecting bona fide non-apoptotic functions of MCL-1. As currently presented, the data do not adequately distinguish between these possibilities, substantially weakening the central conclusion of the study.
2. Many of the reported mitochondrial phenotypes represent incremental advances.
Several phenotypes attributed to MCL-1 inhibition, including disruption of mitochondrial ultrastructure, accumulation of lipid droplets, and impaired fatty acid oxidation, have been described previously in other model systems. Although confirming these observations in human primary neural progenitors is valuable, the current work largely extends existing findings rather than providing fundamentally new biological insight. Greater mechanistic depth would be required to substantially advance the field.
3. The differentiation phenotype lacks mechanistic investigation.
The effects of MCL-1 inhibition on neural progenitor differentiation are among the most novel and potentially impactful findings in the manuscript. However, these observations remain largely descriptive. The study does not investigate how inhibition of the BH3-binding groove of MCL-1 mechanistically alters differentiation programs or identify the downstream pathways responsible for this phenotype. As a result, the biological significance of these observations remains uncertain.

Minor Concerns

1. Reliance on a single MCL-1 inhibitor raises concerns regarding specificity.
All experiments were performed using S63845. Although this is a well-characterized MCL-1 inhibitor, confirming key findings with an independent MCL-1 inhibitor would strengthen confidence that the observed phenotypes are not compound-specific. In addition, inclusion of a control BH3 mimetic that does not target MCL-1, such as venetoclax, would provide an important specificity control. If similar phenotypes were observed following BCL-2 inhibition, it would suggest that the effects are not unique to MCL-1 inhibition.

2. QVD is inconsistently incorporated throughout the study.
If the purpose of QVD is to isolate non-apoptotic functions of MCL-1, it should be incorporated consistently into experiments supporting the manuscript's central conclusions. Restricting QVD to only a subset of assays further limits the authors' ability to conclude that the reported phenotypes are independent of apoptosis. This does not obviate the major weakness of reliance on QVD (described above).
3. The evidence supporting displacement of ACSL1 from mitochondria is not sufficiently convincing.
The conclusion that MCL-1 inhibition displaces ACSL1 from mitochondria is based primarily on immunofluorescence imaging of exogenously expressed HALO-tagged ACSL1 (Figure 6). While intriguing, the presented images are not sufficiently compelling to support this conclusion, and several important controls are lacking. For example: 1) No biochemical validation (e.g., co-immunoprecipitation, mitochondrial fractionation, or proximity-labeling experiments) is provided to support altered MCL-1-ACSL1 association. 2) The functional competence of the ACSL1-HALO fusion protein is not demonstrated. 3) Appropriate negative controls for HALO staining are not shown. 4) The identity of the punctate structures and the diffuse HALO signal observed under control conditions remains unclear.
Although the quantitative analysis is consistent with the authors' interpretation, the imaging data alone provide relatively weak evidence for mitochondrial displacement of ACSL1.
4. The differentiation analysis in Figure 8 requires stronger supporting evidence.
The reported reduction in the $TBR2^{+}/PAX6^{-}$ intermediate progenitor population is difficult to evaluate from the presented images. QVD treatment alone appears to substantially alter cellular morphology, making interpretation challenging. In addition, the cultures are highly confluent, which further complicates quantitative assessment. Independent validation of the differentiation phenotype—for example through expression of lineage-specific markers by qPCR, RNA-seq, or immunoblotting—would substantially strengthen this conclusion.

© 2026. Published by The Company of Biologists under the terms of the Creative Commons Attribution License (https://creativecommons.org/licenses/by/4.0/).

Reviewer 3

SUMMARY OF THE ADVANCE MADE IN THIS PAPER AND ITS POTENTIAL SIGNIFICANCE TO THE FIELD

This paper describes a non-canonical role of the anti-apoptotic protein Myeloid Cell Leukemia-1 (MCL1) in regulating mitochondrial cristae structure, fatty acid metabolism and neuronal differentiation. This non-canonical function of MCL1 is novel and interesting and clearly significant for the field of mitochondrial biology, metabolism and neuronal differentiation.

SUGGESTIONS TO AUTHORS

The authors have performed several new experiments and analysis to strengthen their claims and have improved the clarity of the manuscript. Some limitations of this study remain, in particular the use of only one MCL-1 inhibitor without validation through MCL-1 knockout as well as the direct proof of altered fatty acid oxidation through palmitate tracing. However, I agree with the authors that these experiments are technically challenging and time-consuming, and can rather be performed in follow up projects. My remaining concerns and suggestions have been sufficiently addressed by the authors.

First revision

Author response to reviewers' comments

Reviewer 1: SUMMARY OF THE ADVANCE MADE IN THIS PAPER AND ITS POTENTIAL SIGNIFICANCE TO THE FIELD

This study describes non-apoptotic roles of MCL-1 in human neural stem cells and connects MCL-1 inhibition to mitochondrial cristae formation and beta-oxidation. Connecting these roles to brain development, the authors also show a reduction in the number of progenitor cells upon MCL-1 inhibition, independently of caspase activity. This further links MCL-1 to a function in mitochondrial morphology maintenance and metabolism beyond its traditional role in apoptosis inhibition.

SUGGESTIONS TO AUTHORS

The authors have addressed all major concerns.

We thank Reviewer 1 for their positive assessment of our manuscript and for acknowledging the significance of these findings. We are pleased that our revisions have addressed all of the reviewer's major concerns, and we appreciate the time and thoughtful evaluation they dedicated to our work.

Reviewer 2: SUMMARY OF THE ADVANCE MADE IN THIS PAPER AND ITS POTENTIAL SIGNIFICANCE TO THE FIELD

Accumulating evidence indicates that MCL-1 regulates cellular processes beyond its well-established role in suppressing apoptosis. Despite these advances, the non-apoptotic functions of MCL-1 have remained largely unexplored in human primary cells. Addressing this important gap, Gama and colleagues demonstrated that pharmacologic inhibition of MCL-1 with the selective inhibitor S63845 in human neural progenitor cultures leads to marked defects in mitochondrial cristae organization, fatty acid oxidation, and neuronal differentiation. These findings were substantiated through multiple orthogonal approaches, including quantitative fluorescence microscopy, transmission electron microscopy (TEM), three-dimensional focused ion beam scanning electron microscopy (FIB-SEM), and biochemical analyses of protein expression.

The major strength of this manuscript is its focus on defining the non-apoptotic functions of MCL-1 in human primary neural progenitor cultures. By extending previous studies into a physiologically relevant human model, the work represents a timely and important advance in our understanding of MCL-1 biology.

© 2026. Published by The Company of Biologists under the terms of the Creative Commons Attribution License (https://creativecommons.org/licenses/by/4.0/).

We thank Reviewer 2 for insightful review and for recognizing the significance of extending MCL-1's non-apoptotic functions into a physiologically relevant human model, and for highlighting the strength of our multi-modal approach combining quantitative fluorescence microscopy, TEM, FIB-SEM, and biochemical analyses. We address their specific concerns below:

SUGGESTIONS TO AUTHORS
Despite these strengths, the manuscript is limited by its largely descriptive nature. While the authors identify several phenotypes, these findings are primarily extensions of previous studies and provide only limited mechanistic insight into how MCL-1 inhibition gives rise to the observed effects. Most importantly, the study does not convincingly distinguish the reported mitochondrial and differentiation phenotypes from consequences of mitochondrial outer membrane permeabilization (MOMP) induced by MCL-1 inhibition. As a result, it remains unclear whether these phenotypes reflect bona fide non-apoptotic functions of MCL-1 or are secondary to MOMP and its downstream cellular effects.

Major Concerns
1. The experimental strategy does not convincingly distinguish non-apoptotic functions of MCL-1 from its canonical anti-apoptotic activity. To separate the anti-apoptotic and non-apoptotic functions of MCL-1, the authors rely on the pan-caspase inhibitor Q-VD-OPh (QVD) in a subset of experiments. While QVD effectively prevents caspase activation and substrate cleavage downstream of apoptosis, it is not expected to block the upstream mitochondrial events triggered by MCL-1 inhibition. In the presence of S63845, BAX/BAK activation, mitochondrial outer membrane permeabilization (MOMP), cytochrome c release, and apoptosome assembly would still be predicted to occur despite caspase inhibition. Consequently, many of the reported phenotypes, particularly the alterations in mitochondrial ultrastructure, including cristae morphology, could arise as indirect consequences of MOMP rather than reflecting bona fide non-apoptotic functions of MCL-1. As currently presented, the data do not adequately distinguish between these possibilities, substantially weakening the central conclusion of the study.

We thank the reviewer for raising these important points, which highlight a genuine limitation in our experimental strategy. We agree that QVD, by acting downstream of caspase activation, cannot formally rule out upstream MOMP-associated events such as BAX/BAK activation or cytochrome c release. We agree that MOMP is generally considered a committed step toward cell death; however, emerging evidence indicates that MOMP is not always an all-or-none, uniformly lethal event. Live-cell imaging studies have shown that MOMP can be incomplete within a cell, with a subset of mitochondria remaining intact, and that the persistence of this intact mitochondrial pool correlates with cellular recovery when caspase activity is blocked (Ichim et al., 2015, PMID: 25702873). This indicates that caspase-inhibited cells are not necessarily locked into a uniform, unrecoverable MOMP state, and that a functional mitochondrial population, along with the cellular processes it supports, can persist under these conditions.

The best approach to answer this critical point raised by Reviewer 2 is to perform analysis in a BAX/BAK double knockout (DKO) background. Unfortunately, we have previously shown that BAX/BAK double-knockout human iPSC-derived cortical cells, which cannot undergo MOMP, display abnormal mitochondrial morphology and give rise to aberrant cortical structures (Joshi et al., 2020, PMID: 32978370). This finding demonstrates, independently of any consideration of MOMP kinetics, that mitochondrial structural integrity can be perturbed by the core apoptotic machinery in a manner separable from cell death itself, and supports the plausibility that the cristae and mitochondrial ultrastructural phenotypes we observe upon MCL-1 inhibition reflect a function distinct from MOMP, rather than a downstream consequence of it. This work also presents a critical limitation of using the DKO model, because the mitochondria are already perturbed. Together, these lines of evidence support our interpretation that the phenotypes reported here are unlikely to solely reflect an ongoing, unresolved MOMP process. Although we included these points in the previous version of the paper, we agreed with Reviewer 2 on the importance of expanding them in the Discussion section and incorporating them more explicitly (lines 393–413).

2. Many of the reported mitochondrial phenotypes represent incremental advances. Several phenotypes attributed to MCL-1 inhibition, including disruption of mitochondrial ultrastructure, accumulation of lipid droplets, and impaired fatty acid oxidation, have been described previously in

© 2026. Published by The Company of Biologists under the terms of the Creative Commons Attribution License (https://creativecommons.org/licenses/by/4.0/).

other model systems. Although confirming these observations in human primary neural progenitors is valuable, the current work largely extends existing findings rather than providing fundamentally new biological insight. Greater mechanistic depth would be required to substantially advance the field.

We agree that individual features of the phenotype we describe, such as cristae disruption or lipid droplet accumulation, have precedent in other systems. We would like to respectfully highlight, however, that this study's significance lies in demonstrating that these phenotypes occur in a coordinated manner in human primary neural progenitor cells, a physiologically relevant and technically demanding model that has not previously been used to interrogate non-apoptotic MCL-1 function. By confirming and extending these findings in a primary human context and directly linking them to a developmentally relevant outcome (progenitor differentiation), our study provides a critical bridge between mechanistic work in simplified systems and MCL-1 function in a setting directly relevant to human neurogenesis. We revised the Introduction (lines 121-126) and Discussion (lines 380 - 384) to more clearly articulate this distinction and the added value of the human cell developmental model.

3. The differentiation phenotype lacks mechanistic investigation. The effects of MCL-1 inhibition on neural progenitor differentiation are among the most novel and potentially impactful findings in the manuscript. However, these observations remain largely descriptive. The study does not investigate how inhibition of the BH3-binding groove of MCL-1 mechanistically alters differentiation programs or identify the downstream pathways responsible for this phenotype. As a result, the biological significance of these observations remains uncertain.

We agree with the reviewer that identifying the downstream pathways linking MCL-1 inhibition to altered differentiation would substantially strengthen the mechanistic depth of the current study. As the reviewer notes, this is among the most novel findings of our work, and we agree that detailed pathway dissection, for example through transcriptomic profiling or targeted pathway inhibition, is an important direction for follow-up studies. We consider this mechanistic dissection to be beyond the scope of the current manuscript, but included this critical point in the Discussion section (lines 465 – 470)

Minor Concerns

1. Reliance on a single MCL-1 inhibitor raises concerns regarding specificity. All experiments were performed using S63845. Although this is a well-characterized MCL-1 inhibitor, confirming key findings with an independent MCL-1 inhibitor would strengthen confidence that the observed phenotypes are not compound-specific. In addition, inclusion of a control BH3 mimetic that does not target MCL-1, such as venetoclax, would provide an important specificity control. If similar phenotypes were observed following BCL-2 inhibition, it would suggest that the effects are not unique to MCL-1 inhibition.

We thank Reviewer 2 for raising this point regarding specificity. S63845 is among the most extensively characterized and selective MCL-1 inhibitors available, with well-documented on-target activity and minimal cross-reactivity with other BCL-2 family members. Kotschy et al. originally characterized S63845 as binding human MCL-1 with high affinity (Kd of 0.19 nM) and showing no discernible binding to the other BCL-2 family members, BCL-2 or BCL-XL, in contrast to earlier-generation inhibitors such as A-1210477 (Kotschy et al., 2016, PMID: 27760111). This selectivity has since been independently confirmed by our group and others (Rasmussen et al., 2020, PMID: 32283523 and Greaves et al., 2019, PMID: 30185825), who reported a high binding affinity and selectivity of S63845 in inducing apoptosis in human iPSC-derived cardiomyocytes and MCL-1-dependent cancer cell lines, respectively, supporting the specificity of the phenotypes we observe in our system.

2. QVD is inconsistently incorporated throughout the study. If the purpose of QVD is to isolate non-apoptotic functions of MCL-1, it should be incorporated consistently into experiments supporting the manuscript's central conclusions. Restricting QVD to only a subset of assays further limits the authors' ability to conclude that the reported phenotypes are independent of apoptosis. This does not obviate the major weakness of reliance on QVD (described above).

© 2026. Published by The Company of Biologists under the terms of the Creative Commons Attribution License (https://creativecommons.org/licenses/by/4.0/).

We incorporated QVD into experiments where we considered caspase-dependent effects most likely to confound interpretation; in other assays, we omitted it because of factors such as assay duration, compatibility with downstream readouts, or prior validation showing that its inclusion did not alter outcomes in related experiments. We added a statement in the Limitations section (lines 550-554) to state these points more explicitly.

3. The evidence supporting displacement of ACSL1 from mitochondria is not sufficiently convincing. The conclusion that MCL-1 inhibition displaces ACSL1 from mitochondria is based primarily on immunofluorescence imaging of exogenously expressed HALO-tagged ACSL1 (Figure 6). While intriguing, the presented images are not sufficiently compelling to support this conclusion, and several important controls are lacking. For example: 1) No biochemical validation (e.g., co-immunoprecipitation, mitochondrial fractionation, or proximity-labeling experiments) is provided to support altered MCL-1-ACSL1 association. 2) The functional competence of the ACSL1-HALO fusion protein is not demonstrated. 3) Appropriate negative controls for HALO staining are not shown. 4) The identity of the punctate structures and the diffuse HALO signal observed under control conditions remains unclear. Although the quantitative analysis is consistent with the authors' interpretation, the imaging data alone provide relatively weak evidence for mitochondrial displacement of ACSL1.

We thank the reviewer for this detailed critique. We agree that the imaging data in Figure 6, based on exogenously expressed HALO-tagged ACSL1, are suggestive rather than definitive; accordingly, we have tempered the language throughout the Results and Discussion. We also addressed the specific limitations raised by Reviewer 2 as open questions in the Discussion section (lines 430 - 434).

4. The differentiation analysis in Figure 8 requires stronger supporting evidence. The reported reduction in the $TBR2^{+}/PAX6^{-}$ intermediate progenitor population is difficult to evaluate from the presented images. QVD treatment alone appears to substantially alter cellular morphology, making interpretation challenging. In addition, the cultures are highly confluent, which further complicates quantitative assessment. Independent validation of the differentiation phenotype—for example through expression of lineage-specific markers by qPCR, RNA-seq, or immunoblotting—would substantially strengthen this conclusion.

We thank the reviewer for highlighting these concerns regarding the interpretability of the imaging-based quantification in Figure 8. We have not measured the effects of QVD treatment alone on cellular morphology. However, high culture confluency is necessary to maintain neural progenitor cells in good condition. In Supplementary Figure 5, we show decreased expression of the gene encoding TBR2 (*EOMES*) after 48 hours of treatment with MCL-1i, with and without QVD. We clarified this finding in lines 356-359 of the Results section.

Reviewer 3: SUMMARY OF THE ADVANCE MADE IN THIS PAPER AND ITS POTENTIAL SIGNIFICANCE TO THE FIELD

This paper describes a non-canonical role of the anti-apoptotic protein Myeloid Cell Leukemia-1 (MCL1) in regulating mitochondrial cristae structure, fatty acid metabolism and neuronal differentiation. This non-canonical function of MCL1 is novel and interesting and clearly significant for the field of mitochondrial biology, metabolism and neuronal differentiation.

SUGGESTIONS TO AUTHORS

The authors have performed several new experiments and analysis to strengthen their claims and have improved the clarity of the manuscript. Some limitations of this study remain, in particular the use of only one MCL-1 inhibitor without validation through MCL-1 knockout as well as the direct proof of altered fatty acid oxidation through palmitate tracing. However, I agree with the authors that these experiments are technically challenging and time-consuming, and can rather be performed in follow up projects. My remaining concerns and suggestions have been sufficiently addressed by the authors.

We thank the reviewer for their positive evaluation of our manuscript and for recognizing the novelty and significance of these findings for the fields of mitochondrial biology, metabolism, and

© 2026. Published by The Company of Biologists under the terms of the Creative Commons Attribution License (https://creativecommons.org/licenses/by/4.0/).

neuronal differentiation. We appreciate the reviewer's acknowledgment of the technical challenges associated with MCL-1 knockout validation and direct palmitate tracing experiments. As the reviewer notes, we agree these represent important directions for follow-up work, and we plan to pursue them in future studies building on the findings presented here. To address this point, we added it to the Limitations section (lines 554–557).

Second decision letter

MS ID#: jcs.265172R1

MS Title: Pharmacological inhibition of MCL-1 disrupts mitochondrial cristae and depletes the human neural progenitor cell pool

Authors: Marina Hanna; James Costanzo; Madison Yarbrough; Melanie Gil; Matthew Murrow; Vivian Gama
Article Type: Review Commons Transfer

Dear Dr Gama,

I am happy to tell you that your manuscript has been accepted for publication in Journal of Cell Science, pending standard publication integrity checks.

Thank you for sending your manuscript to Journal of Cell Science through Review Commons.

© 2026. Published by The Company of Biologists under the terms of the Creative Commons Attribution License (https://creativecommons.org/licenses/by/4.0/).
